# Efficacy and Safety of Postmenopausal Osteoporosis Treatments: A Systematic Review and Network Meta-Analysis of Randomized Controlled Trials

**DOI:** 10.3390/jcm10143043

**Published:** 2021-07-08

**Authors:** Shih-Yin Lin, Min-Chih Hung, Shih-Fu Chang, Fon-Yih Tsuang, Jenny Zwei-Chieng Chang, Jui-Sheng Sun

**Affiliations:** 1 Department of Dentistry, MacKay Memorial Hospital, No. 92, Sec. 2, Zhongshan N. Rd., Taipei 10449, Taiwan; aaa1212p@gmail.com; 2 School of Dentistry, College of Medicine, National Taiwan University, No. 1, Chang-De Street, Taipei 10048, Taiwan; orthodaniel@yahoo.com.tw (M.-C.H.); blr1350@gmail.com (S.-F.C.); 3 Division of Neurosurgery, Department of Surgery, National Taiwan University Hospital, No. 7, Chung-Shan South Road, Taipei 10002, Taiwan; tsuangfy@gmail.com; 4 Department of Orthopedic Surgery, College of Medicine, China Medical University, No. 2, Yu-Der Rd., Taichung 40447, Taiwan; 5 Department of Orthopedic Surgery, National Taiwan University Hospital, No. 7, Chung-Shan South Road, Taipei 10002, Taiwan

**Keywords:** network meta-analysis, randomized controlled trial, osteoporosis, bone mineral density, risks of complications

## Abstract

Although a range of pharmacological interventions is available, it remains uncertain which treatment for osteoporosis is more effective. This network meta-analysis study aimed to compare different drug efficacy and safety in randomized controlled trials (RCTs) for the treatment of postmenopausal osteoporosis. PubMed, EMBASE, MEDLINE, Clinicaltrial.gov, Cochrane library, Google scholar were searched up to 31 October 2020. Randomized placebo-controlled trials that reported measures of bone mineral density (BMD) percentage change and/or numbers of adverse events of postmenopausal osteoporosis patients were included. Network meta-analysis was conducted using frequentist approach. Ninety-four RCTs comprising 15,776 postmenopausal osteoporosis females were included in the network meta-analysis. Compared with placebo, most interventions showed increase in BMD change. According to surfaces under the cumulative ranking curves (SUCRAs), strontium ranelate, fluoride, and hormone replacement therapy were most effective in increasing total hip, lumbar spine, and distal radius BMD, respectively. Parathyroid hormone (PTH) was most effective in preventing new hip fracture. When taking into account all anatomic sites, bisphosphonate (BP), monoclonal antibody (mAb), and fluoride have a balanced efficacy in increasing BMD at all sites. Considering both the effectiveness of increasing BMD and preventing hip fracture, mAb, BP, and PTH are more favorable among all interventions. The treatment effects of different medications on BMD percentage change are anatomic site-dependent. After weighing anti-osteoporosis treatment efficacy against risk of complications, BP and mAb are the more favorable interventions to increase BMD at all sites and reduce the risks of hip fracture and death.

## 1. Introduction

Osteoporosis is a common metabolic bone disease and major worldwide public health problem that leads to an increase in bone fragility and susceptibility to fracture [1]. The osteoporosis-related fractures are complicated with significant morbidity and mortality [2,3,4] and provoke a heavy economic burden for both patients and society [5,6,7]. Because the decrease in estrogen production accelerates bone loss, osteoporosis is quite common in postmenopausal women [8,9,10].

Hip fractures are the greatest complication since they may influence the baseline functionality (Barthel index) of osteoporosis patients [11]. Patients with osteoporosis-related fractures are at a higher risk of subsequent re-fractures and are associated with increased morbidity and premature mortality [12]. The anti-osteoporosis medication taken for a year or more reduces the incidence of re-fractures by 70% [13]. Fracture Liaison Service (FLS) care also significantly reduces the post-fracture mortality, especially in hip fractures [12]. Bisphosphonates (BP) treatment is associated with reduced mortality in women after hip fracture and concomitantly decreases mortality [14].

In recent FLS cohort study, significantly more deaths were observed within three years of initial fracture after fracture; but a higher rate of medication initiation/review was associated with a lower rate of re-fracture [15]. Current prevention and treatment of postmenopausal osteoporosis mainly consist of bisphosphonates, hormone replacement therapy, denosumab, strontium ranelate, and selective estrogen receptor modulators [16]. However, it remains uncertain which therapy is more effective and not all currently available therapies allow for long-term use. For instance, the increased duration of bisphosphonates therapy may result in osteonecrosis of the jaw, atypically low-impact subtrochanteric stress fractures, or gastrointestinal disturbances [17,18,19]. Inconsistent therapeutic effect on specific treatment modality has also been reported [20,21]. Until now, very few randomized controlled trials reported complications in the treatment of postmenopausal osteoporosis.

An effective approach with a favorable long-term safety to prevent bone loss and reduce fracture risk is mandatory for current osteoporosis treatment [22]. Previous studies for treatment of postmenopausal osteoporosis only focused on certain classifications of medicines and lacked the comparison between their long-term adverse effects and treatment efficacy. Since many treatments have not been directly compared, traditional meta-analyses are not able to generate clear hierarchies among available treatments. We systematically reviewed randomized controlled trials (RCTs) and carried out a network meta-analysis to determine which effective osteoporosis treatments exhibited fewer side effects. We aimed to comprehensively compare and rank different drug efficacy and safety with a network meta-analysis of RCTs in the treatment of postmenopausal osteoporosis.

## 2. Materials and Methods

Network meta-analysis is an extension of pairwise meta-analysis to allow indirect comparisons of multiple interventions that have not been examined in head-to-head studies. In comparison with traditional pairwise meta-analyses, network meta-analysis provides a larger amount of evidence, the relative effectiveness, and ranking among all interventions. Until very recently, few network meta-analyses have attempted to compare the relative efficacies and/or safety of multiple therapies in postmenopausal osteoporosis treatment [23,24,25,26,27]. Here, we conducted this systematic review and network meta-analysis according to the preferred reporting items for systematic reviews and meta-analysis (PRISMA) guidelines [28] and the Cochrane Handbook for Systematic Reviews of Interventions (Available at: www.cochrane-handbook.org, accessed on 15 November 2020). Since all analyses were based on previous published studies, ethical approval was not necessary.

### 2.1. Literature Search and Selection Criteria

#### 2.1.1. Search Strategy

PubMed, EMBASE, MEDLINE, ClinicalTrial.gov, the Cochrane library, and Google scholar were searched up to 31 October 2020 for randomized placebo-controlled trials of postmenopausal osteoporosis treatments using defined terms (Supplementary Table S1). Additional eligible trials in the reference lists of meta-analyses, reviews, and retrieved trials of postmenopausal osteoporosis treatments were also identified manually. In a systematic review and meta-analysis for evaluation of fragility fractures, the authors have found that although magnetic resonance imaging is sensitive and specific for osteoporosis, its use for screening has not been sufficiently evaluated. While computed tomography may predict fracture occurrence, its cost, radiation exposure, and availability have made it unsuitable for screening. Whereas ultrasound appears to be a good predictor of fracture occurrence, due to operator-dependency and difficulty in standardization of testing it is not able to replace DXA as a screening tool for osteoporosis. Therefore, DXA remains to be the better screening method to evaluate osteoporosis and predict fragility fractures [29].

#### 2.1.2. Selection Criteria

Studies: randomized, placebo-controlled trials analyzed by intention to treat that reported measures of BMD percentage change and numbers of adverse events were included. Combination drug therapy and nutritional supplement were excluded in this study.

#### 2.1.3. Population

Participants were postmenopausal women previously untreated and clinically diagnosed as osteoporosis objectively with dual-energy X-ray absorptiometry (DXA). The exclusion criteria were (1) men; (2) secondary osteoporosis caused by certain medical conditions or treatments, such as metastasis, Paget’s disease, hypercalcemia, or glucocorticoid-induced osteoporosis; (3) previous postmenopausal osteoporosis treatment with continuous medication; (4) studies with asymptomatic or healthy general population as control.

#### 2.1.4. Interventions

We intended to include studies treated with any pharmacologic therapy (bisphosphonate, estrogen-progestin, selective estrogen-receptor modulators, parathyroid hormone, calcitonin, monoclonal antibody, sodium fluoride, strontium ranelate, beta-blocker, diuretic, statin, nitrate), and control (placebo).

#### 2.1.5. Comparisons

Postmenopausal osteoporosis patients treated with any of the above treatments compared to any other treatment regimen or control.

#### 2.1.6. Outcomes

Primary outcomes included percentage change in bone mineral density (BMD) from baseline at lumbar spine (LS), total hip (TH), or distal radius (RU) after postmenopausal osteoporosis treatments. The incidence of adverse events including cancer, cardiovascular disease (CVD), hip fracture, death, and osteonecrosis of the jaw.

### 2.2. Data Abstraction and Quality Assessment

Two independent reviewers (S-YL and S-FC) initially screened titles and abstracts to identify potentially applicable studies. Full-text articles that met inclusion criteria were then accessed for data extraction, adverse events, patients’ demographics, medicine, treatment protocol, duration of follow-up, outcome measurements of BMD and incidence of adverse events. Studies would be cited under one study name and only the most inclusive data were obtained if same data were reported in multiple studies. Consensus was achieved by discussion with a third reviewer (JZC) when there was disagreement between the two reviewers. Selection bias, performance bias, detection bias, attrition bias, and reporting bias of the included studies were assessed and classified according to Cochrane guidelines and recorded as low, unclear, or high risk of bias for quality assessment.

### 2.3. Statistical Methods

The effect size was odds ratio (OR) for dichotomous outcomes or weighted mean differences (WMDs) for continuous outcomes with a 95% confidence interval (95% CI). Traditional pairwise meta-analysis was performed using random-effects models with DerSimonian and Laird weights for direct comparisons. Heterogeneity within each pairwise comparison was tested using the Cochran Q statistic and quantified with the *I*^2^ statistic. Small study bias and/or publication bias were assessed with Funnel plot analysis and Egger’s test. For comparison of multiple treatments, network meta-analysis was performed within a frequentist framework [30]. Design-by-treatment interaction, loop inconsistency, and side-splitting models were tested to evaluate the inconsistency between direct and indirect evidence within the network. The surfaces under the cumulative ranking curves (SUCRAs) were calculated to rank treatments for each independent outcome. Stata (version SE15.0, StataCorp, College Station, TX, USA) software package was used for all statistical analyses with significance level set at 5%. Treatment hierarchy considering simultaneously BMD outcomes at the three different sites or total hip BMD versus incidence of hip fracture was summarized in clustered ranking plots based on cluster analysis of the SUCRA values.

## 3. Results

### 3.1. Search Results

**Figure 1:**
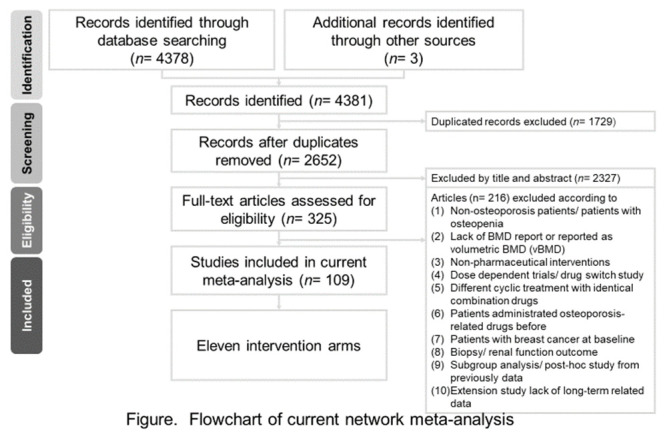
Flow diagram of this network meta-analysis.

The selection process was documented in a PRISMA flowchart (Figure 1). A total of 4378 studies were identified through database searching and three additional studies detected manually. After exclusion of 1729 duplicated studies, another 2461 trials were excluded during screening of the title and the abstract, resulting in 191 papers for full-text evaluation. Eventually, 98 articles met the inclusion criteria for this review. The excluded 93 studies at full-text stage were listed in (Supplementary Table S2) with reasons. The characteristics of the included 98 studies were detailed in Supplementary Tables S3 and S4. From these 98 trials, 32 did not report years since menopause, 30 did not report BMI, and 30 gave description of concerned complications.

**Figure 2:**
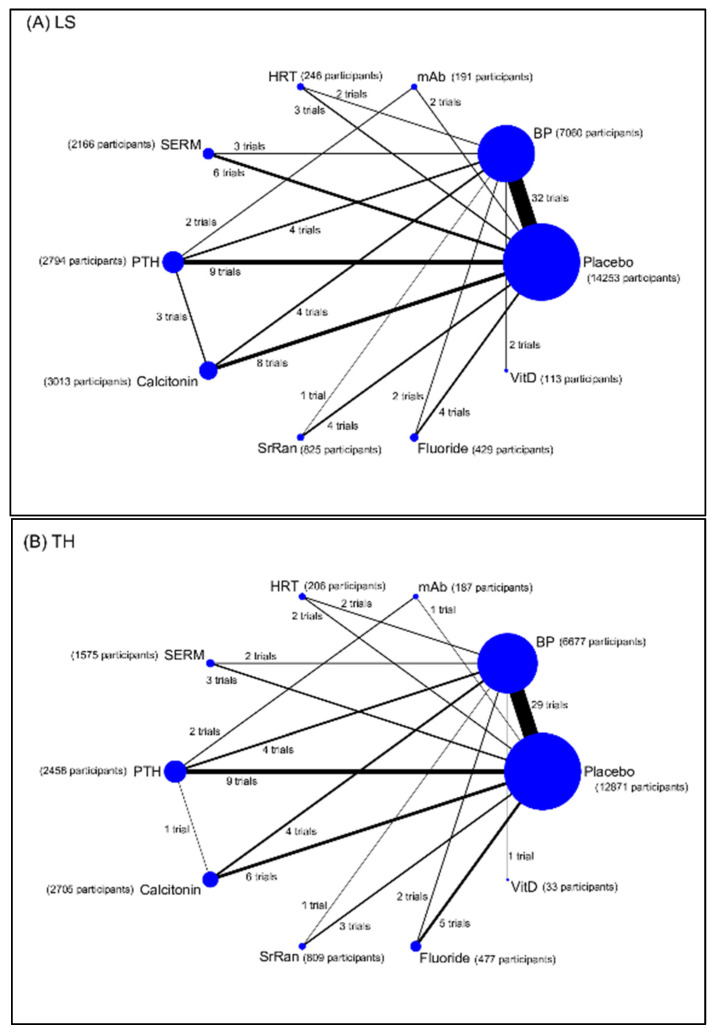

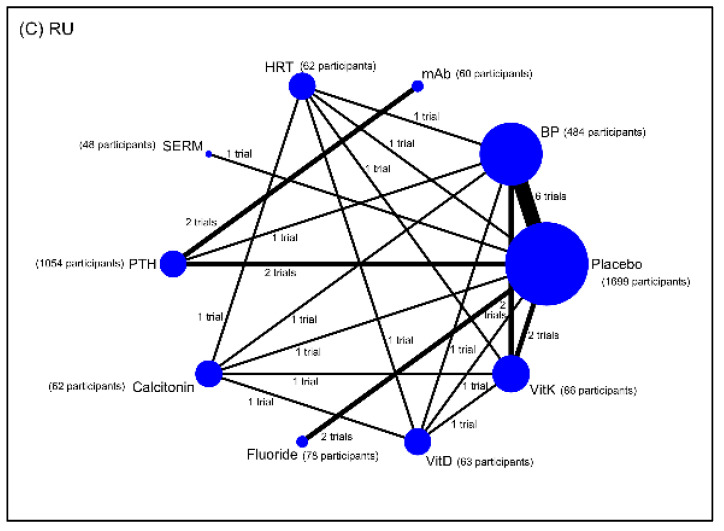
Networks of treatment comparisons for percentage change in bone mineral density (BMD) from baseline at (**A**) lumbar spine (LS), (**B**) total hip (TH), and (**C**) radius (RU) in postmenopausal women with osteoporosis. Abbreviation: BP, bisphosphonate; HRT, hormone replacement therapy; mAb, monoclonal antibody; PTH, parathyroid hormone; SERM, selective estrogen receptor modulator; SrRan, strontium ranelate; Vit D, vitamin D; Vit K, vitamin K.

**Figure 3:**
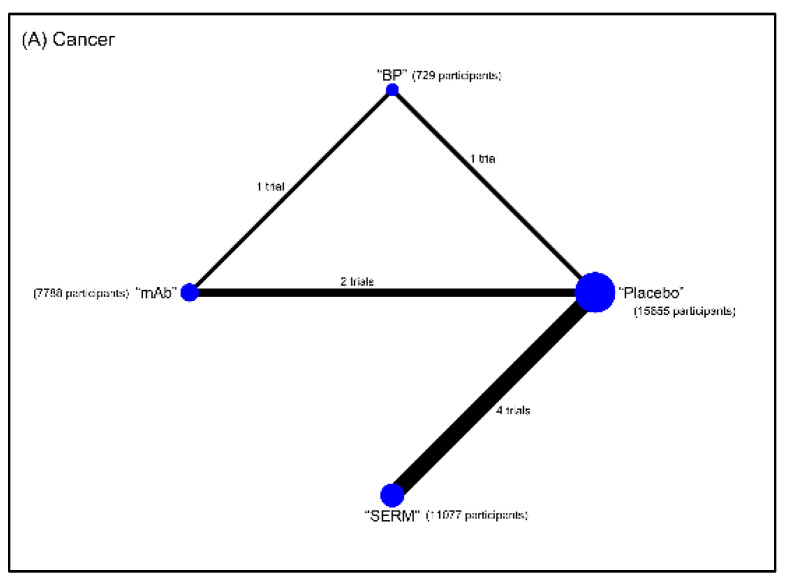

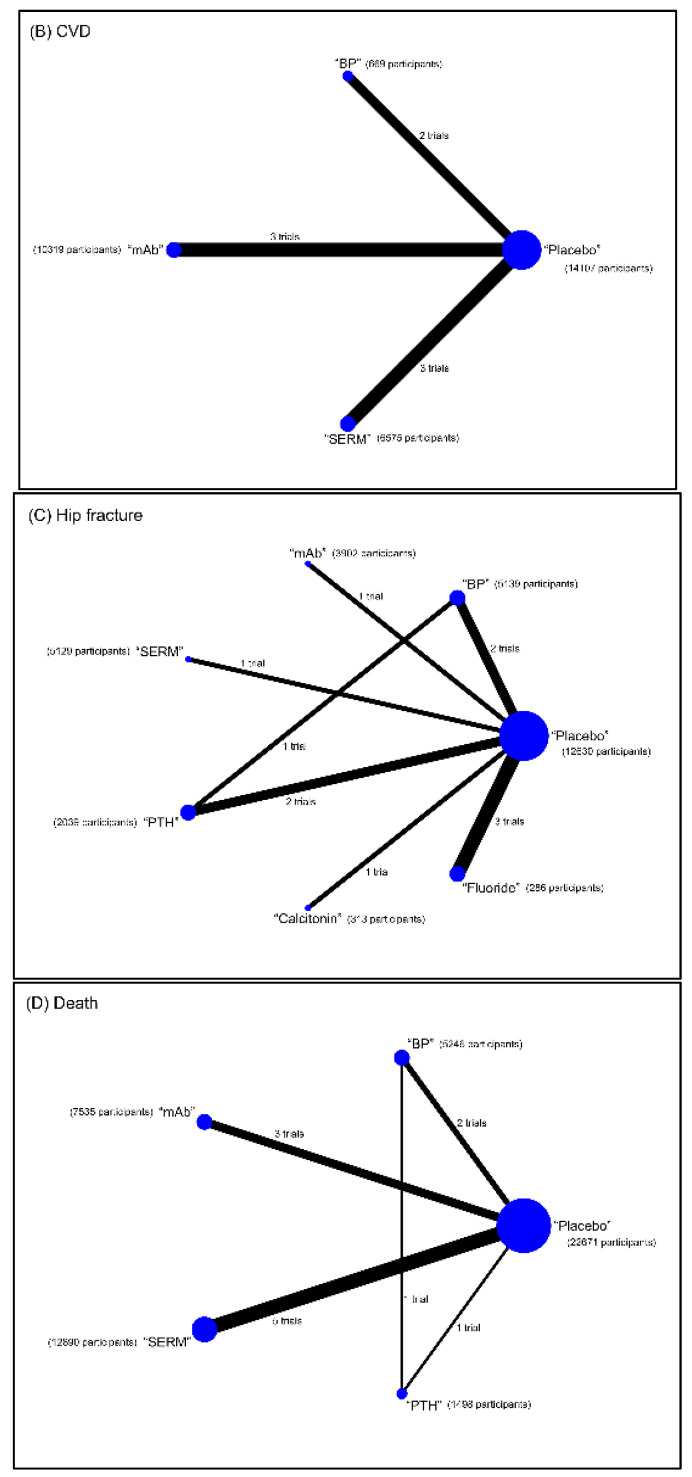
Networks of treatment comparisons for the incidence of (**A**) cancer, (**B**) cardiovascular disease (CVD), (**C**) hip fracture, and (**D**) death in postmenopausal women with osteoporosis. Abbreviation: BP, bisphosphonate; mAb, monoclonal antibody; PTH, parathyroid hormone; SERM, selective estrogen receptor modulator.

Eleven interventions were identified and classified according to types of pharmacological mechanisms: placebo, bisphosphonate (BP), hormone replacement therapy (HRT; such as estrogen-progestin), selective estrogen-receptor modulators (SERMs), parathyroid hormone (PTH), calcitonin, monoclonal antibody (mAb; such as denosumab, a RANKL inhibitor), sodium fluoride, strontium ranelate (SrRan), vitamin D (VitD), and vitamin K (VitK). The BMD measurements were grouped by anatomic sites into lumbar spine BMD (LS), total hip BMD (TH), and distal radius BMD (RU) groups. The reported complications that we were interested involved the number of hip fracture, cancer, cardiovascular disease (CVD), death, and osteonecrosis of the jaw (ONJ). Network maps of direct evidence for BMD (Figure 2) and adverse events (Figure 3) were presented with number of trials and participants for each intervention. The participants primarily received placebo or BP. Since only RCTs were included in this study, the risk of bias was generally low (Supplementary Figure S1). The results of conventional pairwise meta-analysis for BMD and adverse events were shown in forest plots in Supplementary Figure S2 and Supplementary Figure S3, respectively.

### 3.2. Results of the Network Meta-Analysis

Table 1A shows the results of network meta-analysis for BMD percentage change at LS. In comparison with placebo, all interventions showed statistically significant increase in BMD percentage change except for VitD. Fluoride was most effective in increasing BMD (9.45 (weighted mean difference; WMD); 95% confidence interval (CI): 6.89–12.01). PTH, mAb, SrRan, and BP were the more effective treatments; while HRT, SERM, calcitonin, and Vit D were less effective in increasing BMD at LS.

Table 1B shows the results of network meta-analysis for BMD percentage change at TH. In comparison with placebo, all interventions showed statistically significant increase in BMD percentage change except for VitD. SrRan (5.65; 3.83–7.46) was most effective in increasing BMD at TH followed by mAb, HRT, BP, and PTH. The less effective treatments were SERM, fluoride, calcitonin and VitD.

Table 1C shows the results of network meta-analysis for BMD percentage change at RU. When compared with placebo, HRT (5.04; 1.93–8.14) was most effective in increasing BMD followed by calcitonin and BP. Although fluoride, mAb, VitK, and SERM appeared to be more effective while VitD seemed less effective than placebo, these results were not statistically significant; whereas PTH (−2.40; −4.65 to −0.15) was significantly less effective than placebo to increase BMD at RU.

Table 2A–D shows the results of network meta-analysis for the incidence of adverse events. SERM (OR: 0.65; 95% CI: 0.48–0.88) exhibited significantly decreased risk of cancer than placebo while BP and mAb increased the risk. SERM showed less, BP showed equivalent, while mAb showed more risk than placebo for CVD to occur. Compared with placebo, mAb and BP showed decreased incidence rate of death; while SERM and PTH increased the risk of death. Nonetheless, all of the above-mentioned comparisons were statistically insignificant expect for the comparison between SERM and placebo for incidence of cancer. As for the incidence of hip fractures, all interventions were associated with decreased risk of new fractures than placebo. However, the relative beneficial effects were statistically significant only when treated with mAb (0.60; 0.37–0.98) or BP (0.58; 0.42–0.82).

Only five trials intended to investigate the complication of ONJ (Table 3A). Three studies reported zero event of ONJ after one to three-year follow-up [31,32,33]. One study reported one event of ONJ after 12 months of romosozumab (mAb) treatment and zero for placebo [34]. The other was a seven-year extension study of an originally three-year randomized, double-blind, placebo controlled trial where the original placebo group had received seven additional years of denosumab while the original experimental group had received 10 total years of denosumab [35]. Therefore, quantitative meta-analysis was inapplicable for the incidence of ONJ. As different interventions may be associated with risks for different cancers or reasons of death, we have listed the associated types of cancers or reasons of death in Table 3B,C.

### 3.3. Ranking of Interventions

**Figure 4:**
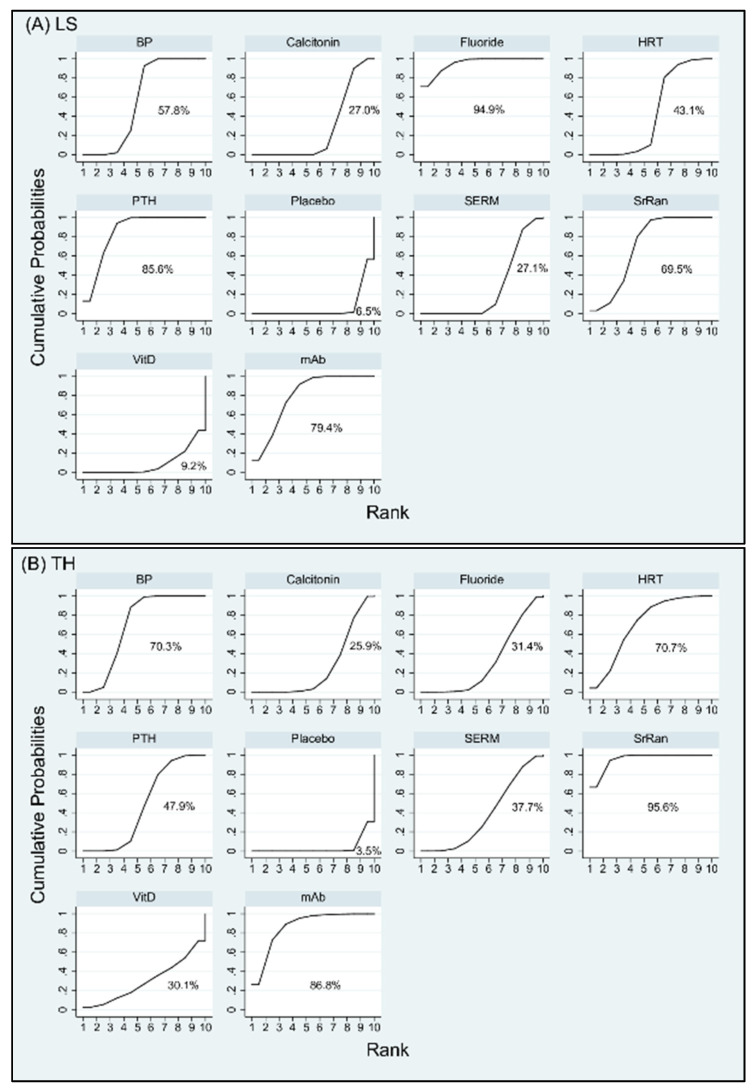

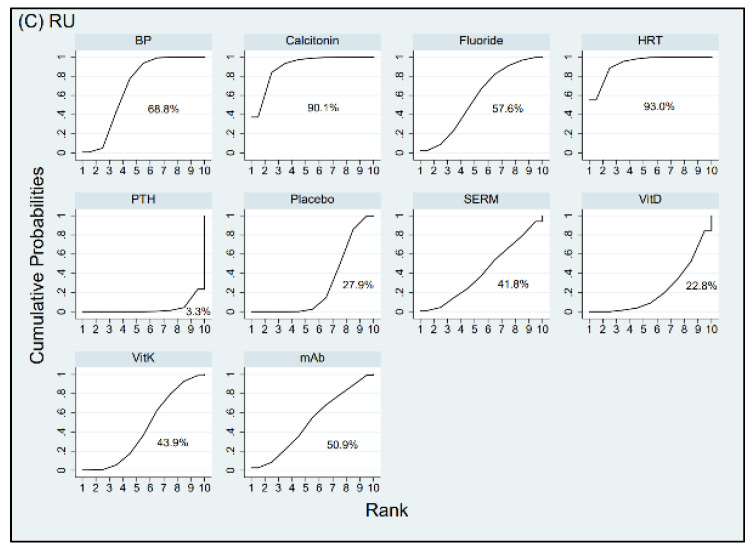
Cumulative rankograms: plots of the surface under the cumulative ranking curves (SUCRAs) for the increase in bone mineral density (BMD) at (**A**) lumbar spine (LS), (**B**) total hip (TH), and (**C**) radius (RU) with various treatments in the postmenopausal osteoporosis networks. Ranking indicates the probability to be the best treatment, the second best, and so on, among the different interventions under evaluation. A larger SUCRA score indicates a more effective intervention. Abbreviation: BP, bisphosphonate; HRT, hormone replacement therapy; mAb, monoclonal antibody; PTH, parathyroid hormone; SERM, selective estrogen receptor modulator; SrRan, strontium ranelate; Vit D, vitamin D; Vit K, vitamin K.

**Figure 5:**
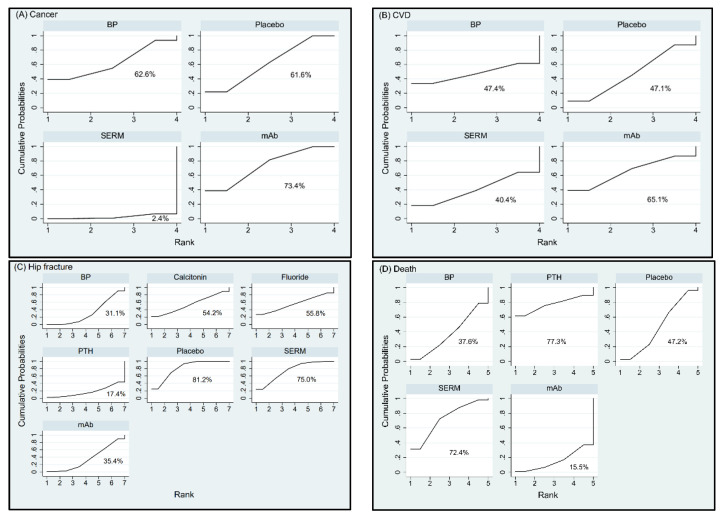
Cumulative rankograms: plots of the surface under the cumulative ranking curves (SUCRAs) for the incidence of (**A**) cancer, (**B**) cardiovascular disease (CVD), (**C**) hip fracture, and (**D**) death with various treatments in the postmenopausal osteoporosis networks. A larger SUCRA score indicates a higher risk for the event to occur with the intervention. Abbreviation: BP: bisphosphonate; mAb: monoclonal antibody; PTH: parathyroid hormone; SERM: selective estrogen receptor modulator.

The rankings of treatment effectiveness regarding BMD percentage increase or drug safety concerning incidence of adverse events are presented in Figure 4 and Figure 5, respectively, and Supplementary Figure S4 based on the SUCRA values. In terms of effectiveness in increasing BMD at LS, fluoride (94.9%) was ranked as the best intervention, followed in order by PTH, mAb, SrRan, BP, HRT, SERM, calcitonin, and VitD; while placebo (6.5%) was the least effective intervention. In terms of effectiveness in increasing BMD at TH, SrRan (95.6%) was ranked as the best, followed in order by mAb, HRT, BP, PTH, SERM, Fluoride, VitD, and calcitonin; while placebo (3.5%) was the least effective intervention.

**Figure 6:**
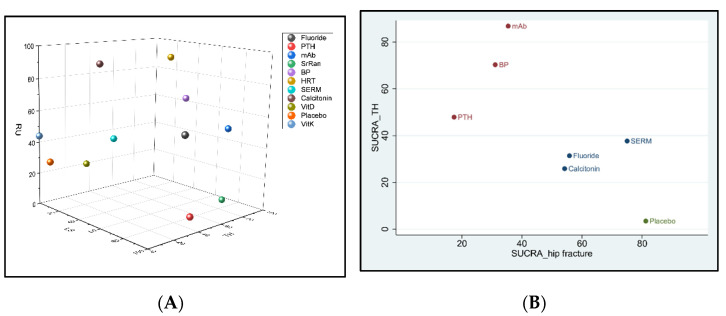
Clustered ranking plot of the postmenopausal osteoporosis network based on the surface under the cumulative ranking curve (SUCRA) values for (**A**) the increase of bone mineral density (BMD) percentage simultaneously at the lumbar spine (LS), total hip (TH), and distal radius (RU). Treatments lying in the upper right corner are more effective and acceptable than the other treatments. Clustered ranking plot based on SUCRA values for (**B**) the increase of BMD percentage at TH versus incidence of new hip fractures. Each color represents a group of treatments that belong to the same cluster. Interventions lying in the upper left corner were more favorable than the other interventions. Here, the red dots indicate the more effective interventions that increase BMD at TH but also reduce incidence of new hip fracture. Abbreviation: BP, bisphosphonate; HRT, hormone replacement therapy; mAb, monoclonal antibody; PTH, parathyroid hormone; SERM, selective estrogen receptor modulator; SrRan, strontium ranelate; Vit D, vitamin D; Vit K, vitamin K.

In terms of effectiveness in increasing BMD at RU, HRT (93%) was ranked as the best intervention, followed in order by calcitonin, BP, fluoride, mAb, VitK, SERM, placebo, and VitD; while PTH (3.3%) was the least effective intervention. In terms of risk for cancer, mAb (73.4%) appeared to have the highest probability for cancer to occur followed by BP and placebo; while SERM (2.4%) was associated with the lowest incidence of cancer. In terms of risk for CVD, mAb (65.1%) was ranked to have the highest risk for CVD followed by BP and placebo; while SERM (40.4%) revealed the lowest incidence of CVD. As for the complication of death, PTH (77.3%) showed the highest incidence of death followed by SERMs, placebo, and BP; while mAb (15.5%) indicated least risk for death. With respect to incidence of new hip fracture, placebo ranked first (81.2%), indicating that all other interventions reduced the risk of hip fractures compared to those treated by placebo. According to SUCRA, SERMs (75%) showed higher incidence of hip fracture followed by fluoride, calcitonin, mAb, and BP; while PTH (17.4%) appeared to be the most efficacious drug in preventing new hip fractures. The treatment hierarchy considering simultaneously the treatment efficacies of increase in BMD percentage at the three different anatomic sites was summarized and presented in Figure 6A using clustered ranking plot. Interventions lying in the upper right corner were more effective than the other interventions. When taking into account all anatomic sites for the effectiveness of osteoporosis treatment, BP, mAb, and fluoride seem to have a balanced efficacy in increasing BMD at all sites (Figure 6A). The treatment hierarchy considering simultaneously the treatment efficacies of increasing BMD at total hip and decreasing the risk of hip fracture was summarized in Figure 6B. Interventions lying in the upper left corner were more favorable than the other interventions. Considering both the effectiveness of increasing BMD at total hip and preventing hip fracture, mAb, BP, and PTH are more favorable among all interventions (Figure 6B).

### 3.4. Evaluating the Inconsistency between Direct and Indirect Evidence

According to the tests of inconsistency with the design-by-treatment and loop-inconsistency models (Supplementary Figure S5), all *p* values were higher than 0.05, suggesting no significant inconsistency in terms of drug efficacy or safety. However, side-splitting models showed great inconsistency in the comparison between the effectiveness of BMD increase for PTH and BP at lumbar spine and total hip, with corresponding *p* values of 0.002 and 0.007, respectively. At lumbar spine, the indirect evidence had less extent of PTH in increasing BMD percentage over BP. At total hip, the indirect evidence favored BP, while the direct evidence favored PTH in increasing BMD percentage. The comparison-adjusted funnel plots (Supplementary Figure S6) and Egger’s test (Supplementary Figure S7) showed no publication bias in most measurements, except for the outcomes of BMD percentage change at lumbar spine (*p* = 0.021) and total hip (*p* = 0.013). The between-trial heterogeneity and the small-study effect are the most likely explanations for the bias.

## 4. Discussion

Serious osteoporotic fractures and hip fractures lead to impaired mobility and become a cause a high degree of mortality. Therefore, it is important for clinicians to know the comparative efficacy of various interventions as well as safety. Since there is no trial comparing all relevant interventions, we conducted this network meta-analysis to distinguish treatments more favorable than others with respect to efficacy and safety. In this study, we included trials with participants objectively diagnosed as postmenopausal osteoporosis by using DXA. Those that used other tools for diagnosis or detection of fractures were excluded.

There are three main categories of pharmaceutical interventions for postmenopausal osteoporosis. One is anti-resorptive agents containing hormone replacement therapy (HRT; such as estrogen, estrogen-progestin), selective estrogen receptor modulators (SERMs; such as bazedoxifen, raloxifene), calcitonin, bisphosphonates (BP; alendronate, ibandronate, risedronate, zoledronate), and monoclonal antibody (mAb; such as denosumab, a RANKL inhibitor or romosozumab, a mAb against sclerostin). The other category is drugs with anabolic effects on bone such as strontium ranelate (SrRan) and parathyroid hormone (PTH; PTH1-84, PTH1-34, teriparatide, abaloparatide). Other options include supplemental calcium, sodium fluoride, vitamin D (VitD), and vitamin K (VitK). BP is the traditional first-line drug for treatment of postmenopausal osteoporosis. Recent meta-analyses show that denosumab improves BMD significantly more than BP at the lumbar spine, total hip, and femoral neck [16,36], but has no benefit for reducing risk of fracture than BP [36]. With the number of therapeutic agents increasing, most studies only focus on the treatment effects of specific types of medicines and lack the comprehensive comparison. As fragility fracture is one of the most disabling consequences of aging in women, many studies also report efficacy in prevention of fracture in addition to BMD change [37]. However, long-term of use of certain types of medicine may be associated with serious complications. For example, in relatively healthy postmenopausal women, HRT may increase the risk of coronary event, venous thromboembolism, stroke, breast cancer, gallbladder disease, and death from lung cancer [38].

Although these studies seem to compare many different interventions, several medicines belong to the same categories if grouped according to the pharmaceutical mechanisms. There are in fact fewer types of interventions compared than claimed. In the meantime, these network meta-analyses just pooled in the number of all adverse events; none of them specified the types of adverse events or serious adverse events evaluated. Our present network meta-analysis, including 94 RCTs, reports the most comprehensive review to date on the efficacy and safety of 11 different types of interventions grouped according to pharmaceutical mechanisms. We are also the first to compare incidence of different types of adverse events regarding the use of different interventions. We have found that different interventions have their anatomical preference with respect to efficacy. As mentioned above, fluoride is the most effective in increasing lumbar spine BMD, SrRan is the most effective in increasing total hip BMD, while HRT is the most effective in increasing radius BMD. Possible explanation of this site-specific manner is still pending, but individualized/personalized medication could be considered according to the physical conditions of the specific patient. When taking into account the effectiveness of BMD percentage increase at all three anatomic sites, BP, mAb, and fluoride seem to have a balanced efficacy in increasing BMD at all sites. However, efficacy in increasing BMD does not necessarily mean a greater effect in preventing fracture. Results from clustered ranking plot show that considering both the effectiveness of increasing BMD at total hip and preventing hip fracture, mAb, BP, and PTH are more favorable among all interventions.

Although fluoride ranks best in increasing BMD at lumbar spine, the evidence is based on trials conducted around year 2000 or earlier. Fluoride has been used as a potential therapy for postmenopausal osteoporosis decades ago, but it is not frequently used as a first line drug nowadays. One possible reason is that the effects of fluoride to increase BMD do not result in reduction of fractures. According to the meta-analysis of Haguenauer et al., as the dose of fluoride increases, the risks of non-vertebral fracture and gastrointestinal side effects increase without any effect on the vertebral fracture rate [39]. Our results also show that fluoride is a less favorable treatment option considering simultaneously the efficacy of increase in BMD at total hip and reduction of hip fracture (Figure 6B).

Postmenopausal osteoporosis is a consequence of long-term postmenopausal estrogen deficiency leading to progressive bone loss. Therefore, hormone replacement therapy (HRT) has been used to prevent osteoporosis in postmenopausal women. Our results show that HRT ranks first and third in increasing BMD at radius and total hip, respectively among all interventions (Figure 4). However, as it increases risks of CVD, cancers, or death in generally fit postmenopausal women, recent pairwise meta-analysis suggests using HRT to prevent osteoporosis only for whom at significant osteoporosis risk and when non-estrogen therapies are unsuitable [38]. Due to paucity of trials, HRT was not compared in our network meta-analysis with other interventions for risks of adverse events. SERMs are a class of drugs that act on the estrogen receptor to selectively inhibit or stimulate estrogen-like action in different tissues. Raloxifene is the first clinically available SERM to prevent both osteoporosis and breast cancer. Our results (Table 1) show that although statistically insignificant, SERM is considerably less potent than HRT in increasing BMD at all sites. SERM ranks best in reducing risk of cancer and CVD; however, it causes high incidence of death (PTH (77.3%) > SERM (72.4%) > placebo (47.2%) > BP (37.6%) > mAb (15.5%)) among the available interventions evaluated.

Bisphosphonate (BP), a synthetic pyrophosphate analog, is the most common treatment for osteoporosis. Our study has supported that BP is satisfactory in increasing BMD and reducing the risk of hip fracture. Although oral BP has proven efficacy and generally good tolerability, it is associated with upper gastrointestinal (GI) side effects [19] and infrequent side effects such as pyrexia, renal function impairment, hypocalcemia, and avascular osteonecrosis of the jaw (ONJ) [19,40]. Until now, the most efficacious management approach for ONJ has not been clearly established [40]. Medication-related ONJ has been reported to be associated with high cumulative doses of bisphosphonates or denosumab [41]. Since patients with long-term high-dose antiresorptive drugs intake may be considered as high-risk patients for ONJ, we attempted to compare the incidence of ONJ among all osteoporosis medications. However, of the 98 included studies in systematic review, only five trials intended to investigate the complication of ONJ (Table 3A). Due to insufficiency of data, meta-analysis could not be performed. For those who have received invasive oral procedures, the incidence of ONJ is higher (0.68%) compared to those who have not (0.05%). Overall, the incidence rate of ONJ is rare, which is also probably due to the lack of long-term data in RCTs [35].

Denosumab works by preventing the development of osteoclasts to protect bone from degradation, thereby counters the progression of osteoporosis. The largest study to date investigating denosumab was the FREEDOM trial [33]. After 36 months denosumab treatment, the results showed no significant increased risk of cancer, CVD, infection, delayed fracture healing, hypocalcemia, or death, and no cases of ONJ. However, a 7-year extension of the FREEDOM trial with denosumab has detected additional 13 cases of ONJ in total [35]. Romosozumab, a monoclonal antibody to inhibit sclerostin, has a dual effect of increasing bone formation and decreasing bone resorption. After the one-year romosozumab treatment, some hypersensitivity reactions, one ONJ with ill-fitted dentures, and an atypical femoral fracture with prodromal pain at fracture site were reported [34]. Results from our network meta-analysis show that both BP and mAb have a balanced efficacy in increasing BMD at all sites and lower risk of new hip fracture among all interventions. Although statistically insignificant, mAb also shows least risk of death (15.5%) but higher risk of CVD (65.1%) and cancer (73.4%).

PTH, teriparatide, induces differentiation of pre-osteoblasts into osteoblasts, stimulates osteoblasts to form new bone, decreases osteoblast apoptosis, and directly promotes bone formation with an increase in the rates of both bone remodeling and modeling; it restores the structure of trabecular bone and increases cortical thickness and diameter in osteoporosis [42]. Results from our network meta-analysis show that PTH ranks first in reducing risk of hip fracture and second in increasing BMD at lumbar spine. However, it ranks worst in increasing BMD at radius and moderate at total hip. Although statistically insignificant, PTH is associated with highest risk of death (77.3%).

Strontium ranelate (SrRan) has a dual action to both increase new bone formation by osteoblasts and decrease bone resorption by osteoclasts. Our meta-analysis shows that SrRan ranks first in increasing BMD at total hip and favorable at lumbar spine. In March 2014, because of its CVD risks, the European Medicines Agency recommended to restrict SrRan only to the treatment of severe osteoporosis in postmenopausal women and adult men at high risk of fracture who cannot use other osteoporosis treatments due to contraindications or intolerance (https://www.gov.uk/drug-safety-update/strontium-ranelate-cardiovascular-risk, accessed on 15 January 2021). Due to lack of data, the efficacy of SrRan at radius BMD and preventing hip fracture or safety regarding CVD, cancer, or death may not be evaluated in our population of postmenopausal osteoporotic women. Collectively, after weighing treatment efficacy against risk of complications, the results of our network meta-analysis show that BP and mAb are the more favorable interventions to comprehensively increase BMD at all sites and reduce the risks of hip fracture and death. The safety of the other interventions needs further evaluation.

To our knowledge, this present study presents the most comprehensive network meta-analysis to date with respect to the effects of therapies on BMD at different anatomic sites and incidence of risks for cancer, CVD, hip fracture, and death in postmenopausal women with osteoporosis. However, some limitations should be acknowledged. First of all, some inconsistency existed in the comparison between the effectiveness of BMD increase with PTH and BP at lumbar spine and total hip. The direct evidence showed greater extent of PTH to increase BMD over BP than the indirect evidence. This may be due to the fact that only four trials directly compared BMD at total hip or lumbar spine between BP (*n* = 340) and PTH (*n* = 333). Among the four trials, one with relatively small sample size reported substantially greater WMD than others to favor PTH (*n* = 20) over BP (*n* = 29) [43]. Secondly, modes of drug administration in the included studies differ in the prescribed dosage, potency, or duration of different medications. We have only grossly grouped the interventions according to their pharmacologic mechanisms for practical reason. However, each individual group may consist of different drugs (for example, denosumab and romosozumab have been grouped into the same category as mAb). This may contribute to the increased heterogeneity among studies. Thirdly, since there are only a small number of relevant studies available in the analysis of specific types of adverse events, some comparisons were missing in the original primary studies for cancer, CVD, and death. The results have to be interpreted with caution. As different interventions may be associated with risks for different cancers or reasons of death, we have listed the associated types of cancers or reasons of death in Table 3B,C. Fourthly, recent study has shown superior performance of combination therapy over monotherapy to improve BMD at lumbar spine and total hip without risk of serious adverse events. Combination therapy has also shown an advantage over monotherapy to reduce fracture risk [44,45,46,47]. However, our network meta-analysis only compared monotherapies. Fifthly, the follow-up period of included trials for our meta-analysis ranged from six months to eight years. As treatment of osteoporosis requires long-term therapy to gain continuing benefit, the risk of adverse events in this study may be underestimated for the relatively short-term trials. Lastly, we only assessed risk of hip fracture in our study. Since increase of BMD may not necessarily result in reduction of fractures, future research is necessary to evaluate the efficacy of these interventions to prevent other types/sites of fractures. Further network meta-analysis is also necessary to evaluate the best combination of therapies as well as to assess the tolerability and cost-effectiveness of the different interventions.

## 5. Conclusions

Our results have shown that different interventions have their anatomical preference with respect to efficacy. Fluoride is the most effective in increasing lumbar spine BMD, SrRan is the most effective in total hip BMD, while HRT is the most effective in the radius BMD. PTH is the most effective to prevent new hip fracture. As for adverse events, SERM exhibits least risk for cancer and CVD; while mAb exhibits least risk for death. BP, mAb, and fluoride seem to have a balanced efficacy in increasing BMD at all sites. Considering simultaneously the effectiveness of increasing BMD at total hip and preventing hip fracture, mAb, BP, and PTH are more favorable among all interventions. After weighing treatment efficacy against risk of complications, BP and mAb are the more favorable interventions to comprehensively increase BMD at all sites and reduce the risks of hip fracture and death. The safety of the other interventions needs further evaluation. Individualized/personalized medication could be considered according to the physical conditions of the specific patient in future.

## Figures and Tables

**Table 1:** Result of network meta-analysis and pairwise meta-analysis for bone mineral density (BMD) percentage change at lumbar spine (**A**), total hip (**B**), and distal radius (**C**).

**A. Lumbar Spine**									
VitD								−6.17 (−10.10, −2.24)	
−9.83 (−14.87, −4.80)	Fluoride							4.52 (0.89, 8.15)	8.98 (7.01, 10.95)
−7.28 (−12.18, −2.37)	2.55 (−0.86, 5.97)	SrRan						0.90 (−1.53, 3.33)	6.88 (−0.14, 13.89)
−2.28 (−6.85, 2.29)	7.55 (4.62, 10.49)	5.00 (2.30, 7.69)	Calcitonin	−4.49 (−5.81, −3.16)				−1.90 (−2.75, −1.05)	1.17 (0.46, 1.87)
−8.71 (−13.23, −4.18)	1.13 (−1.74, 4.00)	−1.43 (−4.05, 1.20)	−6.43 (−8.21, −4.64)	PTH			1.20 (−0.39, 2.79)	5.42 (1.02, 9.83)	7.45 (6.59, 8.31)
−2.31 (−6.99, 2.36)	7.52 (4.41, 10.62)	4.96 (2.08, 7.84)	−0.03 (−2.32, 2.25)	6.39 (4.19, 8.59)	SERM			−3.86 (−6.87, −0.84)	1.81 (1.04, 2.58)
−4.27 (−9.24, 0.71)	5.56 (2.01, 9.11)	3.01 (−0.34, 6.36)	−1.99 (−4.84, 0.86)	4.44 (1.65, 7.23)	−1.96 (−4.98, 1.07)	HRT		−4.60 (−6.17, −3.02)	4.77 (2.97, 6.57)
−8.14 (−13.24, −3.04)	1.69 (−1.99, 5.38)	−0.86 (−4.35, 2.63)	−5.86 (−8.84, −2.88)	0.57 (−2.13, 3.26)	−5.82 (−9.01, −2.63)	−3.87 (−7.49, −0.25)	mAb		8.25 (−1.65, 18.15)
−6.20 (−10.49, −1.91)	3.63 (0.10, 6.27)	1.08 (−1.29, 3.45)	−3.92 (−5.48, −2.36)	2.51 (1.07, 3.95)	−3.88 (−5.74, −2.03)	−1.93 (−4.44, 0.58)	1.94 (−0.81, 4.69)	BP	6.06 (5.69, 6.44)
−0.38 (−4.75, 3.99)	9.45 (6.89, 12.01)	6.90 (4.62, 9.17)	1.90 (0.43, 3.36)	8.32 (6.99, 9.66)	1.93 (0.15, 3.72)	3.89 (1.41, 6.37)	7.76 (5.10, 10.41)	5.82 (5.00, 6.63)	Placebo
**B. Total Hip**									
VitD								−2.20 (−4.20, −0.20)	
−0.40 (−4.78, 4.00)	Fluoride							−2.03 (−8.01, 3.94)	1.57 (0.92, 2.21)
−4.39 (−8.91, 0.12)	−4.00 (−6.33, −1.67)	SrRan						1.40 (−0.47, 3.27)	5.76 (0.87, 10.66)
−0.17 (−4.46, 4.12)	0.22 (−1.66, 2.11)	4.22 (2.05, 6.39)	Calcitonin	0.10 (−0.24, 0.44)				−1.10 (−1.82, −0.38)	1.24 (0.71, 1.77)
−1.16 (−5.41, 3.08)	−0.77 (−2.53, 0.99)	3.23 (1.17, 5.28)	−0.99 (−2.45, 0.47)	PTH			−1.20 (−1.80, −0.60)	1.23 (−0.96, 3.43)	1.59 (0.97, 2.21)
−0.70 (−5.17, 3.76)	−0.31 (−2.57, 1.95)	3.69 (1.19, 6.19)	−0.53 (−2.62, 1.56)	0.46 (−1.51, 2.44)	SERM			−1.00 (−1.72, −0.28)	1.73 (1.45, 2.01)
−2.42 (−6.98, 2.14)	−2.03 (−4.47, 0.42)	1.97 (−0.70, 4.64)	−2.25 (−4.53, 0.04)	−1.26 (−3.44, 0.93)	−1.72 (−4.32, 0.88)	HRT		0.19 (−1.03, 1.41)	3.92 (2.46, 5.38)
−3.50 (−8.15, 1.16)	−3.10 (−5.70, −0.50)	0.90 (−1.91, 3.70)	−3.32 (−5.74, −0.90)	−2.33 (−4.41, −0.25)	−2.79 (−5.54, −0.04)	−1.07 (−3.98, 1.83)	mAb		6.80 (6.56, 7.04)
−2.20 (−6.30, 1.90)	−1.80 (−3.35, −0.26)	2.19 (0.31, 4.07)	−2.03 (−3.28, −0.78)	−1.04 (−2.10, 0.03)	−1.50 (−3.26, 0.26)	0.22 (−1.76, 2.20)	1.29 (−0.91, 3.50)	BP	3.72 (2.83, 4.61)
1.26 (−2.90, 5.41)	1.65 (0.18, 3.12)	5.65 (3.83, 7.46)	1.43 (0.22, 2.64)	2.42 (1.43, 3.41)	1.96 (0.22, 3.70)	3.68 (1.70, 5.65)	4.75 (2.61, 6.90)	3.46 (2.83, 4.08)	Placebo
**C. Distal Radius**									
VitK	1.70 (1.05, 2.35)		−3.50 (−4.16, −2.84)			−3.90 (−4.63, −3.17)		−1.49 (−2.14, −0.84)	1.29 (0.67, 1.92)
1.47 (−1.83, 4.76)	VitD		−5.20 (−5.72, −4.68)			−5.60 (−6.22, −4.98)		−3.10 (−3.67, −2.53)	−0.30 (−0.84, 0.24)
−0.91 (−4.58, 2.75)	−2.38 (−6.50, 1.74)	Fluoride							2.01 (−2.46, 6.47)
−3.73 (−7.03, −0.44)	−5.20 (−8.74, −1.66)	−2.82 (−6.94, 1.30)	Calcitonin			−0.40 (−1.02, 0.22)		2.10 (1.53, 2.67)	4.90 (4.36, 5.44)
3.31 (0.08, 6.53)	1.84 (−1.90, 5.57)	4.22 (0.68, 7.76)	7.04 (3.30, 10.77)	PTH			−3.62 (−4.75, −2.48)	−7.80 (−9.70, −5.90)	−1.03 (−5.89, 3.83)
0.21 (−4.16, 4.57)	−1.26 (−6.01, 3.49)	1.12 (−3.42, 5.66)	3.94 (−0.82, 8.69)	−3.10 (−7.36, 1.16)	SERM				0.70 (−0.13, 1.53)
−4.13 (−7.45, −0.81)	−5.60 (−9.16, −2.04)	−3.21 (−7.35, 0.92)	−0.40 (−3.96, 3.16)	−7.44 (−11.19, −3.68)	−4.34 (−9.11, 0.43)	HRT		2.50 (1.84, 3.16)	5.30 (4.66, 5.94)
−0.34 (−4.61, 3.94)	−1.81 (−6.48, 2.87)	0.58 (−3.94, 5.09)	3.39 (−1.28, 8.07)	−3.64 (−6.45, −0.84)	−0.54 (−5.65, 4.56)	3.79 (−0.89, 8.48)	mAb		
−1.59 (−4.04, 0.86)	−3.06 (−6.15, 0.02)	−0.68 (−3.77, 2.41)	2.14 (−0.95, 5.22)	−4.90 (−7.34, −2.46)	−1.80 (−5.70, 2.10)	2.54 (−0.57, 5.65)	−1.26 (−4.97, 2.46)	BP	2.22 (1.72, 2.72)
0.91 (−1.53, 3.34)	−0.56 (−3.64, 2.52)	1.82 (−0.91, 4.55)	4.64 (1.56, 7.72)	−2.40 (−4.65, −0.15)	0.70 (−2.92, 4.32)	5.04 (1.93, 8.14)	1.24 (−2.35, 4.84)	2.50 (1.06, 3.94)	Placebo

Results of network meta-analysis (mixed comparison; lower-left portion) and pairwise meta-analysis (direct comparison; upper-right portion) for percentage change in bone mineral density (BMD) from baseline at distal radius (RU) in postmenopausal women with osteoporosis. Estimates are presented as weighted mean difference (WMD) and 95% confidence interval (CI; in parentheses). Comparisons between treatments should be read from left to right. The estimate of treatment effectiveness is located at the intersection of the column-defining treatment and the row-defining treatment. A WMD above 0 favors the column-defining treatment of the mixed comparison. Abbreviation: BP, bisphosphonate; HRT, hormone replacement therapy; mAb, monoclonal antibody; PTH, parathyroid hormone; SERM, selective estrogen receptor modulator; VitD, vitamin D; VitK, vitamin K.

**Table 2:** Result of network meta-analysis and pairwise meta-analysis for the incidence of cancer (**A**), cardiovascular disease (**B**), hip fracture (**C**), and death (**D**).

**A. Cancer**						
SERM						0.65 (0.40, 0.90)
0.62 (0.43, 0.91)		mAb		1.09 (0.58, 1.61)		1.03 (0.76, 1.29)
0.64 (0.37, 1.10)		1.03 (0.66, 1.59)		BP		1.12 (0.45, 1.79)
0.65 (0.48, 0.88)		1.04 (0.83, 1.31)		1.01 (0.65, 1.58)		Placebo
**B. Cardiovascular Disease**						
SERM						1.03 (0.79, 1.26)
0.92 (0.63, 1.35)		mAb				1.05 (0.86, 1.24)
0.97 (0.56, 1.68)		1.06 (0.62, 1.79)		BP		1.00 (0.56, 1.43)
0.97 (0.73, 1.29)		1.05 (0.82, 1.35)		1.00 (0.63, 1.59)		Placebo
**C. Hip Fracture**						
Fluoride						1.04 (−0.34, 2.41)
0.99 (0.20, 4.99)	Calcitonin					0.75 (−0.25, 1.75)
1.86 (0.36, 9.63)	1.87 (0.44, 7.98)	PTH			0.40 (−1.25, 2.04)	0.75 (−0.75, 2.25)
0.78 (0.20, 3.01)	0.79 (0.26, 2.37)	0.42 (0.13, 1.33)	SERM			0.95 (0.50, 1.40)
1.24 (0.32, 4.82)	1.25 (0.41, 3.80)	0.67 (0.21, 2.13)	1.58 (0.81, 3.08)	mAb		0.60 (0.11, 1.09)
1.28 (0.34, 4.74)	1.29 (0.45, 3.70)	0.69 (0.24, 1.99)	1.63 (0.93, 2.86)	1.03 (0.57, 1.87)	BP	0.58 (0.23, 0.92)
0.75 (0.21, 2.65)	0.75 (0.28, 2.05)	0.40 (0.14, 1.15)	0.95 (0.61, 1.50)	0.60 (0.37, 0.98)	0.58 (0.42, 0.82)	Placebo
**D. Death**						
PTH					2.17 (1.27, 3.07)	0.60 (−0.83, 2.03)
1.18 (0.46, 3.05)		SERM				1.23 (0.79, 1.67)
1.75 (0.65, 4.71)		1.49 (0.86, 2.58)	mAb			0.78 (0.50, 1.05)
1.49 (0.64, 3.45)		1.26 (0.74, 2.14)	0.85 (0.47, 1.53)		BP	1.02 (0.65, 1.38)
1.40 (0.58, 3.39)		1.19 (0.83, 1.69)	0.80 (0.52, 1.21)		0.94 (0.63, 1.40)	Placebo

Results of network meta-analysis (mixed comparison; lower-left portion) and pairwise meta-analysis (direct comparison; upper-right portion) for the incidence of death in postmenopausal women with osteoporosis. The outcomes are expressed as odds ratio (OR) (95% confidence intervals). For the pairwise meta-analyses, OR of less than 1 indicate that the treatment specified in the row had fewer incidences than that specified in the column. For the network meta-analysis (NMA), OR of less than 1 indicate that the treatment specified in the column had fewer incidences than that specified in the row. Abbreviation: BP, bisphosphonate; mAb, monoclonal antibody; PTH, parathyroid hormone; SERM, selective estrogen receptor modulator.

**Table 3:** Previous reports for medication-related osteonecrosis of the jaw (ONJ), cancer, and death. (**A**) Medication-Related Osteonecrosis of the Jaw (ONJ), (**B**) Cancer, (**C**) Death.

A. Medication-Related Osteonecrosis of the Jaw (ONJ)			
Study	Treatment	Total Number (Incidence)	Follow Up
Watts, N. B. 2019 (7-Year FREEDOM extension)	Denosumab (3 + 7 yrs) ^a^ Placebo (3 yrs)/Denosumab (7 yrs)	2343 *(7)* ^b^ 2207 *(6)*	10 years 10 years
Kendler, D.L. 2018	Teriparatide Risedronate (BP)	680 *(0)* 680 *(0)*	24 months 24 months
Saag, K.G. 2017	Romosozumab (mAb) Alendronate (BP)	2040 *(0)* 2014 *(0)*	1 year 1 year
Cosman, F. 2016	Romosozumab (mAb) Placebo Romosozumab to Denosumab ^c^ Placebo to Denosumab	3581 *(1)* 3576 *(0)* 3581 *(2)* 3576 *(1)*	12 months 12 months 24 months 24 months
Cummings, S.R. 2009 (FREEDOM study)	Denosumab (mAb) Placebo	3902 *(0)* 3906 *(0)*	36 months 36 months
**B. Cancer**			
**Study**	**Intervention**	**Type of Cancers**	**Number of Events (Percentage of Events, %)**
(Saag, K. G. et al. 2017)	SC 210mg Romosozumab QM for 12 M Oral 70 mg Alendronate QW for 12 M	All cancers	Romosozumab group: 31 (1.5) Alendronate group: 28 (1.4)
(Cosman, F. et al. 2016)	SC 210 mg Romosozumab QM for 12 M Placebo	All cancers	Romosozumab group: 59 (1.6) Placebo group: 69 (1.9)
(Palacios, S. et al. 2015)	Oral 20 mg Bazedoxifene daily for 7 yrs Placebo	Breast cancer	Bazedoxifene group: 23 (0.6) Placebo group: 11 (0.6)
(LaCroix, A. Z. et al. 2010)	Oral 0.5 mg Lasofoxifene daily for 5 yrs Placebo	Breast cancer	Lasofoxifene group: 5 (0.41) Placebo group: 24 (1.97)
(Christiansen, C. et al. 2010)	Oral 20 mg Bazedoxifene daily for 3 yrs Oral 60 mg Raloxifene daily for 3 yrs Placebo	Breast cancer	Bazedoxifene group: 6 (0.3) Raloxifene group: 7 (0.4) Placebo group: 8 (0.4)
(Cummings, S. R. et al. 2009)	SC 60 mg Denosumab Q6 M for 36 M Placebo	All cancers	Denosumab group: 187 (4.8) Placebo group: 166 (4.3)
(Martino, S. et al. 2005)	Oral 60 mg Raloxifene daily for 8 yrs Placebo	All cancers (excluding non-melanoma skin cancer)	Raloxifene group: 156 (5.7) Placebo group: 110 (8.6)
(Barrett-Connor, E. et al. 2004)	Oral 60 or 120 mg Raloxifene daily for 4 yrs Placebo	Invasive breast cancer, endometrial cancer	Invasive breast cancer Raloxifene group: 17 (0.1) Placebo group: 35 (0.4) Endometrial cancer Raloxifene group: 7 (0.04) Placebo group: 5 (0.06)
(Martino, S. et al. 2004)	Oral 60 mg Raloxifene daily for 8 yrs Placebo	Breast cancers	Raloxifene group: 31 (0.9) Placebo group: 30 (1.8)
(Reginster, J. Y. et al. 2000)	Oral 5 mg Risedronate daily for 1 yr Placebo	All cancers	Risedronate group: 19 (4.7) Placebo group: 17 (4.2)
**C. Death**			
**Study**	**Intervention**	**Reasons of Death**	**Number of Events (Percentage of Events, %)**
(Kendler, D. L. et al. 2018)	SC 20 ug Teriparatide daily for 24 M Oral 35 mg Risedronate weekly for 24 M	N/A ^(1)^	Teriparatide group: 15 *(2.2)* Risedronate group: 7 *(1.0)*
(Koh, J. M. et al. 2016)	SC 60 mg Denosumab Q6 M for 6 M Placebo	Traumatic subdural hemorrhage due to motorcycle accident	Denosumab group: 1 *(1)* Placebo group: 0 *(0)*
(Cosman, F. et al. 2016)	SC 210 mg Romosozumab QM for 12 M Placebo	Romosozumab: 17 from Cardiovascular causes Placebo: 15 from Cardiovascular causes	Romosozumab group: 23 *(0.6)* Placebo group: 29 *(0.8)*
(Miller, P. D. et al. 2016)	SC 80 ug Abaloparatide daily for 1 yr SC 20 ug Teriparatide daily for 1 yr Placebo	Abaloparatide: sepsis, bronchiectasis, ischemic heart disease Teriparatide: pancreatic cancer, general health deterioration, cardiorespiratory arrest Placebo: bowel cancer, intestinal obstruction, myocardial infarction, dissecting aneurysm of the aorta, sudden death	Abaloparatide group: 3 *(0.4)* Teriparatide group: 3 *(0.4)* Placebo group: 5 *(0.6)*
(Palacios, S. et al. 2015)	Oral 20 mg Bazedoxifene daily for 7 yrs Placebo	N/A ^(2)^	Bazedoxifene group: 52 *(1.4)* Placebo group: 18 *(1.0)*
(Cummings, S. R. et al. 2010)	Oral 0.25 mg Lasofoxifene daily for 5 yrs Placebo	Lasofoxifene: 34 from cancers Placebo: 20 from cancers	Lasofoxifene group: 90 *(3.2)* Placebo group: 65 *(2.3)*
(Cummings, S. R. et al. 2009)	SC 60 mg Denosumab Q6 M for 36 M Placebo	N/A	Denosumab group: 70 *(1.8)* Placebo group: 90 *(2.3)*
(Black, D. M. et al. 2007)	IV (15 min) 5 mg Zoldronic acid yearly for 3 yrs Placebo	Zoldronic acid: 20 from stroke; 39 from cardiovascular causes Placebo: 11 from stroke; 33 from cardiovascular causes	Zoldronic acid group: 130 *(3.4)* Placebo group: 112 *(2.9)*
(Martino, S. et al. 2005)	Oral 60 mg Raloxifene daily for 8 yrs Placebo	N/A	Raloxifene group: 47 *(1.7)* Placebo group: 29 *(2.3)*
(Boonen, S. et al. 2004)	Oral 5 mg Risedronate daily for 3 yrs Placebo	N/A	Risedronate group: 40 *(5.7)* Placebo group: 49 *(7.1)*

^a^ Ten-year treatment with denosumab in long-term group compared with three-year placebo and subsequently seven-year denosumab in crossover patients. ^b^ There were no reported cases of ONJ in the 3-year FREEDOM study. During the FREEDOM extension, there were 7 in long-term patients and 6 in crossover patients. ^c^ During open-label period, each patient was administrated denosumab subcutaneously every 6 months for additional 12 months. ^(1)^ The author considered that all deaths were unrelated to study drug. ^(2)^ The reasons of death were not defined in the 7-year dataset. However, previous 5-year follow-up study from de Villers, T. J. et al. 2011 summarized that 7 patients died from cardiovascular causes, 9 from oncology, 6 from others, 2 from unknown reason in Bazedoxifene group; while in placebo group, 6 died from cardiovascular causes, 5 from oncology, 1 from other, and 1 from unknown reason. Abbreviation: BP, bisphosphate; mAb, monoclonal antibody; Yr, year; SC: subcutaneously; QM: once a month; QW: once a week; Q6 M: every 6 months; yr: year; N/A, not available; SC, subcutaneously; QM, once a month; Q6 M, every 6 months; IV, intravenously.

## Data Availability

The datasets used and/or analyzed in the current study are available from the corresponding author on reasonable request.

## Supplementary Materials

The following are available online at https://www.mdpi.com/article/10.3390/jcm10143043/s1, Table S1: Search Strategies, Table S2: The Excluded Studies at Full-text Stage with Reasons of Exclusion, Table S3: Characteristics of the Included Studies, Table S4: Concerned Adverse Events Reported in Previous Studies, Figure S1: Risk of Bias Assessment Summary Graph (Review Authors’ Judgment as Low, Unclear, or High for Each Risk of Bias Item) Shown as Percentages Across All Included Studies, Figure S2: Forest Plots of Direct Pairwise Comparisons for BMD at (A) lumbar spine (LS) (B) total hip (TH) (C) radius (RU), Figure S3: Forest Plots of Direct Pairwise Comparisons for incidence of adverse events including (A) cancer (B) cardiovascular disease (CVD) (C) hip fracture and (D) death, Figure S4: The Surface Under the Cumulative Ranking Curves (SUCRAs) for the outcomes of individual interventions including BMD (A) at lumbar spine (LS), (B) total hip (TH), (C) radius (RU) and incidence of (D) cancer, (E) cardiovascular disease (CVD), (F) hip fracture, and (G) death, Figure S5: Assessment of Inconsistency Results Between Direct and Indirect Evidence for the Outcomes of BMD at (A) Lumbar Spine (LS) (B) Total Hip (TH) (C) Radius (RU) and Incidence of (D) Cancer (E) Hip fracture (F) Death within the Networks Using the Design-by-Treatment Interaction Models, Loop Inconsistency Models, and Side-splitting Models, Figure S6: Comparison-Adjusted Funnel Plots for the Outcomes of BMD at (A) lumbar spine (LS) (B) total hip (TH) (C) radius (RU) and incidence of (D) cancer (E) cardiovascular disease (CVD) (F) hip fracture (G) death, Figure S7: Egger’s Publication Bias Plots for the Outcomes of BMD at (A) lumbar spine (LS) (B) total hip (TH) (C) radius (RU) and the incidence of (D) cancer (E) cardiovascular disease (CVD) (F) hip fracture (G) death.
