# Supplementary material for: Efficacy and Safety of Postmenopausal Osteoporosis Treatments: A Systematic Review and Network Meta-Analysis of Randomized Controlled Trials
Source: J Clin Med. 2021 Jul 8;10(14):3043. doi: 10.3390/jcm10143043 (PMC8305263; doi:10.3390/jcm10143043)
Supplement: Supplementary file 1 [file jcm-10-03043-s001.zip › jcm-1247748-Supplementary.pdf]

Supplementary Materials Content

for **Efficacy and safety of postmenopausal osteoporosis treatments**

Table S1. Search Strategies……………………………………………………………………………….….2

Table S2. The Excluded Studies at Full-text Stage with Reasons of Exclusion…….…………………………......3

Table S3. Characteristics of the Included Studies……………………………………..……………………...5

Table S4. Concerned Adverse Events Reported in Previous Studies…………………………….………...….16

Figure S1: Risk of Bias Assessment Summary Graph…….……………………………….…..….…………..…….20

Figure S2: Forest Plots of Direct Pairwise Comparisons for BMD……………...……….……………….….…….22

Figure S3: Forest Plots of Direct Pairwise Comparisons for Adverse Events…..…………………….….…….….…..25

Figure S4: SUCRAs for the Outcomes of Individual Interventions…….…………………..………………..….29

Figure S5: Assessment of Inconsistency Results Between Direct and Indirect Evidence ...…………………...….31

Figure S6: Comparison-Adjusted Funnel Plots……………………...……………..…………………….…….43

Figure S7: Egger's Publication Bias Plots………………………..………………...……...……………...……..47

References……………………………..……………………………………………………………....….52

Table S1: Search Strategies

A	postmenopause osteoporosis OR post-menopause osteoporosis OR postmenopausal osteoporosis OR post-menopausal osteoporosis (***MeSHTerms***)
B	vitamin D OR calcium OR (bisphosphat* OR bis-phosphat* OR bisphosphonate* OR bis-phosphonate*) OR (Alendronate* OR alendronic acid* OR Fosamax OR Binosto OR Alendro* OR Marvil OR osmak) OR (Ibandronate* OR Ibandronic acid* OR Boniva OR Bondronat OR Bonviva) OR (Risedronate* OR Risedronic acid* OR Actonel OR Atelvia OR Benet OR Gusong OR Ribastamin OR Ribone OR Risofos) OR (Zoldronate* OR Zoldronic acid OR Zometa OR Reclast OR Aclasta) OR (Clodronate OR clodronic acid OR Bonefos) OR (Etidronate* OR etidronic acid* OR Didronel) OR (Pamidronate* OR pamidronic acid* OR Aredia) OR (Minodronate* OR minodronic acid*) OR (Tiludronate* OR tiludronic acid* OR Skelid) OR (Neridronate* OR neridronic acid*) OR (Olpadronate* OR olpadronic acid*) OR (Estrogen* OR Estradiol* OR norethisterone OR nor-ethisterone OR Medroxyprogesterone) OR (SERMs* OR Raloxifene OR Tamoxifen OR Toremifene OR Raloxifene OR Bazedoxifene) OR (parathyroid* OR PTH(1-34) OR Teriparatide OR PTH(1-84)) OR (Calcitonin OR Fortical) OR (RANK* OR monoantibod* or mono-antibod* or Denosumab) OR (Isoflavone OR Genistein) OR sodium fluoride OR (ranelic* OR Strontium ranelate) OR (steroid OR Tibilone) OR (diuretic* OR Hydro-chlorothiazide OR Hydrochlorothiazide) OR (statin* OR Atrovastatin OR Fluvastatin OR lovastatin OR Pravastatin OR Rosuvastatin) OR (nitrate* OR Nitric oxide) OR (beta-blocker* or betablocker*) ***(All fields)***
A **AND** B	Filters activated: **Clinical Trial**, Publication date to **2020/10/31**, **Humans**, **Female**.

Table S2: The Excluded Studies at Full-text Stage with Reasons of Exclusion

Study	Reasons
(Bonnick, S. et al. 2007[1], Chen, M. et al. 2001[2], Iseri, K. et al. 2018[3], Rubinacci, A. et al. 2003[4], Braga, de Castro Machado A. et al. 1999[5], Castelo-Branco, C. et al. 2000[6], Cooper, L. et al. 2003[7], Dawson-Hughes, B. et al. 1990[8] , Grados, F. et al. 2003[9], Haines, C. J. 1995[10], Harris, S. T. et al. 1991[11], Hillard, T. C. et al. 1994[12], Jirapinyo, M. et al. 2003[13])	Non-aging-related postmenopausal osteoporosis
(Fitzpatrick, L. A. et al. 2011[14], You, L. et al. 2010[15], Bhattoa, H. P. et al. 2004[16], Watts, N. B. et al. 2003[17], Bell, N. H. et al. 2002[18], Greenspan, S. L. et al. 2002[19], Greenspan, S. L. et al. 2003[20])	Patients with osteopenia included
(Grbic, J. T. et al. 2008[21])	Previous treatment for postmenopausal osteoporosis and continuous medication
(Chung, Ys. et al. 2009[22], Devogelaer, Jp. et al. 2007[23], Gallagher, J. C. et al. 2006[24], Cryer, B. et al. 2005[25], Iwamoto, J. et al. 2004[26], Iwamoto, J. et al. 2004[27], Keaveny, M. et al, 2007[28], Kung, A. W. et al, 2009[29], Ljunghall, S. et al, 1991[30], Lufkin, E. G. et al, 1994[31], Majima, T. et al. 2008[32], Gruber, H. E. et al. 1984[33], Miller, P. D. et al. 2000[34], Reid, I. R. et al. 2010[35], Riggs, B. L. et al. 1982[36], Stepan, J. J. et al. 2007[37], Van Der Poest Clement, E. et al. 2000[38])	BMD was not reported as percentage increase or no concerned adverse effects (death, cancer, CVD, hip fracture, ONJ)
(Engelke, K. et al. 2014[39], Yang, L. et al. 2013[40])	BMD was measured as volumetric BMD (vBMD)
(Arcoraci, V. et al. 2017[41], Agnusdei, D. et al. 1992[42], Gonnelli, S. et al. 1997[43], Moscarni, M. et al. 1994[44], Nakamura, T. et al. 2014[45], Saaf, M. et al. 1999[46], Zhang, X. et al. 2010[47], Zhao, G. et al. 2003[48], Eastell, R. et al. 2011[49], Eastell, R. et al. 2014[50], Cecchettin, M. et al. 1995[51], Chee, W. S. S. et al. 2003[52], Choi, Y. K. et al. 1997[53], Douglas, A. S. et al. 1995[54], Greendale, G. A. et al. 2002[55], Hampson, G. et al. 2003[56], Kovács, A. B. et al. 1994[57])	Non-pharmaceutical Interventions

(Hagino, H. et al. 2009[58], Iwamoto, J. et al. 2005[59], Kushida, K. et al. 2004[60], Li, M. et al. 2010[61], Miller, P. D. et al. 2008[62], Reid, D. M. et al. 2008[63], Sarioglu, M. et al. 2006[64], Tanko, L. B. et al. 2003[65], Balena, R. et al. 1998[66])	Comparison with drugs of the same class
(Suzuki, T. et al. 2018[67], Gurlek, A. et al, 1997[68], Lindsay, R. et al, 1997[69], Orimo, H. et al. 2011[70], Reid, I. R. et al. 2007[71], Ringe, J. D. et al. 2002[72], Deal, C. et al. 2005[73], Gutteridge, D. H. et al. 2002[74], Harris, S. T. et al. 2001[75], Hodsman, A. B. et al. 1997[76], Lindsay, R. et al. 1999[77])	Comparison with combination drug therapy
(Sakhaee, K. et al. 1993[78])	The BMD measurement was not by DXA
(Sone, T. et al. 2014[79])	Measurements of LS, TH and RU was not included in the reported sites
(Duvernoy, et al. 2005[80], Itabash, A. et al. 2015[81], Marcus, R. et al. 2003[82])	Post-hoc study of previously data
(Dempster, D. W. et al. 2018[83], Reginster, J. Y. et al. 2009[84], Reginster, J. Y. et al. 2012[85], Zanchetta, J. R. et al. 2010[86], Kanis, J. A. et al. 2005[87], Harrington, J. T. et al. 2004[88], Sorensen O. H. et al. 2003[89], Watts, N. B. et al. 2003[90], Goldstein, S. R. et al. 2002[91], Maricic, M. et al. 2002[92], Liao E. Y. et al. 2018[93])	Extension study and lack of related long-term BMD change or concerned adverse effects

Abbreviation: BMD, bone mineral density; CVD, cardiovascular diseases; ONJ, osteonecrosis of the jaw; DXA, dual-energy x-ray absorptiometry; LS, lumber spine; TH, total hip; RU, radius

Table S3: Characteristics of the Included Studies

Name	Country	Race (%)	Yrs since menopause	Group	Subject	Mean age	BMI
(Watts, N. B. et al. 2019)[94]	International	N/A	26.7±7.3	SC 60mg Denosumab Q6M for 7yrs	2343	74.9±5.0	N/A
			26.7±7.4	Placebo for 3yrs and Denosumab for 7 yrs	2207	74.8±5.1	
(Sugimoto, T. et al. 2019)[95]	Japan	Japanese (100)	N/A	IM 20U Elactonin weekly for 36M	316	75.5±5.7	23.3±3.4
				Placebo	309	75.5±5.7	23.2±3.3
(Kendler, D. L. et. Al. 2018)[96]	International	White (99)<br>Black (1)<br>Asian (1)	N/A	SC 20ug Teriparatide daily for 24M	498	72.6±8.77	26.9±4.61
				Oral 35mg Risedronate weekly for 24M	515	71.6±8.58	27.1±4.64
(Tsai, J. N. et al. 2017)[97]	USA.	White (100)	N/A	SC 20mcg Teriparatide daily for 48M	27	66.1±7.9	25.5±3.7
				SC 60mg Denosumab Q6M for 48M	27	65.1±6.2	23.8±4.1
(Saag. K. G. et al. 2017)[98]	USA.	Hispanic (32.3)	N/A	SC 210mg Romosozumab QM for 12M	1750	74.4±7.5	25.46±4.41
		Non-hispanic (67.7)		PO 70mg Alendronate QW for 12M	1757	74.2±7.5	25.36±4.42
(Liang. B. C. et al. 2017)[99]	China	Chinese (100)	9.78±1.45	IV 5mg Zoldronic acid Q12M for 24M	155	57.11±2.75	21.82±1.08
			9.98±1.55	Placebo	95	57.12±3.16	21.58±0.96
(Koh, J. M. et al. 2016)[100]	Korea	Korean (100)	19±7.02	SC 60mg Denosumab Q6M for 6M	68	67.0±4.86	23.5±2.83
			17.5±6.20	Placebo	66	66.0±4.77	23.7±2.29
(Kim, H. et al. 2016)[101]	International	White (66.3)	N/A	PO 0.8mg Calcitonin (SMC021) daily for 3yrs	2064	66.5±6.12	26.1±4.17
		Asian (12.9)		Placebo	2125	67.0±6.16	26.0±4.15

(Cosman, F. et al. 2016)[102]	International	Hispanic (39.4)<br>Non-hispanic (60.6)	N/A	Placebo<br>PO 210mg Romosozumab QM for 12M	61<br>65	70.8±6.9<br>70.9±7.0	24.74±4.42<br>24.66±4.30
(Miller, P. D. et al. 2016)[103]	International	White (79.8)<br>Asian (16.0)<br>Black (2.8)	20.6±8.3<br>19.9±8.1<br>20.4±8.2	SC 80ug Abaloparatide daily for 1yr<br>SC 20ug Teriparatide daily for 1yr<br>Placebo	652<br>704<br>694	68.9±6.5<br>68.8±6.6<br>68.7±6.5	25±3.5<br>25.2±3.6<br>25.1±3.6
(Zhang, Z. L. et al. 2015)[104]	China	Chinese (100)	N/A	PO 70mg Alendronate with 5600 IU D3 QW for 6M<br>0.25μg Calcitonin daily for 6M	95<br>101	65.6±8.0<br>64.8±7.4	23.0±3.5<br>22.7±2.9
(Palacios, S. et al. 2015)[105]	International	White (85.2)<br>Black (7.1)<br>Hispanic (5.2)<br>Other (2.5)	19.7±8.6<br>19.5±8.8	Oral 20mg Bazedoxifene daily for 7yrs<br>Placebo	421<br>441	66.5±6.5<br>66.5±6.8	26.6±3.8<br>26.3±3.8
(Leder, B. Z. et al. 2015)[106]	International	White (62.2)<br>Asian (24.4)<br>Other (13.3)	N/A	SC 20ug Teriparatide daily for 24weeks<br>Placebo	39<br>42	64.5±7.5<br>65.0±7.1	26±3.6<br>26±3.4
(Leder, B. Z. et al. 2014)[107]	International	White (100)	27±20<br>36±23	SC 60mg Teriparatide Q6M for 2yrs<br>SC 20ug Denosumab daily for 2yrs	28<br>31	65.5±7.9<br>66.3±8.3	25.5±3.8<br>24.1±3.9
(Abboskhujaeva, L. S. et al. 2014)[108]	N/A	N/A	N/A	Strontium ranelate<br>Placebo	16<br>16	63.3±1.5<br>62.9±1.52	30.4±1.06<br>31.2±1.14
(Kim, H. et al. 2013)[109]	Denmark<br>Estonia	N/A	N/A	Placebo<br>SC 20ug Teriparatide daily for 24weeks	29<br>31	65.8±6.2<br>66.4±7.1	25.23±3.4<br>24.79±4.14

(Rizzoli, R. et al. 2012)[110]	International	Caucasian (100)	N/A	2g Strontium ranelate daily for 24M<br>70mg Alendronate weekly for 24M	30<br>27	63.6±7.5<br>63.8±7.6	23.1±3.3<br>22.6±2.7
(Zhang, L. et al. 2012)[111]	China	Chinese (100)	14.7±7<br>13.5±7.2	SC 20ug rhPTH daily for 12M<br>IM 20 U Elactonin weekly for 12M	89<br>35	64.3±7.5<br>63.3±7.3	23.04±3.92<br>22.75±3
(Binkley, N. et al. 2012)[112]	USA.	N/A	N/A	Oral 0.2mg Calcitonin daily for 48 weeks<br>Placebo	189<br>82	66.5±7.6<br>66.5±8	25.63±4.44<br>25.67±3.79
(Cosman, F. et al. 2011)[113]	USA.	White (97.8)<br>Other (2.2)	N/A	SC 20ug Teriparatide/IV placebo daily for 52 wks<br>IV 5mg Zoldronic acid yearly for 52 wks	138<br>137	63.8±9.1<br>66.1±9	25.3±4.15<br>25.3±4.42
(Tuppurainen, M. et al. 2010)[114]	Finland	N/A	11.5±5.5<br>11.2±3.9	Oral 2mg/1mg Estradiol/Norethisterone daily for 5yrs<br>Placebo	45<br>51	60.9±2.8<br>60.9±2.5	25.6±3.6<br>25.9±4.1
(Finkelstein, J. S. et al. 2010)[115]	USA.	N/A	N/A	Oral 10mg Alendronate daily<br>SC 40ug Teriparatide daily	29<br>20	64±6<br>65±7	25.6±4.5<br>24.9±3.6
(Ensrud, K. et al. 2010)[116]	International	White (74.3)<br>Asian (18.3)<br>Other (7.5)	N/A	Oral 0.5mg Lasofoxifene daily for 5yrs<br>Placebo	2852<br>2852	67.3±5.2<br>67.5±5.2	25.4±3.7<br>25.4±3.8
(Cummings, S. R. et al. 2010)[117]	International	White (74.3)<br>Asian (18.3)<br>Other (7.5)	N/A	Oral 0.5mg Lasofoxifene daily for 5yrs<br>Placebo	2852<br>2852	67.3±5.2<br>67.5±5.2	25.4±3.7<br>25.4±3.8
(LaCroix, A. Z. et al. 2010)[118]	International	White (74.3)<br>Black (0.9)<br>Asian (18.3)	N/A	Oral 0.5mg Lasofoxifene daily for 5yrs<br>Placebo	2852<br>2852	67.3±5.2<br>67.5±5.2	25.4±3.7<br>25.4±3.8

		Hispanic 4.9)					
(Christiansen, C. et al. 2010)[119]	International	White (87.1)<br>Black (6.4)<br>Hispanic (4.7)<br>Other (1.9)	19.7±8.6<br>19.5±8.8	Oral 20mg Bazedoxifene daily for 3yrs<br>Placebo	1254<br>1256	66.5±6.5<br>66.5±6.8	26.6±3.8<br>26.3±3.8
(Yan, Y. et al. 2009)[120]	China	Chinese (100)	15.36±6.88<br>15.14±6.66	Oral 70mg Alendronate weekly for 12M<br>Placebo	280<br>280	65.19±6.47<br>64.66±5.87	23.12±2.89<br>23.31±3.13
(Cummings, S. R. et al. 2009)[121]	International	N/A	N/A	SC 60mg Denosumab Q6M for 36M<br>Placebo	3902<br>3906	72.3±5.2<br>72.3±5.2	26±4.1<br>26±4.2
(Silverman, S. L. et al. 2008)[122]	International	White (87.1)	19.5±8.7<br>19.5±8.8	Oral 60mg Raloxifene daily for 36M<br>Placebo	1252<br>1256	66.4±6.7<br>66.5±6.8	26.4±3.8<br>26.3±3.8
(Iwamoto, J. et al. 2008)[123]	Japan	Japanese (100)	N/A	Oral 5mg Alendronate daily for 12M<br>Oral 60mg Raloxifene daily for 12M	50<br>52	70.3±7.6<br>68.5±7.2	21.9±2.6<br>21.7±2.5
(Miyauchi, A. et al. 2008)[124]	Japan	Japanese (100)	23.32±7.1<br>19.49±3.8	SC 40ug Teriparatide daily over 24weeks<br>Placebo	27<br>34	72.5±6.1<br>69.9±3.6	21.59±3.68<br>21.36±2.61
(Hwang, J. S. et al. 2008)[125]	Taiwan	N/A	16.2±1.0<br>18.2±1.1	Oral 2g Stronium ranelate daily for 12M<br>Placebo	64<br>61	64.3±0.8<br>65.8±1	23.5±0.4<br>26.2±4.1
(Sethi, B. K. et al. 2008)[126]	India	N/A	15.7±6.4<br>16.0±7.6	SC 20ug Teriparatide daily for 6M<br>Placebo	38<br>35	61±6.3<br>63±6.3	26.2±4.1<br>25.3±3.9
(Greenspan, S. L. et al. 2007)[127]	International	White (83.8)<br>Hispanic (13.3)<br>Black (1.4)	18.1±9.5<br>18.2±9.5	SC 100ug PTH(1-84) daily for 18M<br>Placebo	824<br>877	64±7.4<br>64.3±7.4)	25.6±1.34<br>25.7±4.27

		Asian (0.7)					
(Black, D. M. et al. 2007)[128]	International	West Europe (30.1)<br>East Europe (20)<br>North America (19.8)<br>Latin America (16.1)<br>Asian (14)	N/A	IV(15min) 5mg Zoldronic acid yearly for 3yrs<br>Placebo	3248<br>3269	73.1±5.34)<br>73±5.4)	25.1±4.3<br>25.4±4.3
(Tanakol, R. et al. 2007)[129]	Turkey	N/A	154±91(M)<br>109±93(M)	Oral 400mg Clodronate daily for 36M<br>Placebo	30<br>49	59.3±5.9<br>55.4±9	25.1±4.7<br>26.9±5
(Kung, A. W. C. et al. 2006)[130]	International	Asian (100)	22.8±8.3<br>22.9±8.7	SC 20ug Teriparatide 1-34 daily for 6M<br>SC 100IU Calcitonin daily for 6M	39<br>41	70.6±7.1<br>70.6±6.6	N/A
(Hwang, J. S. et al. 2006)[131]	Taiwan	N/A	20.06±1.38<br>19.72±1.38	SC 20ug Teriparatide daily for 6M<br>Placebo	34<br>29	68.06±1.07<br>66.90±1.39	24.34±0.62<br>23.62±0.50
(Gonnelli, S. et al. 2006)[132]	Italy	N/A	N/A	SC 20ug Teriparatide 1-34 daily for 12M<br>Placebo	27<br>28	71.3±7.0<br>71.0±6.8	24.7±3.3<br>24.8±3.1
(Ensrud, K. et al. 2006)[133]	International	White (96)	18±8<br>18±8	Oral 60mg or 120mg Raloxifene daily for 4yrs<br>Placebo	2336<br>1106	66±7<br>66±7	25.2±3.9<br>25.2±3.9
(Martino, S. et al. 2005)[134]	International	White (96)	17.8±8<br>18±8	Oral 60mg or 120mg Raloxifene daily for 4yrs<br>Placebo	2725<br>1286	65.7±6.8<br>65.9±6.7	25.2±3.9<br>25.2±3.9
(McClung, M. R. et al. 2005)[135]	N/A	White (59)	19.9±9.6<br>19.5±10.3	Oral 10mg Alendronate daily for 18M<br>IV 20ug Teriparatide daily for 18M	101<br>102	66.6±8.5<br>65.3±8.4	25.3±4.5<br>25.7±4
(Leung, J. Y. Y. et al. 2005)[136]	China	N/A	15.5±1.6<br>15.5±2.2	Oral 5mg Risedronate daily for 18M<br>Placebo	31<br>34	67±6<br>67±6	N/A

(Ho, A. Y. Y. et al. 2005)[137]	China	N/A	11.6±5.8	Oral 70mg Alendronate weekly for 12M	29	60.6±5.5	22.4±3.2
			12±4.8	Placebo	29	62±4	23.1±2.8
(Dominguez, L. J. et al. 2005)[138]	N/A	N/A	N/A	IM 100mg Clodronate weekly for 12M	85	69.8±4.33	N/A
				Placebo	75	70.7±4.24	
(Li, Y. et al. 2005)[139]	China	N/A	N/A	Oral 5mg Risedronate daily for 12M	30	N/A	N/A
				Placebo	30		
(Kushida, K. et al. 2004)[140]	Japan	Japanese (100)	22±7.2	Oral 5mg Alendronate daily for 3yrs	90	71.2±5.3	N/A
			22.4±7.6	Oral 1ug Alfacalcidol daily for 3yrs	80	72.6±5.7	
(Ishida, Y. et al. 2004)[141]	Japan	Japanese (100)	N/A	Oral 0.625mg Estrogen daily for 24M	62	70±15	N/A
				Oral 200mg Etidronate daily for 24M	62	70±15	
				Oral 20IU Calcitonin weekly for 24M	62	69±17	
				Oral 1ug Alfacalcidol daily for 24M	63	71±12	
				Placebo	63	68±11	
					60	68±8	
(Adami, S. et al. 2004)[142]	Italy	N/A	17.6±6.4	IV 2mg Ibandronate Q3M for 12M	221	65.5±4.5	N/A
			17.7±7.3	Placebo	118	65.5±4.7	
(Ste-Marie, L. G. et al. 2004)[143]	Canada	N/A	23.9±7.9	Oral 5mg Risedronate daily for 60M	44	69.5±6.8	N/A
			23.3±10.1	Placebo	42	69.2±9.15	
(Meunier, P. J. et al. 2004)[144]	International	N/A	22.1±8.8	Oral 2g Stronium ranelate daily for 3yrs	628	69.4±7.2	26.1±4.1
			21.6±8.7	Placebo	632	69.2±7.3	26.2±4.1
(Luckey, M. et al. 2004)[145]	USA.	White (92.3)	17.8	Oral 60mg Raloxifene daily for 12M	193	64.7±9.8	25.3±4.8
		Black (2.4)	17.3	Oral 70mg Alendronate weekly for 12M	179	63.8±9.9	25.3±5.2

		Asian (1.5)<br>Other (3.7)					
(Genant, H. K. et al. 2004)[146]	USA.	N/A	21.4±6.4<br>21.3±6.8	Oral 60 or 120mg Raloxifene daily for 2yrs<br>Placebo	37<br>21	70.3±5.0<br>70.0±4.9	N/A
(Boonen, S. et al. 2004)[147]	International	N/A	N/A	Oral 5mg Risedronate daily for 3yrs<br>Placebo	704<br>688	83.0±3.1<br>83.0±3.0	24.9±4.6<br>24.5±4.1
(Barrett-Connor, E. et al. 2004)[148]	International	White (95.7)	N/A	Oral 60mg or 120mg Raloxifene daily for 4yrs<br>Placebo	5129<br>2576	66.4<br>66.6	25.2<br>25.2
(Martino, S. et al. 2004)[149]	International	White (95.5)	18.4±8.2	Oral 60mg Raloxifene daily for 8yrs<br>Placebo	2725<br>1286	66.2±6.9	25.3±3.9
(Morii, H. et al. 2003)[150]	Japan	Japanese (100)	15.8±6.7<br>14.4±6.3	Oral 120mg Raloxifene daily for 52weeks<br>Placebo	81<br>87	64.7±6.2<br>64.3±6.5	21.9±3<br>22±3
(Braga, V. et al. 2003)[151]	Italy	N/A	17.4±10.3<br>17.6±8.9	IV 50mg Neridronate Q2M for 36M<br>Placebo	39<br>39	63.4±8.7<br>65.7±6.6	23.6±3.7<br>23.5±3.4
(Hodsman, A. B. et al. 2003)[152]	USA, Canada	N/A	18±7<br>17±6	SC 100ug PTH(1-84) daily for 12M<br>Placebo	51<br>53	64±6<br>64±6	N/A
(Chailurkit, L. et al. 2003)[153]	Thailand	Thai (100)	12.6±7.0<br>12.8±6.6	Oral 10mg Alendronate daily for 12M<br>Placebo	32<br>38	62.2±6.4<br>61.8±5.6	22.3±2.9<br>22.4±3.5
(Meunier, P. J. et al. 2002)[154]	European countries	Caucasian	17.5±8.3<br>19.1±7.7	Oral 2g Stronium ranelate daily for 24M<br>Placebo	87<br>91	65.6±6.9<br>66.7±6.5	25.7±3.1<br>24.9±3.1
(Johnell, O. et al. 2002)[155]	International	N/A	15.6±7.7<br>16.5±7.7	Oral 60mg Raloxifene daily for 12M<br>Oral 10mg Alendronate daily for 12M	82<br>83	63.4±6.3<br>63.7±6.0	24.8±3.8<br>24.8±3.8

			17.6±8.2	Placebo	82	63.8±5.3	24.3±3.9
(Body, J. J. et al. 2002)[156]	International	N/A	18±9	SC 40ug Teriparatide 1-34 daily for 12M	73	66±8	23.9±4.5
			19±10	Oral 10mg Alendronate daily for 12M	73	65±9	24.4±3.5
(Rubin, C. D. et al. 2001)[157]	USA.	N/A	26±8	Oral 25mg Sodium Fluoride daily for 42M	44	73±5	25.5±4.3
			26±10	Placebo	41	73±5	24.6±4.3
(Riis, B. J. et al. 2001)[158]	Denmark	N/A	18.8±6.8	IV 2.5mg Ibandronate daily for 12M	81	66.8±4.9	N/A
			17.8±6.7	Placebo	81	66.3±4.8	
(Neer, R. M. et al. 2001)[159]	International	White (99)	21±8	SC 40ug Teriparatide 1-34 daily for 24M	497	70±7	26.6±4.3
			21±8	Placebo	504	67±7	26.7±4.7
(Iwamoto, J. et al. 2001)[160]	Japan	Japanese	17.0±1.3	Oral 200mg Etidronate daily for 24M	25	64.3±1.3	21.2±0.7
			18.3±1.5	Oral 45mg Menatetrenone daily for 24M	23	65.4±1.2	20.6±0.7
			16.0±1.2	Placebo	24	66±1.1	20.9±0.6
(Guanabens, N. et al. 2000)[161]	Spain	N/A	16.5	Oral 50mg Fluoride daily for 36M	31	64±7.96	26.6±3.73
			19	Oral 400mg Etidronate daily for 36M	47	65±6.92	27±5
(Bone, H. G. et al. 2000)[162]	USA.	Caucasian	22±8	Oral 10mg Alendronate daily for 24M	92	61±8	N/A
			21±8	Oral 0.625mg Estrogen daily for 24M	143	61±8	
			23±11	Placebo	50	62±9	
(Sahota, O. et al. 2000)[163]	UK.	N/A	17.4±5.1	Oral 10mg Alendronate daily for 12M	31	67.5±5.1	25.8±2.9
			17.2±4.2	Oral 250ng Calcitriol twice daily for 12M	33	67.2±4.2	26.8±3.1
(Downs, R. W. et al. 2000)[164]	USA.	White (98.3)	16.5±7.7	Oral 10mg Alendronate daily for 12M	118	64.6±6.8	25.6±4.2
			16.1±7.4	Intra-nasal 200 IU Calcitronin daily for 12M	123	64.1±7.2	25.9±4.6
			16.5±8.6	Placebo	58	64.6±6.8	25.4±4.2

(Chesnut, C. H. et al. 2000)[165]	USA, UK	N/A	21.9±8.4<br>22±9.4	Intra-nasal 400IU Calcitonin for 5yrs<br>Placebo	127<br>128	67.9±6.9<br>68.2±7.7	24.9±3.6<br>24.7±3.9
(Boivin, G. Y. et al. 2000)[166]	France	N/A	N/A	Oral 10mg Alendronate daily for 2yrs<br>Placebo	9<br>15	64±3<br>62±2	N/A
(Reginster, J. Y. et al. 2000)[167]	International	N/A	25±8.6<br>25±8.7	Oral 5mg Risedronate daily for 1yr<br>Placebo	251<br>221	71±7<br>71±7	N/A
(Kung, A. W. C. et al. 2000)[168]	China	Chinese	15±4<br>15±4	Alendronate<br>Placebo	35<br>35	64±5<br>65±4	23.2±3.3<br>23.1±2.3
(Tiras, M. B. et al. 2000)[169]	Turkey	N/A	4.9±4.6<br>6.5±5.6	Oral 2mg/1mg HRT daily for 12M<br>Oral 10mg Alendronate daily for 12M	31<br>32	52.7±5.6<br>53.8±6.8	24.2±3.6<br>23.8±4.1
(Rossini, M. et al. 1999)[170]	Italy	N/A	14±3<br>13±4	IM 100mg Clodronate weekly for 24M<br>Placebo	30<br>30	61±4<br>61±3	23.2±1.7<br>23.1±1.6
(Ringe, J. D. et al. 1999)[171]	Germany	N/A	N/A	Oral 20mg MFP/Ca daily for 3yrs<br>Placebo	30<br>33	65.5±6<br>63.7±6.1	N/A
(Harris S. T. et al. 1999)[172]	USA	American	24±10.1<br>24±10.0	Oral 5mg Risedronate daily for 3yrs<br>Placebo	425<br>398	69±7.7<br>68±7.2	N/A
(Reginster, J. Y. et al. 1998)[173]	Belgium	N/A	N/A	Oral 10mg MFP/Ca daily for 4yrs<br>Placebo	100<br>100	N/A	N/A
(Meunier, P. J. et al. 1998)[174]	France	N/A	18.1<br>18.8	Oral 50mg/150mg Fluoride(NaF/MFP) daily for 2yrs<br>Placebo	208<br>146	65.8<br>65.5	N/A

(Lufkin, E. G. et al. 1998)[175]	USA.	N/A	22±6.24	Oral 60mg Raloxifene daily for 12M	48	69.9±3.46	24.8±4.23
			22.2±1	Placebo	48	68.2±4.85	25.3±3.81
(Felsenberg, D. et al. 1998)[176]	Germany	N/A	N/A	Oral 10mg Alendronate daily for 1yr	152	64.1±6.7	N/A
				Placebo	145	63.3±7.5	
(Gonnelli, S. et al. 1997)[177]	Italy	N/A	6.5±4.4	Patch 10mg Estrogen daily for 2yr	40	56.2±4.6	N/A
			65.2±3.5	Placebo	41	56.3±4.5	
(Thiébaud, D. et al. 1997)[178]	Switzerland, Germany	N/A	18.3±1.6	IV 2mg Ibandronate Q3M for 12M	23	64±1.5	25.3±0.7
			17±1.3	Placebo	26	64.2±1.1	24.5±0.8
(Filiponni, P. et al. 1996)[179]	Italy	N/A	12.6±0.6	IM 200mg Clodronate every 3wk for 4yrs	44	62.1±0.6	N/A
			14.4±0.4	Placebo	37	63.9±0.7	
(Tucci, J. R. et al. 1996)[180]	USA.	N/A	17.1±8.5	Oral 10mg Alendronate daily for 3yrs	94	63.9±6.4	23.3±3
			17.8±8.2	Placebo	192	64.2±7.4	23.8±3.6
(Ravn, P. et al. 1996)[181]	Denmark	N/A	N/A	Oral 2.5mg Ibandronate daily for 12M	30	65.2±5.7	N/A
				Placebo	30	63.9±6	
(Ellerington, M. C. et al. 1996)[182]	UK.	Caucasian	N/A	Intra-nasal 200IU Calcitonin daily for 2yrs	29	55.4±3.9	N/A
				Placebo	39	56.1±4.3	
(Devogelaer, J. P. et al. 1996)[183]	International	N/A	16±7.7	Oral 10mg Alendronate daily for 3 yrs	102	63.2±6.6	24±3.2
			15.2±8.1	Placebo	205	62.7±7.2	24.4±3.5
(Pak, C. Y. C. et al. 1995)[184]	USA.	N/A	18.5	Oral 25mg SR-NaF/Ca twice daily for 2yrs	48	66.5±8.6	N/A
			19	Placebo	51	68.7±8.9	
(Liberman, U. A. et al. 1995)[185]	International	N/A	16	Oral 10mg Alendronate daily for 3yrs	196	64	24.2
			17	Placebo	397	64	24.1

(Chesnut, C. H. et al. 1995)[186]	USA.	N/A	15±6.9	Oral 10mg Alendronate daily for 24M	30	62.9±6.1	N/A
			16.9±7.7	Placebo	31	63.6±7.1	
(Adami, S. et al. 1995)[187]	Italy	N/A	12±7	Oral 10mg Alendronate daily for 24M	68	59±6	N/A
			11±6	Oral 100IU Calcitonin daily for 24M	75	60±6	
			11±8	Placebo	71	59±6	
(Thiébaud, D. et al. 1994)[188]	Switzerland	N/A	15.1±2.3	IV 30mg Pamidronate Q3M for 24M	16	64.6±2.7	N/A
			14.8±2.4	Oral 20-30mg Fluoride daily for 24M	16	67.2±2.1	
(IAN, R. R. et al. 1994)[189]	New Zealand	N/A	17±9	Oral 150mg Pamidronate daily for 2yrs	26	65±7	N/A
			19±10	Placebo	22	67±6	
(Rossini, M. et al. 1994)[190]	Italy	N/A	12±4	Oral 20mg Alendronate daily for 6M	15	63±3	25.6±3.9
			12±3	Placebo	15	62±2	23.6±3.8
(Adami, S. et al. 1993)[191]	Italy	N/A	12±7	Oral 10mg Alendronate daily for 2yrs	68	59±6	N/A
			11±6	Intra-nasal 100IU Calcitonin daily for 2yrs	75	60±6	
			11±8	Placebo	71	59±6	

Abbreviation: N/A, not applicable; IM, intramuscularly; SC, subcutaneously; M, month; QM, once a month; QW, once a week; Q6M, every 6 month; Yr, year

Table S4: Concerned Adverse Events Reported in Previous Studies

Name	Intervention	Major reported adverse events	subject	Follow up
(Watts, N. B. et al. 2019)[94]	SC 60mg Denosumab Q6M for 7yrs	ONJ	2343	10yrs
	Placebo for 3yrs and Denosumab for 7yrs		2207	
(Kendler, D. L. et al. 2018)[96]	SC 20ug Teriparatide daily for 24M	Death, hip fracture, wrist fracture, atypical femoral fracture, ONJ	498	24M
	Oral 35mg Risedronate weekly for 24M		515	
(Saag, K. G. et al. 2017)[98]	SC 210mg Romosozumab QM for 12M	Death, non-vertebral fracture, CVD, cancer, ONJ, hip fracture, atypical femoral fracture	1750	12M
	PO 70mg Alendronate QW for 12M		1757	
(Koh, J. M. et al. 2016)[100]	SC 60mg Denosumab Q6M for 6M	Death	68	6M
	Placebo		66	
(Cosman, F. et al. 2016)[102]	Placebo	Death, cancer, CVD, ONJ, non-vertebral fracture, atypical femoral fracture	61	12M
	PO 210mg Romosozumab QM for 12M		65	
(Miller, P. D. et al. 2016)[103]	SC 80ug Abaloparatide daily for 1yr	Death, non-vertebral fracture	652	1yr
	SC 20ug Teriparatide daily for 1yr		704	
	Placebo		694	
(Zhang, Z. L. et al. 2015)[104]	PO 70mg Alendronate with 5600 IU D3 QW for 6M	Death	95	6M
	0.25μg Calcitonin daily for 6M		101	

(Palacios, S. et al. 2015)[105]	Oral 20mg Bazedoxifene daily for 7yrs<br>Placebo	Death, CVD, breast cancer	421<br>441	7yrs
(Ensrud, K. et al. 2010)[116]	Oral 0.5mg Lasofoxifene daily for 5yrs<br>Placebo	CVD	2852<br>2852	5yrs
(Cummings, S. R. et al. 2010)[117]	Oral 0.5mg Lasofoxifene daily for 5yrs<br>Placebo	Death	2852<br>2852	5yrs
(LaCroix, A. Z. et al. 2010)[118]	Oral 0.5mg Lasofoxifene daily for 5yrs<br>Placebo	Breast cancer	2099<br>2112	5yrs
(Christiansen, C. et al. 2010)[119]	Oral 20mg Bazedoxifene daily for 3yrs<br>Placebo	Breast cancer	1254<br>1256	3yrs
(Cummings, S. R. et al. 2009)[121]	SC 60mg Denosumab Q6M for 36M<br>Placebo	Death, cancer, ONJ, CVD, non-vertebral fracture, hip fracture	N/A	36M
(Silverman, S. L. et al. 2008)[122]	Oral 20mg Bazedoxifene daily for 36M<br>Placebo	Death, breast cancer, non-vertebral fracture, venous thromboembolic event	1254<br>1256	36M
(Black, D. M. et al. 2007)[128]	IV(15min) 5mg Zoldronic acid yearly for 3yrs<br>Placebo	Death, stroke, non-vertebral fracture, hip fracture	3248<br>3269	3yrs
(Ensrud, K. et al. 2006)[133]	Oral 60mg or 120mg Raloxifene daily for 4yrs<br>Placebo	CVD, death (myocardial cause)	2336<br>1106	4yrs

(Martino, S. et al. 2005)[134]	Oral 60mg Raloxifene daily for 8yrs<br>Placebo	Death, cancer, breast cancer, venous thromboembolic event	2725<br>1286	8yrs
(Adami, S. et al. 2004)[142]	IV 2mg Ibandronate Q3M for 12M<br>Placebo	CVD	221<br>118	12M
(Boonen, S. et al. 2004)[147]	Oral 5mg Risedronate daily for 3yrs<br>Placebo	Death	704<br>688	3yrs
(Barrett-Connor, E. et al. 2004)[148]	Oral 60mg or 120mg Raloxifene daily for 4yrs<br>Placebo	Hip fracture, death, invasive breast cancer	5129<br>2576	4yrs
(Martino, S. et al. 2004)[149]	Oral 60mg Raloxifene daily for 8yrs<br>Placebo	Breast cancer, death	2725<br>1286	8yrs
(Morii, H. et al. 2003)[150]	Oral 120mg Raloxifene daily for 52weeks<br>Placebo	CVD	81<br>87	52wks
(Neer, R. M. et al. 2001)[159]	SC 40ug Teriparatide 1-34 daily for 24M<br>Placebo	Non-vertebral fracture, wrist fracture, hip fracture	497<br>504	24M
(Chesnut, C. H. et al. 2000)[165]	Intra-nasal 400IU Calcitonin for 5yrs<br>Placebo	Non-vertebral fracture, hip fracture	127<br>128	5yrs
(Reginster, J. Y. et al. 2000)[167]	Oral 5mg Risedronate daily for 1yr<br>Placebo	CVD, non-vertebral fracture, cancer	251<br>221	1yr
(Ringe, J. D. et al. 1999)[171]	Oral 20mg MFP/Ca daily for 3yrs<br>Placebo	Hip fracture, non-vertebral fracture	30<br>33	3yrs
(Meunier, P. J. et al. 1998)[174]	Oral 50mg/150mg Fluoride daily for 2yrs<br>Placebo	Hip fracture, non-vertebral fracture	208<br>146	2yrs

(Lufkin, E. G. et al. 1998)[175]	Oral 60mg Raloxifene daily for 12M<br>Placebo	Non-vertebral fracture, hip fracture, wrist fracture	48<br>48	12M
(Pak, C. Y. C. et al. 1995)[184]	Oral 25mg SR-NaF/Ca twice daily for 2yrs<br>Placebo	Hip fracture	48<br>51	2yrs
(Liberman, U. A. et al. 1995)[185]	Oral 10mg Alendronate daily for 3yrs<br>Placebo	Non-vertebral fracture, hip fracture	196<br>397	3yrs

Abbreviation: N/A, not applicable; SC, subcutaneously; M, month; QM, once a month; QW, once a week; Q6M, every 6 month; Yr, year

Figure S1. Risk of Bias Assessment Summary Graph (Review Authors' Judgment as Low, Unclear, or High for Each Risk of Bias Item) Shown as Percentages Across All Included Studies

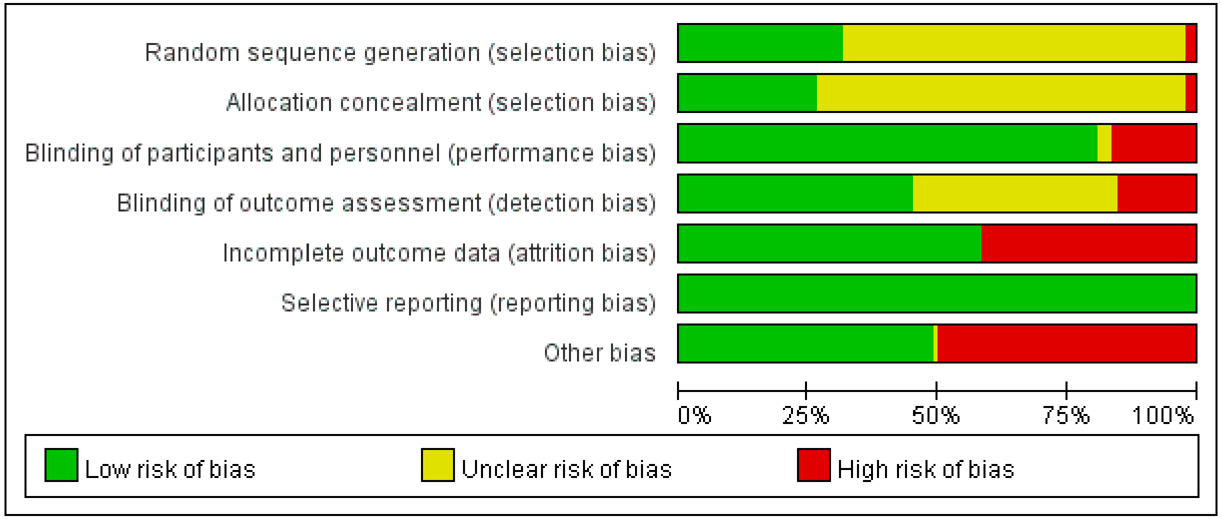

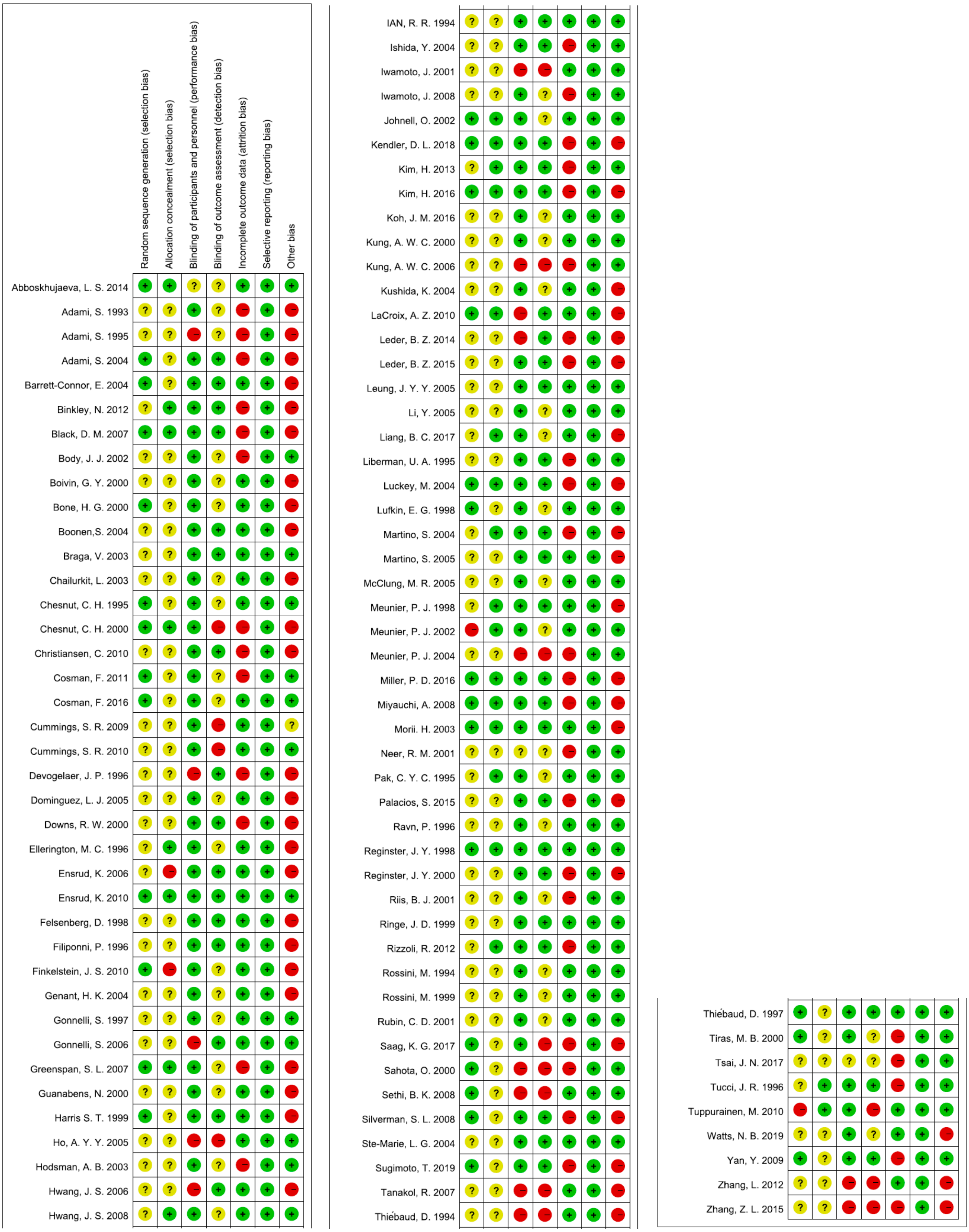

Random sequence generation (selection bias)
Allocation concealment (selection bias)
Blinding of participants and personnel (performance bias)
Blinding of outcome assessment (detection bias)
Incomplete outcome data (attrition bias)
Selective reporting (reporting bias)
Other bias
Abboskhujaeva, L. S. 2014
Adami, S. 1993
Adami, S. 1995
Adami, S. 2004
Barrett-Connor, E. 2004
Binkley, N. 2012
Black, D. M. 2007
Body, J. J. 2002
Boivin, G. Y. 2000
Bone, H. G. 2000
Boonen,S. 2004
Braga, V. 2003
Chailurkit, L. 2003
Chesnut, C. H. 1995
Chesnut, C. H. 2000
Christiansen, C. 2010
Cosman, F. 2011
Cosman, F. 2016
Cummings, S. R. 2009
Cummings, S. R. 2010
Devogelaer, J. P. 1996
Dominguez, L. J. 2005
Downs, R. W. 2000
Ellerington, M. C. 1996
Ensrud, K. 2006
Ensrud, K. 2010
Felsenberg, D. 1998
Filiponni, P. 1996
Finkelstein, J. S. 2010
Genant, H. K. 2004
Gonnelli, S. 1997
Gonnelli, S. 2006
Greenspan, S. L. 2007
Guanabens, N. 2000
Harris S. T. 1999
Ho, A. Y. Y. 2005
Hodsman, A. B. 2003
Hwang, J. S. 2006
Hwang, J. S. 2008
IAN, R. R. 1994
Ishida, Y. 2004
Iwamoto, J. 2001
Iwamoto, J. 2008
Johnell, O. 2002
Kendler, D. L. 2018
Kim, H. 2013
Kim, H. 2016
Koh, J. M. 2016
Kung, A. W. C. 2000
Kung, A. W. C. 2006
Kushida, K. 2004
LaCroix, A. Z. 2010
Leder, B. Z. 2014
Leder, B. Z. 2015
Leung, J. Y. Y. 2005
Li, Y. 2005
Liang, B. C. 2017
Liberman, U. A. 1995
Luckey, M. 2004
Lufkin, E. G. 1998
Martino, S. 2004
Martino, S. 2005
McClung, M. R. 2005
Meunier, P. J. 1998
Meunier, P. J. 2002
Meunier, P. J. 2004
Miller, P. D. 2016
Miyauchi, A. 2008
Morii. H. 2003
Neer, R. M. 2001
Pak, C. Y. C. 1995
Palacios, S. 2015
Ravn, P. 1996
Reginster, J. Y. 1998
Reginster, J. Y. 2000
Riis, B. J. 2001
Ringe, J. D. 1999
Rizzoli, R. 2012
Rossini, M. 1994
Rossini, M. 1999
Rubin, C. D. 2001
Saag, K. G. 2017
Sahota, O. 2000
Sethi, B. K. 2008
Silverman, S. L. 2008
Ste-Marie, L. G. 2004
Sugimoto, T. 2019
Tanakol, R. 2007
Thiébaud, D. 1994
Thiébaud, D. 1997
Tiras, M. B. 2000
Tsai, J. N. 2017
Tucci, J. R. 1996
Tuppurainen, M. 2010
Watts, N. B. 2019
Yan, Y. 2009
Zhang, L. 2012
Zhang, Z. L. 2015

Figure S2. Forest Plots of Direct Pairwise Comparisons for BMD at **(A)** lumbar spine (LS) **(B)** total hip (TH) **(C)** radius (RU)

(A) Lumbar spine (LS)

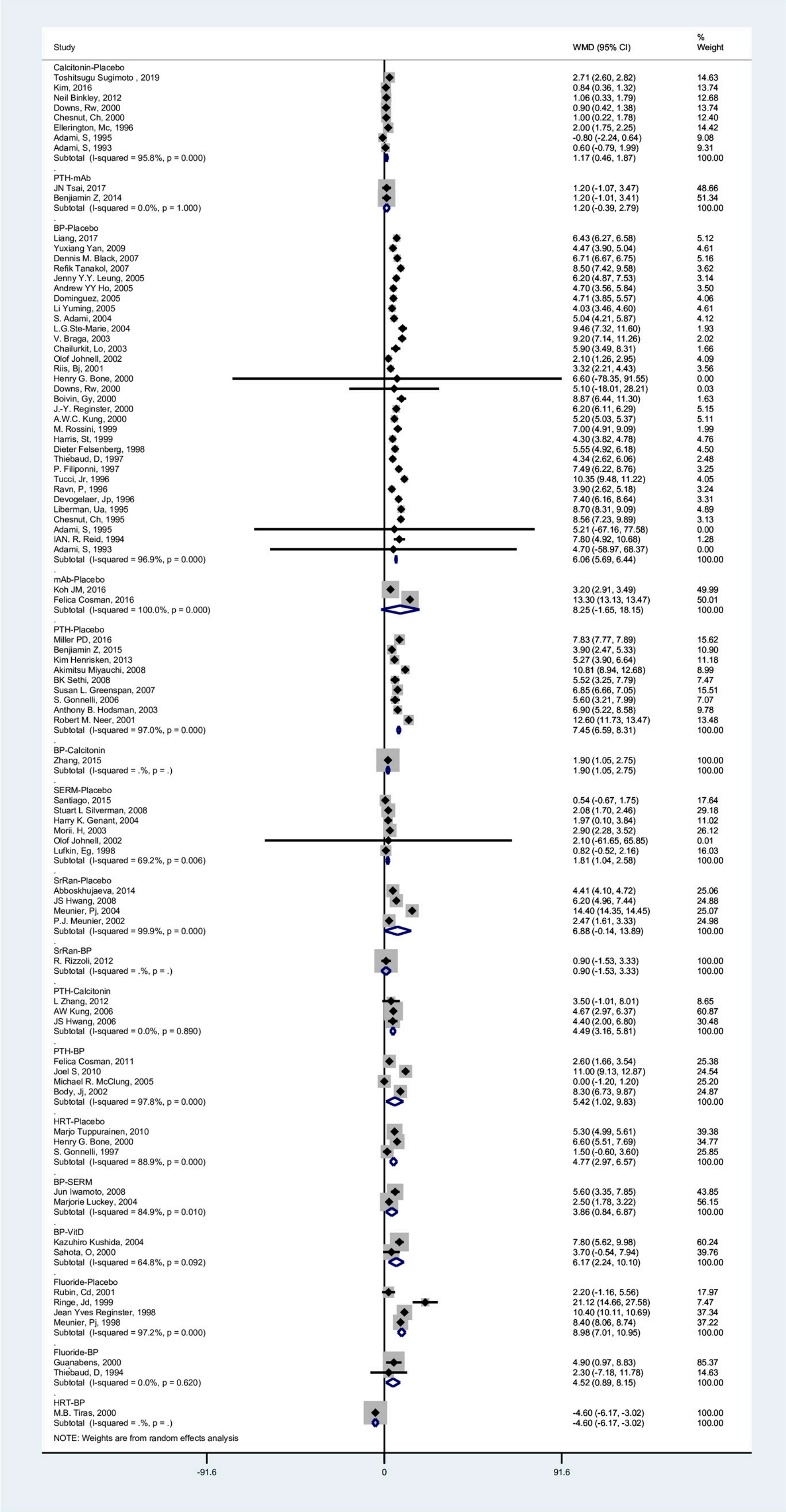

(B) Total hip (TH)

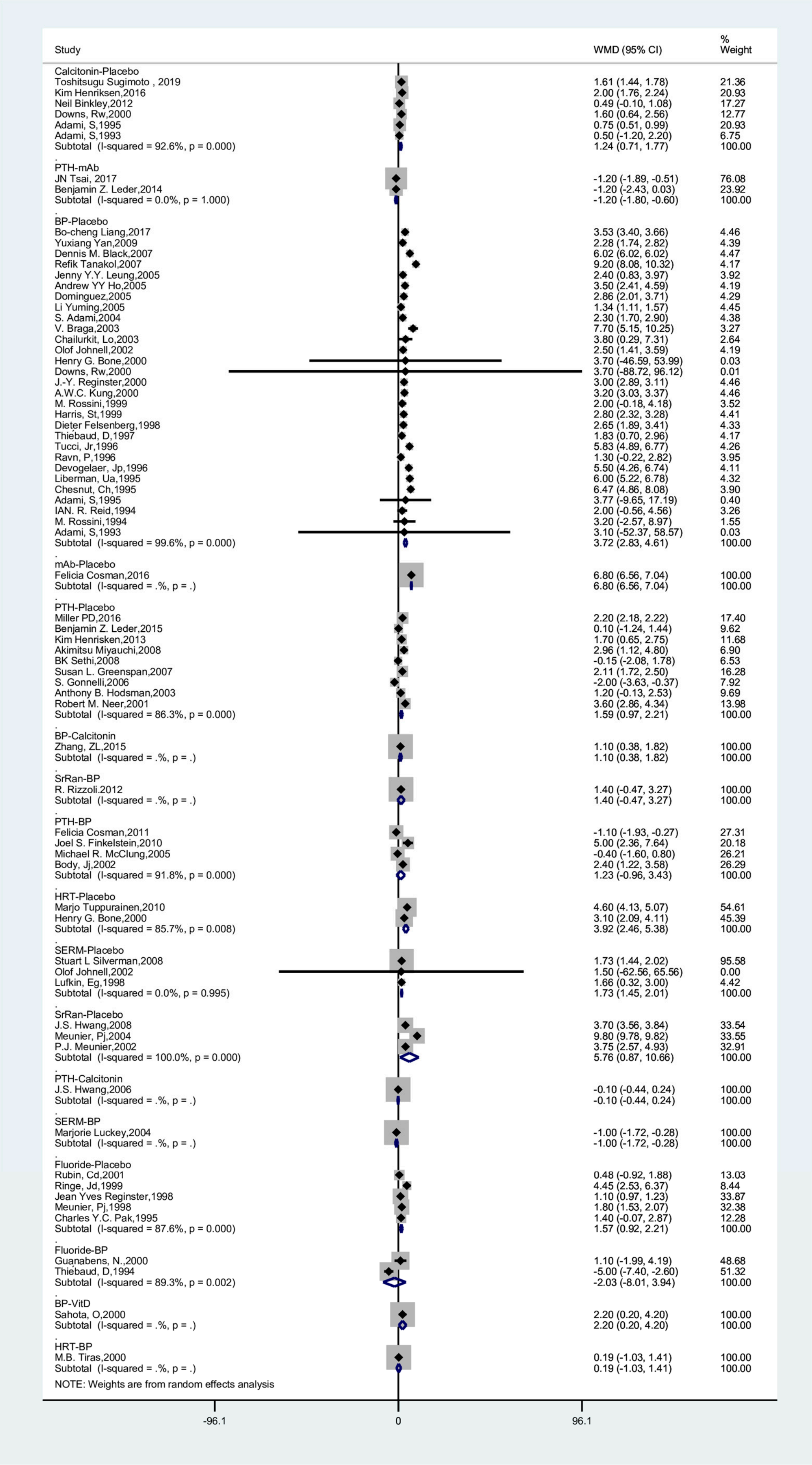

(C) Radius (RU)

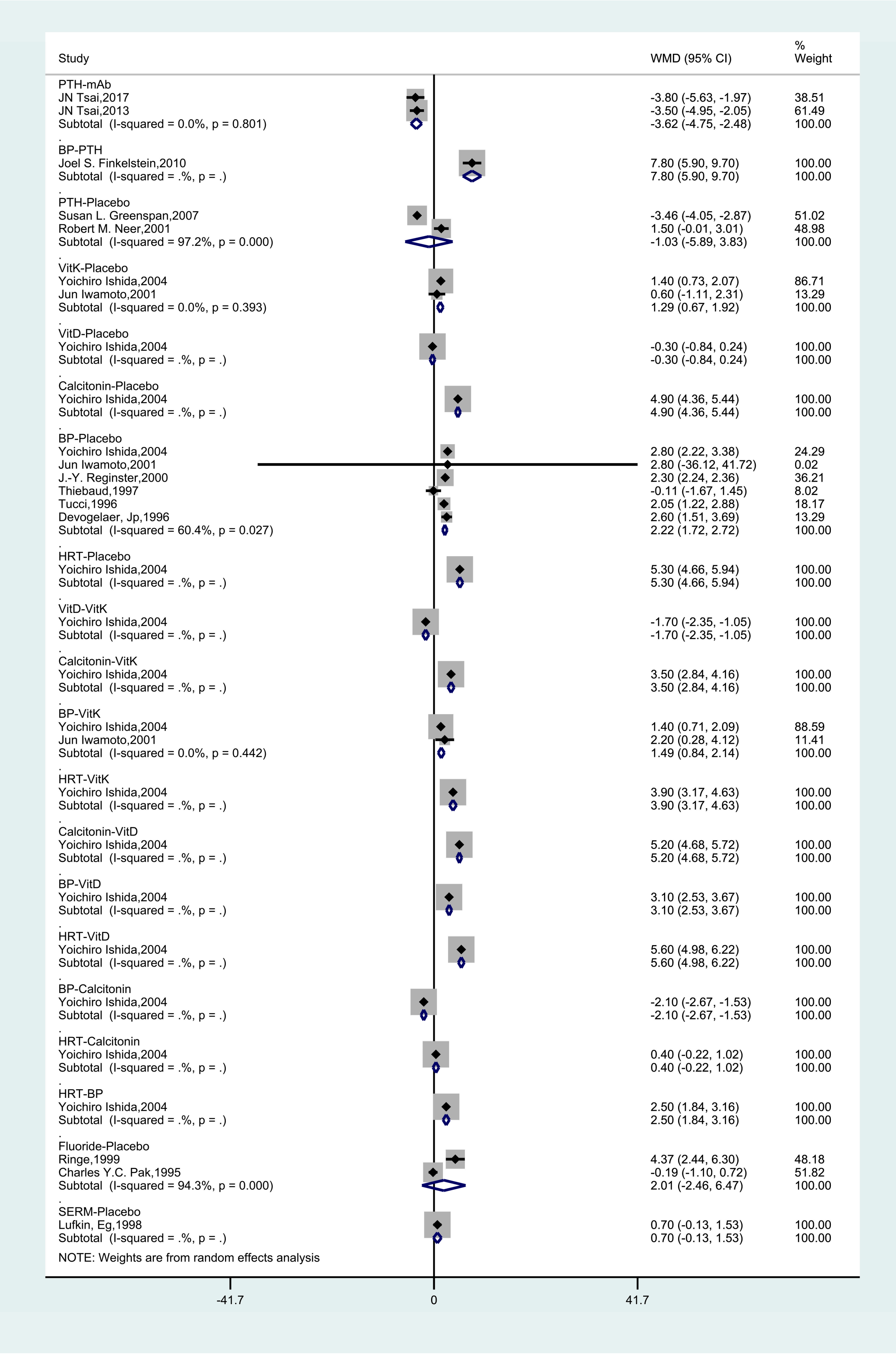
Study
WMD (95% CI)
% Weight
PTH-mAb
JN Tsai,2017 -3.80 (-5.63, -1.97) 38.51
JN Tsai,2013 -3.50 (-4.95, -2.05) 61.49
Subtotal (I-squared = 0.0%, p = 0.801) -3.62 (-4.75, -2.48) 100.00
.
BP-PTH
Joel S. Finkelstein,2010 7.80 (5.90, 9.70) 100.00
Subtotal (I-squared = .%, p = .) 7.80 (5.90, 9.70) 100.00
.
PTH-Placebo
Susan L. Greenspan,2007 -3.46 (-4.05, -2.87) 51.02
Robert M. Neer,2001 1.50 (-0.01, 3.01) 48.98
Subtotal (I-squared = 97.2%, p = 0.000) -1.03 (-5.89, 3.83) 100.00
.
VitK-Placebo
Yoichiro Ishida,2004 1.40 (0.73, 2.07) 86.71
Jun Iwamoto,2001 0.60 (-1.11, 2.31) 13.29
Subtotal (I-squared = 0.0%, p = 0.393) 1.29 (0.67, 1.92) 100.00
.
VitD-Placebo
Yoichiro Ishida,2004 -0.30 (-0.84, 0.24) 100.00
Subtotal (I-squared = .%, p = .) -0.30 (-0.84, 0.24) 100.00
.
Calcitonin-Placebo
Yoichiro Ishida,2004 4.90 (4.36, 5.44) 100.00
Subtotal (I-squared = .%, p = .) 4.90 (4.36, 5.44) 100.00
.
BP-Placebo
Yoichiro Ishida,2004 2.80 (2.22, 3.38) 24.29
Jun Iwamoto,2001 2.80 (-36.12, 41.72) 0.02
J.-Y. Reginster,2000 2.30 (2.24, 2.36) 36.21
Thiebaud,1997 -0.11 (-1.67, 1.45) 8.02
Tucci,1996 2.05 (1.22, 2.88) 18.17
Devogelaer, Jp,1996 2.60 (1.51, 3.69) 13.29
Subtotal (I-squared = 60.4%, p = 0.027) 2.22 (1.72, 2.72) 100.00
.
HRT-Placebo
Yoichiro Ishida,2004 5.30 (4.66, 5.94) 100.00
Subtotal (I-squared = .%, p = .) 5.30 (4.66, 5.94) 100.00
.
VitD-VitK
Yoichiro Ishida,2004 -1.70 (-2.35, -1.05) 100.00
Subtotal (I-squared = .%, p = .) -1.70 (-2.35, -1.05) 100.00
.
Calcitonin-VitK
Yoichiro Ishida,2004 3.50 (2.84, 4.16) 100.00
Subtotal (I-squared = .%, p = .) 3.50 (2.84, 4.16) 100.00
.
BP-VitK
Yoichiro Ishida,2004 1.40 (0.71, 2.09) 88.59
Jun Iwamoto,2001 2.20 (0.28, 4.12) 11.41
Subtotal (I-squared = 0.0%, p = 0.442) 1.49 (0.84, 2.14) 100.00
.
HRT-VitK
Yoichiro Ishida,2004 3.90 (3.17, 4.63) 100.00
Subtotal (I-squared = .%, p = .) 3.90 (3.17, 4.63) 100.00
.
Calcitonin-VitD
Yoichiro Ishida,2004 5.20 (4.68, 5.72) 100.00
Subtotal (I-squared = .%, p = .) 5.20 (4.68, 5.72) 100.00
.
BP-VitD
Yoichiro Ishida,2004 3.10 (2.53, 3.67) 100.00
Subtotal (I-squared = .%, p = .) 3.10 (2.53, 3.67) 100.00
.
HRT-VitD
Yoichiro Ishida,2004 5.60 (4.98, 6.22) 100.00
Subtotal (I-squared = .%, p = .) 5.60 (4.98, 6.22) 100.00
.
BP-Calcitonin
Yoichiro Ishida,2004 -2.10 (-2.67, -1.53) 100.00
Subtotal (I-squared = .%, p = .) -2.10 (-2.67, -1.53) 100.00
.
HRT-Calcitonin
Yoichiro Ishida,2004 0.40 (-0.22, 1.02) 100.00
Subtotal (I-squared = .%, p = .) 0.40 (-0.22, 1.02) 100.00
.
HRT-BP
Yoichiro Ishida,2004 2.50 (1.84, 3.16) 100.00
Subtotal (I-squared = .%, p = .) 2.50 (1.84, 3.16) 100.00
.
Fluoride-Placebo
Ringe,1999 4.37 (2.44, 6.30) 48.18
Charles Y.C. Pak,1995 -0.19 (-1.10, 0.72) 51.82
Subtotal (I-squared = 94.3%, p = 0.000) 2.01 (-2.46, 6.47) 100.00
.
SERM-Placebo
Lufkin, Eg,1998 0.70 (-0.13, 1.53) 100.00
Subtotal (I-squared = .%, p = .) 0.70 (-0.13, 1.53) 100.00
NOTE: Weights are from random effects analysis
-41.7
0
41.7

Figure S3: Forest Plots of Direct Pairwise Comparisons for incidence of adverse events including **(A)** cancer **(B)** cardiovascular disease (CVD) **(C)** hip fracture and **(D)** death.

(A) Cancer

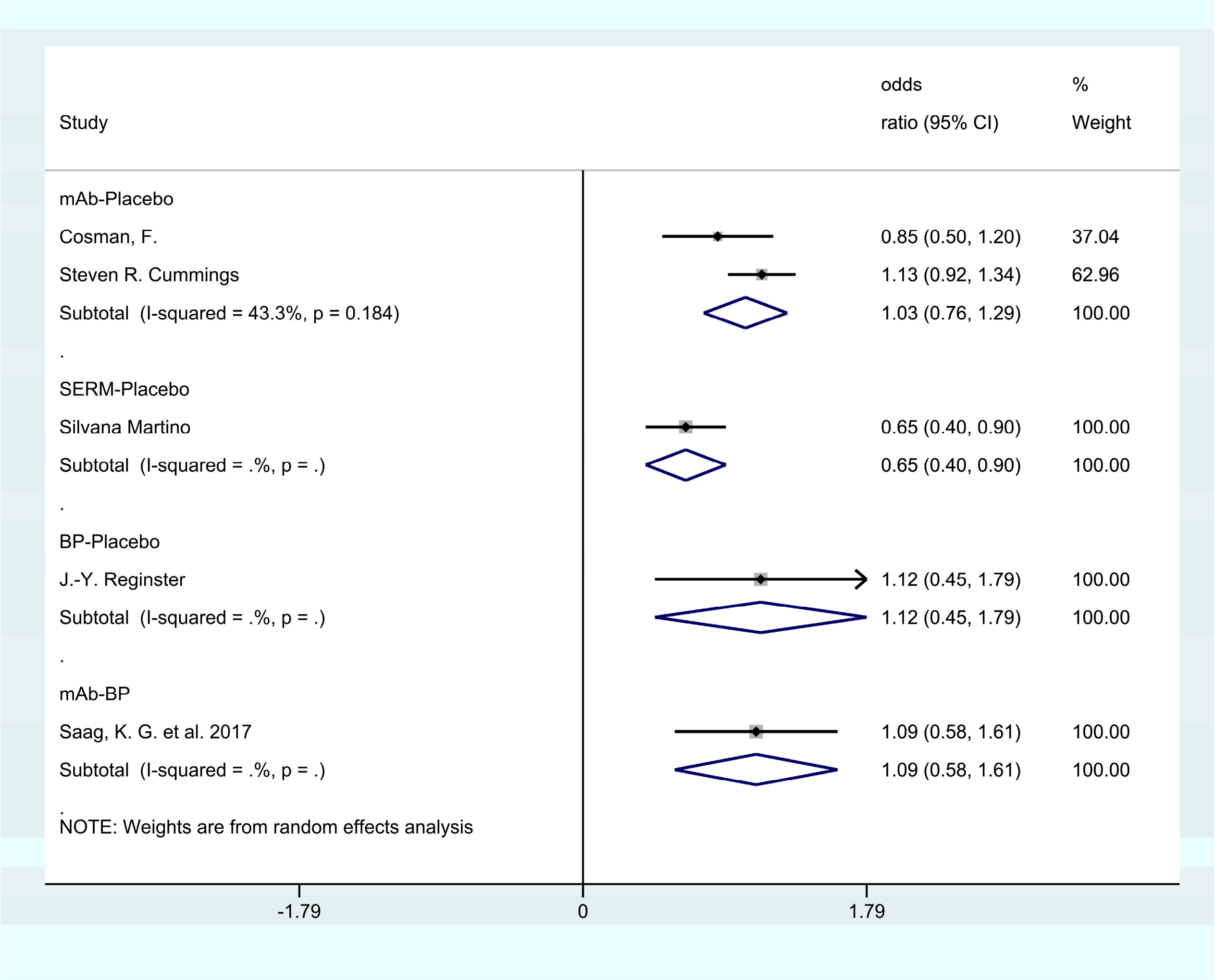

(B) Cardiovascular disease (CVD)

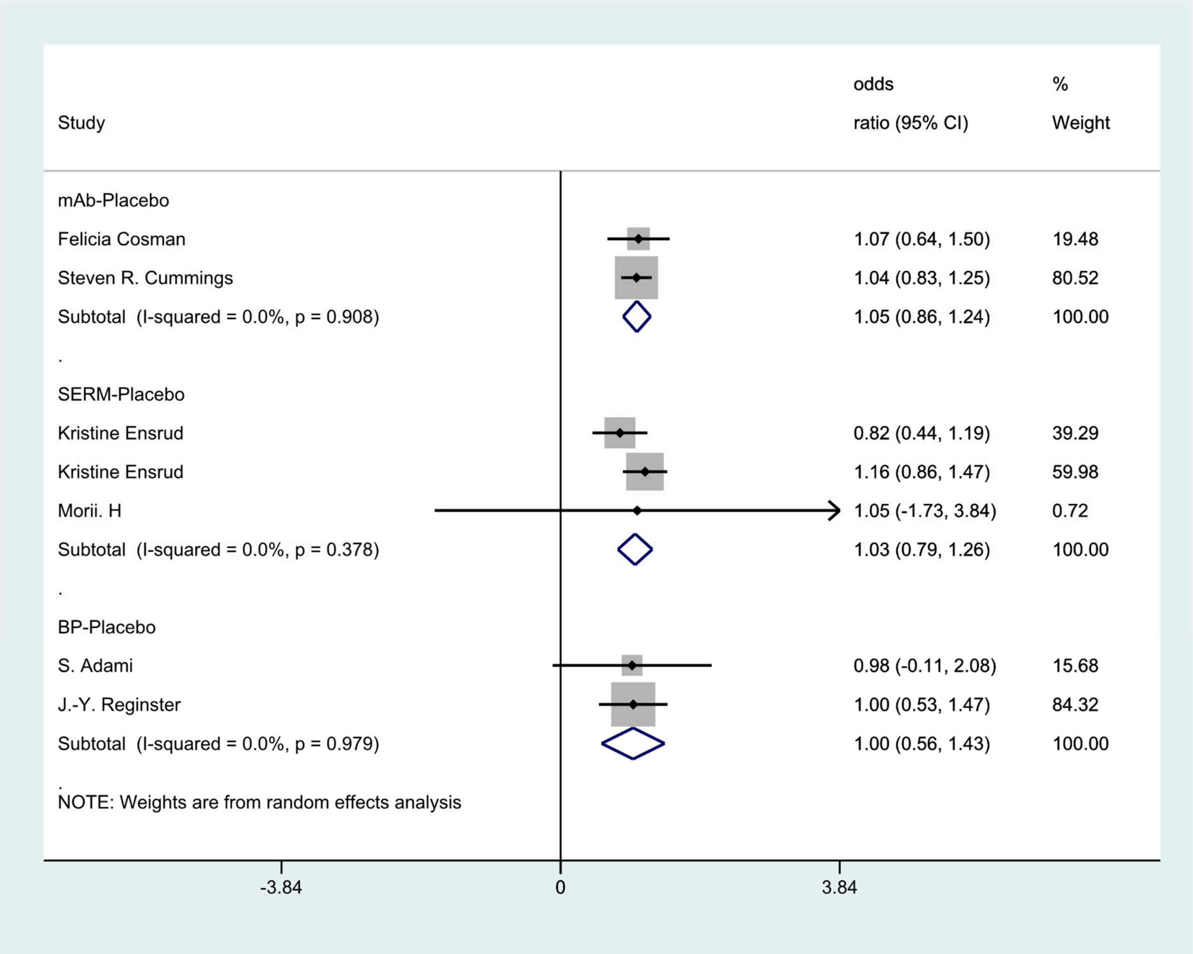

(C) Hip fracture

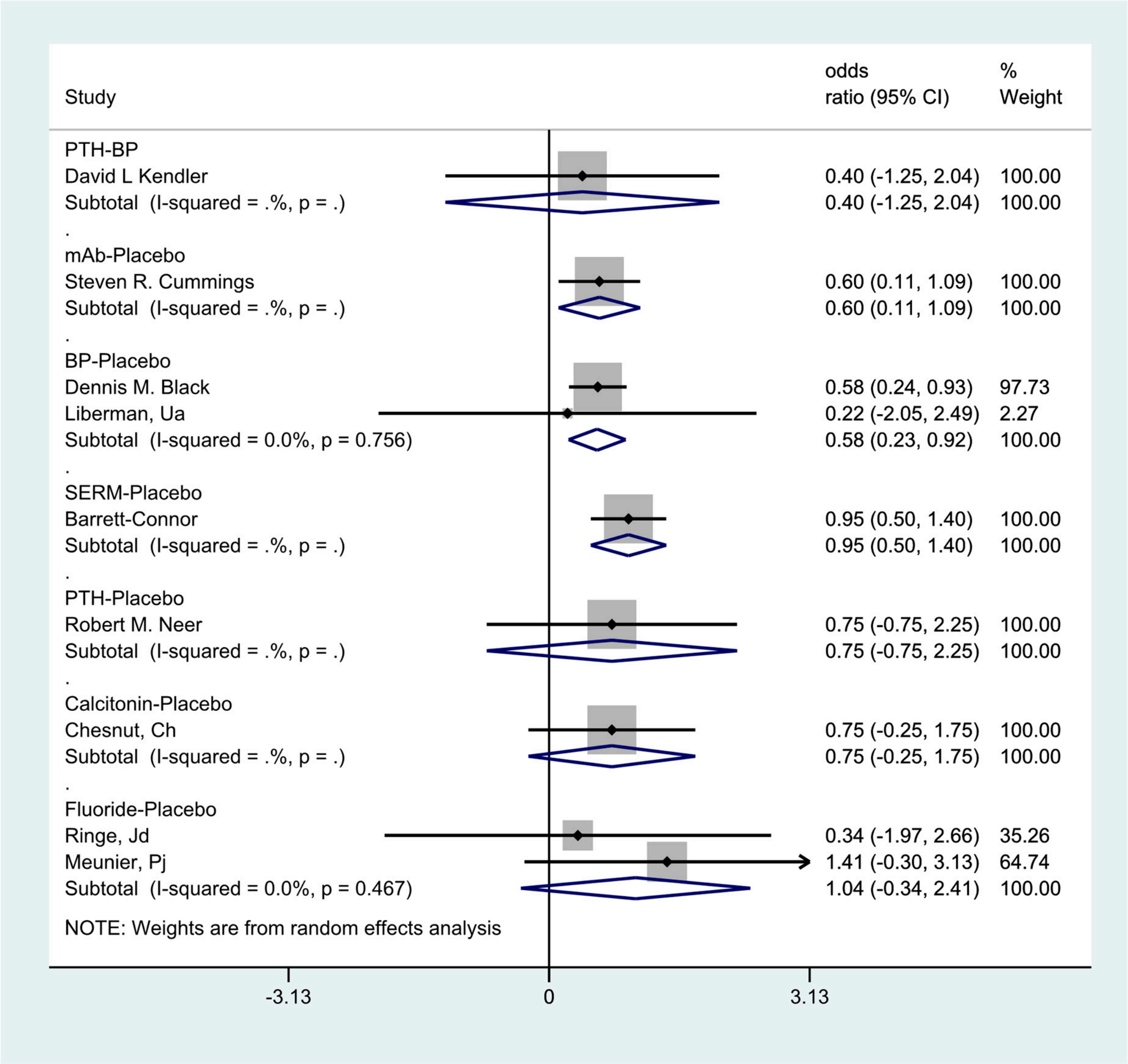

(D) Death

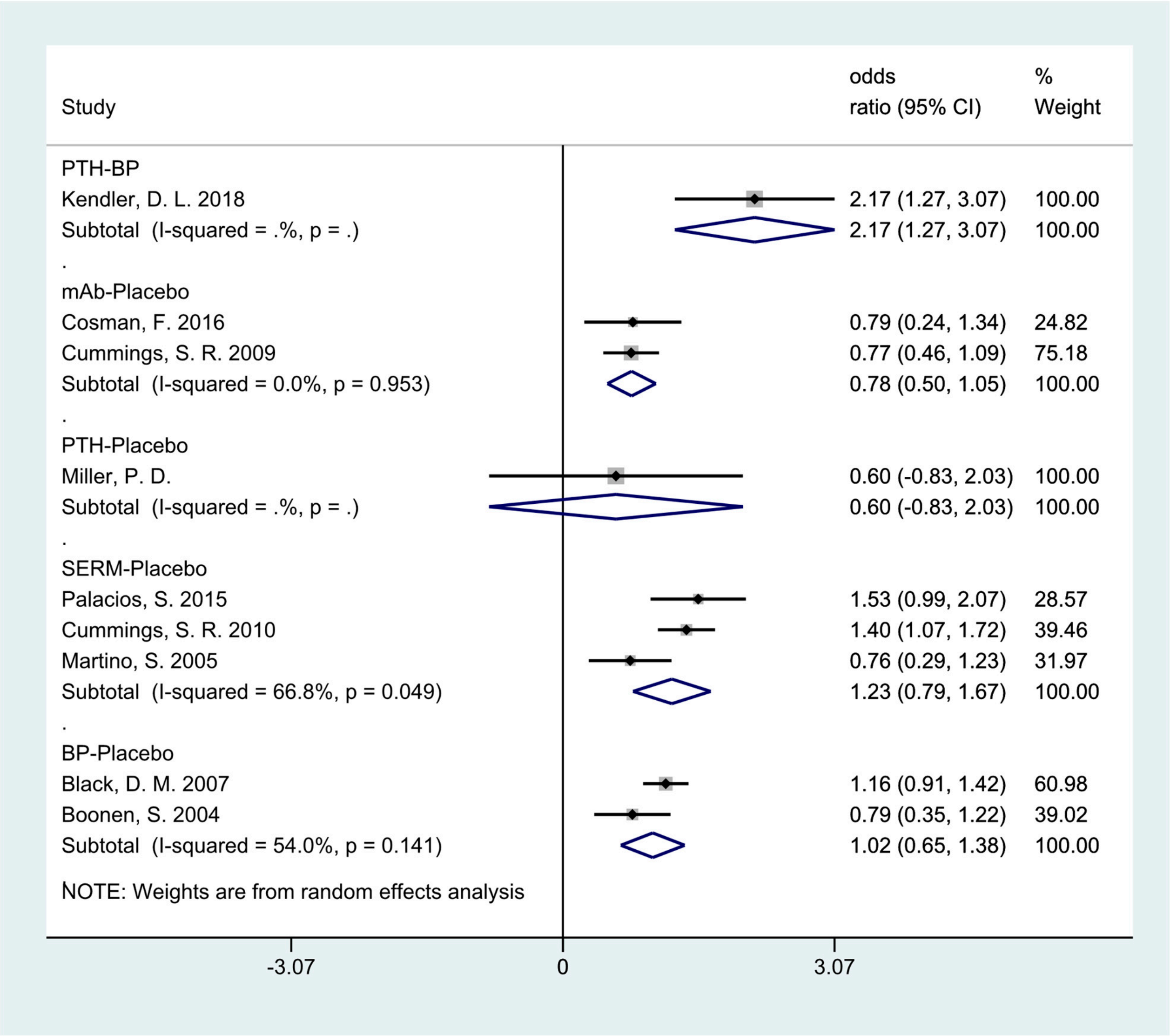

Study
odds ratio (95% CI)
% Weight
PTH-BP
Kendler, D. L. 2018
2.17 (1.27, 3.07)
100.00
Subtotal (I-squared = .%, p = .)
2.17 (1.27, 3.07)
100.00
mAb-Placebo
Cosman, F. 2016
0.79 (0.24, 1.34)
24.82
Cummings, S. R. 2009
0.77 (0.46, 1.09)
75.18
Subtotal (I-squared = 0.0%, p = 0.953)
0.78 (0.50, 1.05)
100.00
PTH-Placebo
Miller, P. D.
0.60 (-0.83, 2.03)
100.00
Subtotal (I-squared = .%, p = .)
0.60 (-0.83, 2.03)
100.00
SERM-Placebo
Palacios, S. 2015
1.53 (0.99, 2.07)
28.57
Cummings, S. R. 2010
1.40 (1.07, 1.72)
39.46
Martino, S. 2005
0.76 (0.29, 1.23)
31.97
Subtotal (I-squared = 66.8%, p = 0.049)
1.23 (0.79, 1.67)
100.00
BP-Placebo
Black, D. M. 2007
1.16 (0.91, 1.42)
60.98
Boonen, S. 2004
0.79 (0.35, 1.22)
39.02
Subtotal (I-squared = 54.0%, p = 0.141)
1.02 (0.65, 1.38)
100.00
NOTE: Weights are from random effects analysis
-3.07
0
3.07

Figure S4: The Surface Under the Cumulative Ranking Curves (SUCRAs) for the outcomes of individual interventions including BMD **(A)** at lumbar spine (LS), **(B)** total hip (TH), **(C)** radius (RU) and incidence of **(D)** cancer, **(E)** cardiovascular disease (CVD), **(F)** hip fracture, and **(G)** death.

(A) BMD at lumbar spine (LS)

Treatment	SUCRA	PrBest	MeanRank
Placebo	6.5	0.0	9.4
BP	57.8	0.0	4.8
mAb	79.4	12.8	2.9
HRT	43.1	0.0	6.1
SERM	27.1	0.0	7.6
PTH	85.6	13.1	2.3
Calcitonin	27.0	0.0	7.6
SrRan	69.5	3.1	3.7
Fluoride	94.9	71.0	1.5
VitD	9.2	0.0	9.2

(B) BMD at total hip (TH)

Treatment	SUCRA	PrBest	MeanRank
Placebo	3.5	0.0	9.7
BP	70.3	0.0	3.7
mAb	86.8	26.6	2.2
HRT	70.7	4.2	3.6
SERM	37.7	0.0	6.6
PTH	47.9	0.0	5.7
Calcitonin	25.9	0.0	7.7
SrRan	95.6	66.6	1.4
Fluoride	31.4	0.0	7.2
VitD	30.1	2.6	7.3

(C) BMD at radius (RU)

Treatment	SUCRA	PrBest	MeanRank
Placebo	27.9	0.0	7.5
BP	68.8	0.9	3.8
mAb	50.9	2.7	5.4
HRT	93.0	55.2	1.6
SERM	41.8	1.1	6.2
PTH	3.3	0.0	9.7
Calcitonin	90.1	37.4	1.9
Fluoride	57.6	2.4	4.8
VitD	22.8	0.0	7.9
VitK	43.9	0.3	6.1

(D) Incidence of Cancer

Treatm~t	SUCRA	PrBest	MeanRank
Placebo	61.6	21.9	2.2
BP	62.6	39.3	2.1
mAb	73.4	38.8	1.8
SERM	2.4	0.0	3.9

(E) Incidence of cardiovascular disease (CVD)

Treatm~t	SUCRA	PrBest	MeanRank
Placebo	47.1	9.0	2.6
BP	47.4	33.7	2.6
mAb	65.1	39.3	2.0
SERM	40.4	18.0	2.8

(F) incidence of hip fracture

Treatment	SUCRA	PrBest	MeanRank
Placebo	81.2	25.0	2.1
BP	31.1	0.0	5.1
mAb	35.4	0.7	4.9
SERM	75.0	24.0	2.5
PTH	17.4	2.1	6.0
Calcitonin	54.2	21.3	3.8
Fluoride	55.8	26.9	3.7

(G) Incidence of Death

Treatm~t	SUCRA	PrBest	MeanRank
Placebo	47.2	2.5	3.1
BP	37.6	3.0	3.5
mAb	15.5	1.2	4.4
SERM	72.4	31.5	2.1
PTH	77.3	61.8	1.9

Figure S5. Assessment of Inconsistency Results Between Direct and Indirect Evidence for the Outcomes of BMD at **(A)** Lumbar Spine (LS) **(B)** Total Hip (TH) **(C)** Radius (RU) and Incidence of **(D)** Cancer **(E)** Hip fracture **(F)** Death within the Networks Using the Design-by-Treatment Interaction Models, Loop Inconsistency Models, and Side-splitting Models.

(A) BMD at lumbar spine (LS)

Design-by-Treatment Interaction Model

A: Placebo; B: bisphosphonate (BP); C: monoclonal antibody (mAb); D: hormone replacement therapy (HRT); E: selective estrogen receptor modulator (SERM); F: parathyroid hormone (PTH); G: Calcitonin; H: strontium ranelate (SrRan); I: Fluoride; J: active vitamin D (VitD)

```
Testing for inconsistency:
 ( 1)  [_y_B]des_ABD = 0
 ( 2)  [_y_B]des_ABE = 0
 ( 3)  [_y_B]des_ABG = 0
 ( 4)  [_y_D]des_AD = 0
 ( 5)  [_y_E]des_AE = 0
 ( 6)  [_y_G]des_AG = 0
 ( 7)  [_y_D]des_BD = 0
 ( 8)  [_y_E]des_BE = 0
 ( 9)  [_y_F]des_BF = 0
 (10)  [_y_G]des_BG = 0
 (11)  [_y_H]des_BH = 0
 (12)  [_y_I]des_BI = 0
 (13)  [_y_F]des_CF = 0
 (14)  [_y_F]des_FG = 0

           chi2( 14) =   15.43
         Prob > chi2 =    0.3492
```

	Coef.	Std. Err.	z	P>|z|	[95% Conf.	Interval]
_y_B						
des_ABD	.2106389	2.655795	0.08	0.937	-4.994623	5.415901
des_ABE	-2.085361	2.607796	-0.80	0.424	-7.196547	3.025825
des_ABG	-1.382632	1.586424	-0.87	0.383	-4.491966	1.726702
_cons	6.389361	.5050656	12.65	0.000	5.399451	7.379271
_y_C						
_cons	8.257541	1.777056	4.65	0.000	4.774575	11.74051
_y_D						
des_AD	-3.027875	3.187885	-0.95	0.342	-9.276015	3.220266
des_BD	-4.80965	3.757999	-1.28	0.201	-12.17519	2.555893
_cons	6.6	2.590077	2.55	0.011	1.523542	11.67646
_y_E						
des_AE	-.4242197	2.810983	-0.15	0.880	-5.933644	5.085205
des_BE	.398245	3.221872	0.12	0.902	-5.916508	6.712998
_cons	2.104	2.558451	0.82	0.411	-2.910472	7.118472
_y_F						
des_BF	5.099325	1.726678	2.95	0.003	1.715099	8.483551
des_CF	2.129756	2.815275	0.76	0.449	-3.388082	7.647594
des_FG	-2.777251	2.450728	-1.13	0.257	-7.58059	2.026087
_cons	7.327771	.8861815	8.27	0.000	5.590887	9.064654
_y_G						
des_AG	1.275388	1.880271	0.68	0.498	-2.409874	4.960651
des_BG	4.235611	3.010028	1.41	0.159	-1.663936	10.13516
_cons	.2537345	1.502189	0.17	0.866	-2.690502	3.197971
_y_H						
des_BH	.3596695	3.191427	0.11	0.910	-5.895413	6.614752
_cons	6.929682	1.274097	5.44	0.000	4.432498	9.426867
_y_I						
des_BI	1.404938	3.31673	0.42	0.672	-5.095734	7.90561
_cons	9.287914	1.461523	6.35	0.000	6.423381	12.15245
_y_J						
_cons	.1934848	2.25647	0.09	0.932	-4.229115	4.616084

Loop Inconsistency Model

A: Placebo; B: bisphosphonate (BP); C: monoclonal antibody (mAb); D: hormone replacement therapy (HRT); E: selective estrogen receptor modulator (SERM); F: parathyroid hormone (PTH); G: Calcitonin; H: strontium ranelate (SrRan); I: Fluoride; J: active vitamin D (VitD)

```
Testing for inconsistency:
 ( 1)  [_y_D]groupB = 0
 ( 2)  [_y_E]groupB = 0
 ( 3)  [_y_F]groupB = 0
 ( 4)  [_y_G]groupB = 0
 ( 5)  [_y_H]groupB = 0
 ( 6)  [_y_I]groupB = 0
 ( 7)  [_y_F]groupC = 0
 ( 8)  [_y_G]groupF = 0

           chi2(  8) =   13.32
         Prob > chi2 =    0.1013
```

	Coef.	Std. Err.	z	P>|z|	[95% Conf.	Interval]
_y_B						
_cons	6.160807	.4423097	13.93	0.000	5.293896	7.027718
_y_C						
_cons	8.258042	1.721215	4.80	0.000	4.884523	11.63156
_y_D						
groupB	-3.163401	2.969698	-1.07	0.287	-8.983903	2.657101
_cons	4.725198	1.390939	3.40	0.001	1.999008	7.451387
_y_E						
groupB	.3130226	2.130841	0.15	0.883	-3.863349	4.489394
_cons	1.969962	1.002213	1.97	0.049	.0056604	3.934264
_y_F						
groupB	4.84199	1.665609	2.91	0.004	1.577456	8.106524
groupC	2.126076	2.737019	0.78	0.437	-3.238383	7.490536
_cons	7.331952	.8611177	8.51	0.000	5.644192	9.019711
_y_G						
groupB	3.028913	2.637923	1.15	0.251	-2.141321	8.199148
groupF	1.799142	2.064046	0.87	0.383	-2.246314	5.844598
_cons	1.23188	.8312695	1.48	0.138	-.3973784	2.861138
_y_H						
groupB	.1272365	3.104391	0.04	0.967	-5.957258	6.211731
_cons	6.933562	1.235132	5.61	0.000	4.512747	9.354377
_y_I						
groupB	1.209236	3.251925	0.37	0.710	-5.16442	7.582892
_cons	9.26654	1.42258	6.51	0.000	6.478334	12.05475
_y_J						
_cons	-.0529302	2.197001	-0.02	0.981	-4.358973	4.253112

Side-Splitting Model

A: Placebo; B: bisphosphonate (BP); C: monoclonal antibody (mAb); D: hormone replacement therapy (HRT); E: selective estrogen receptor modulator (SERM); F: parathyroid hormone (PTH); G: Calcitonin; H: strontium ranelate (SrRan); I: Fluoride; J: active vitamin D (VitD)

Side	Direct		Indirect		Difference			tau
	Coef.	Std. Err.	Coef.	Std. Err.	Coef.	Std. Err.	P>|z|	
A B	6.196154	.4538203	4.132096	.9570798	2.064058	1.059516	0.051	2.455163
A C	8.257558	1.778173	7.045155	2.117893	1.212403	2.765392	0.661	2.511303
A D	4.606274	1.504086	2.129696	2.35353	2.476578	2.795795	0.376	2.49907
A E	1.750899	1.061037	2.459429	1.812532	-.7085292	2.099402	0.736	2.512755
A F	7.330105	.8719392	9.773941	1.054239	-2.443836	1.368277	0.074	2.46484
A G	1.065313	.8890846	3.778371	1.340119	-2.713057	1.610263	0.092	2.468347
A H	6.929513	1.275878	6.713392	2.916039	.2161202	3.182948	0.946	2.513355
A I	9.286751	1.459207	10.13511	2.96143	-.8483575	3.302089	0.797	2.505472
B D	-2.217559	1.864503	-1.667899	1.78944	-.5496591	2.586568	0.832	2.514939
B E	-3.294734	1.524342	-4.26139	1.215859	.9666563	1.949831	0.620	2.510031
B F	5.998816	1.332838	1.130306	.834634	4.86851	1.573804	0.002	2.386217
B G	-4.03145	1.29177	-3.846867	1.020269	-.1845824	1.64637	0.911	2.516907
B H	.9000007	2.88527	1.11611	1.344023	-.216109	3.182951	0.946	2.513357
B I	4.304115	2.931347	3.45575	1.518741	.8483655	3.302089	0.797	2.505472
B J *	-6.198922	2.190618	-11.57971	3896.614	5.380786	3896.614	0.999	2.495973
C F	1.200002	1.996633	-.0123734	1.913337	1.212375	2.765393	0.661	2.511304
F G	-4.298237	1.707496	-7.263246	1.070888	2.965008	2.015653	0.141	2.481604

(B) BMD at total hip (TH)

Design-by-Treatment Interaction Model

A: Placebo; B: bisphosphonate (BP); C: monoclonal antibody (mAb); D: hormone replacement therapy (HRT); E: selective estrogen receptor modulator (SERM); F: parathyroid hormone (PTH); G: Calcitonin; H: strontium ranelate (SrRan); I: Fluoride; J: active vitamin D (VitD)

```
Testing for inconsistency:
 ( 1)  [_y_B]des_ABD = 0
 ( 2)  [_y_B]des_ABE = 0
 ( 3)  [_y_B]des_ABG = 0
 ( 4)  [_y_D]des_AD = 0
 ( 5)  [_y_E]des_AE = 0
 ( 6)  [_y_G]des_AG = 0
 ( 7)  [_y_D]des_BD = 0
 ( 8)  [_y_E]des_BE = 0
 ( 9)  [_y_F]des_BF = 0
 (10)  [_y_G]des_BG = 0
 (11)  [_y_H]des_BH = 0
 (12)  [_y_I]des_BI = 0
 (13)  [_y_F]des_CF = 0
 (14)  [_y_F]des_FG = 0

           chi2( 14) =   11.48
         Prob > chi2 =    0.6480
```

	Coef.	Std. Err.	z	P>|z|	[95% Conf.	Interval]
_y_B						
des_ABD	-.0690897	1.95869	-0.04	0.972	-3.908051	3.769872
des_ABE	-1.26909	1.976752	-0.64	0.521	-5.143452	2.605272
des_ABG	-.2143737	1.190988	-0.18	0.857	-2.548668	2.119921
_cons	3.76909	.4070995	9.26	0.000	2.971189	4.56699
_y_C						
_cons	6.799945	1.830474	3.71	0.000	3.212281	10.38761
_y_D						
des_AD	1.5	2.66051	0.56	0.573	-3.714503	6.714503
des_BD	.8590806	2.770526	0.31	0.757	-4.571051	6.289212
_cons	3.1	1.915944	1.62	0.106	-.655182	6.855182
_y_E						
des_AE	.1978133	2.338011	0.08	0.933	-4.384605	4.780231
des_BE	1.269086	2.706993	0.47	0.639	-4.036522	6.574695
_cons	1.5	1.910786	0.79	0.432	-2.245071	5.245071
_y_F						
des_BF	3.496893	1.272521	2.75	0.006	1.002797	5.990989
des_CF	4.205445	2.369122	1.78	0.076	-.4379488	8.848838
des_FG	-.5267585	2.246476	-0.23	0.815	-4.92977	3.876253
_cons	1.394485	.6547347	2.13	0.033	.111229	2.677742
_y_G						
des_AG	.4071333	1.540162	0.26	0.792	-2.611529	3.425796
des_BG	1.701332	2.218675	0.77	0.443	-2.647191	6.049856
_cons	.9677511	1.116166	0.87	0.386	-1.219894	3.155396
_y_H						
des_BH	-.6699296	2.422117	-0.28	0.782	-5.417192	4.077333
_cons	5.839013	1.077182	5.42	0.000	3.727775	7.950252
_y_I						
des_BI	-.2202793	1.956448	-0.11	0.910	-4.054847	3.614288
_cons	1.767048	.8704673	2.03	0.042	.0609633	3.473132
_y_J						
_cons	1.569086	2.198547	0.71	0.475	-2.739987	5.87816

Loop Inconsistency Model

A: Placebo; B: bisphosphonate (BP); C: monoclonal antibody (mAb); D: hormone replacement therapy (HRT); E: selective estrogen receptor modulator (SERM); F: parathyroid hormone (PTH); G: Calcitonin; H: strontium ranelate (SrRan); I: Fluoride; J: active vitamin D (VitD)

```
Testing for inconsistency:
 ( 1)  [_y_D]groupB = 0
 ( 2)  [_y_E]groupB = 0
 ( 3)  [_y_F]groupB = 0
 ( 4)  [_y_G]groupB = 0
 ( 5)  [_y_H]groupB = 0
 ( 6)  [_y_I]groupB = 0
 ( 7)  [_y_F]groupC = 0
 ( 8)  [_y_G]groupF = 0

           chi2(  8) =   11.63
         Prob > chi2 =    0.1685
```

	Coef.	Std. Err.	z	P>|z|	[95% Conf.	Interval]
_y_B						
_cons	3.717969	.3433816	10.83	0.000	3.044953	4.390984
_y_C						
_cons	6.799951	1.719434	3.95	0.000	3.429923	10.16998
_y_D						
groupB	.1414085	2.198276	0.06	0.949	-4.167132	4.449949
_cons	3.766552	1.156964	3.26	0.001	1.498944	6.03416
_y_E						
groupB	.8636165	2.042042	0.42	0.672	-3.138711	4.865944
_cons	1.854349	.9902935	1.87	0.061	-.0865906	3.795289
_y_F						
groupB	3.405061	1.193896	2.85	0.004	1.065068	5.745054
groupC	4.194764	2.230827	1.88	0.060	-.1775774	8.567105
_cons	1.405175	.6199413	2.27	0.023	.1901119	2.620237
_y_G						
groupB	1.419738	1.90636	0.74	0.456	-2.316659	5.156134
groupF	.3069156	1.955892	0.16	0.875	-3.526561	4.140393
_cons	1.198225	.6805293	1.76	0.078	-.1355877	2.532038
_y_H						
groupB	-.7318574	2.300579	-0.32	0.750	-5.240909	3.777194
_cons	5.849821	1.014107	5.77	0.000	3.862208	7.837433
_y_I						
groupB	-.2829641	1.871088	-0.15	0.880	-3.950229	3.384301
_cons	1.758951	.823268	2.14	0.033	.1453759	3.372527
_y_J						
_cons	1.517965	2.095615	0.72	0.469	-2.589364	5.625295

Side-Splitting Model

A: Placebo; B: bisphosphonate (BP); C: monoclonal antibody (mAb); D: hormone replacement therapy (HRT); E: selective estrogen receptor modulator (SERM); F: parathyroid hormone (PTH); G: Calcitonin; H: strontium ranelate (SrRan); I: Fluoride; J: active vitamin D (VitD)

Side	Direct Coef.	Std. Err.	Indirect Coef.	Std. Err.	Difference Coef.	Std. Err.	P>|z|	tau
A B	3.696167	.3519623	2.440064	.7261904	1.256102	.8070284	0.120	1.733721
A C	6.8	1.726267	3.441772	1.381626	3.358228	2.211083	0.129	1.720702
A D	3.873681	1.286719	3.342586	1.682254	.531095	2.127133	0.803	1.76342
A E	1.632294	1.064462	2.72948	1.632778	-1.097186	1.949113	0.573	1.759134
A F	1.410056	.6057899	4.211569	.8077713	-2.801513	1.010673	0.006	1.668444
A G	1.183228	.7425085	1.990229	1.123091	-.8070009	1.34618	0.549	1.758431
A H	5.84525	1.039477	4.837181	2.099256	1.008068	2.342481	0.667	1.758721
A I	1.762276	.8418495	1.206052	1.699594	.5562234	1.896449	0.769	1.757798
B D	-.2238181	1.310143	.8934242	1.611651	-1.117242	2.076984	0.591	1.759811
B E	-1.004332	1.301361	-1.961611	1.263921	.9572786	1.817276	0.598	1.759662
B F	1.08132	.9417747	-2.005059	.6356444	3.086379	1.137343	0.007	1.675916
B G	-2.195934	.932067	-1.872853	.8948605	-.3230808	1.29269	0.803	1.764301
B H	1.4	2.074132	2.408064	1.088665	-1.008064	2.34248	0.667	1.75872
B I	-2.23404	1.668257	-1.677821	.9026208	-.5562187	1.896451	0.769	1.757801
B J *	-2.2	2.094411	-6.842195	2873.939	4.642195	2873.94	0.999	1.746469
C F	-1.2	1.283596	-4.558203	1.80035	3.358203	2.21108	0.129	1.720699
F G	.1	1.769043	-1.23194	.8266983	1.33194	1.952676	0.495	1.757447

(C) BMD at radius (RU)

Design-by-Treatment Interaction Model

A: Placebo; B: bisphosphonate (BP); C: monoclonal antibody (mAb); D: hormone replacement therapy (HRT); E: selective estrogen receptor modulator (SERM); F: parathyroid hormone (PTH); G: Calcitonin; H: Fluoride; I: active vitamin D (VitD); J: vitamin K (VitK)

```
Testing for inconsistency:
 ( 1)  [_y_B]des_ABDGIJ = 0
 ( 2)  [_y_B]des_ABJ = 0
 ( 3)  [_y_J]des_ABJ = 0
 ( 4)  [_y_F]des_BF = 0

           chi2(  4) =    2.97
         Prob > chi2 =    0.5621
```

	Coef.	Std. Err.	z	P>|z|	[95% Conf.	Interval]
_y_B						
des_ABDGIJ	1.031458	2.250362	0.46	0.647	-3.379171	5.442087
des_ABJ	1.031458	2.438394	0.42	0.672	-3.747706	5.810622
_cons	1.768542	1.029362	1.72	0.086	-.248971	3.786055
_y_C						
_cons	2.468227	2.138485	1.15	0.248	-1.723127	6.659581
_y_D						
_cons	5.3	2.006328	2.64	0.008	1.36767	9.23233
_y_E						
_cons	.7	2.030781	0.34	0.730	-3.280257	4.680257
_y_F						
des_BF	-4.856083	2.887236	-1.68	0.093	-10.51496	.8027955
_cons	-1.175372	1.471968	-0.80	0.425	-4.060377	1.709632
_y_G						
_cons	4.9	1.996657	2.45	0.014	.9866234	8.813377
_y_H						
_cons	1.861939	1.519276	1.23	0.220	-1.115787	4.839664
_y_I						
_cons	-.3	1.996472	-0.15	0.881	-4.213013	3.613013
_y_J						
des_ABJ	-.8	2.987729	-0.27	0.789	-6.655841	5.055841
_cons	1.4	2.009785	0.70	0.486	-2.539105	5.339105

Loop Inconsistency Model

A: Placebo; B: bisphosphonate (BP); C: monoclonal antibody (mAb); D: hormone replacement therapy (HRT); E: selective estrogen receptor modulator (SERM); F: parathyroid hormone (PTH); G: Calcitonin; H: Fluoride; I: active vitamin D (VitD); J: vitamin K (VitK)

```
Testing for inconsistency:
 ( 1)  [_y_F]groupB = 0

           chi2(  1) =    3.39
         Prob > chi2 =    0.0656
```

		Coef.	Std. Err.	z	P>|z|	[95% Conf. Interval]	
_y_B							
	_cons	2.113888	.7010388	3.02	0.003	.7398771	3.487899
_y_C							
	_cons	2.379368	1.8008	1.32	0.186	-1.150136	5.908871
_y_D							
	_cons	4.851498	1.43538	3.38	0.001	2.038206	7.664791
_y_E							
	_cons	.7	1.671868	0.42	0.675	-2.576801	3.976801
_y_F							
	groupB	-4.424314	2.402632	-1.84	0.066	-9.133386	.2847568
	_cons	-1.261796	1.225691	-1.03	0.303	-3.664107	1.140514
_y_G							
	_cons	4.451498	1.421832	3.13	0.002	1.664759	7.238237
_y_H							
	_cons	1.770134	1.280285	1.38	0.167	-.7391788	4.279446
_y_I							
	_cons	-.7485019	1.421571	-0.53	0.599	-3.534731	2.037727
_y_J							
	_cons	.7315285	1.139759	0.64	0.521	-1.502358	2.965415

Side-Splitting Model

A: Placebo; B: bisphosphonate (BP); C: monoclonal antibody (mAb); D: hormone replacement therapy (HRT); E: selective estrogen receptor modulator (SERM); F: parathyroid hormone (PTH); G: Calcitonin; H: Fluoride; I: active vitamin D (VitD); J: vitamin K (VitK)

Side	Direct		Indirect		Difference			tau
	Coef.	Std. Err.	Coef.	Std. Err.	Coef.	Std. Err.	P>|z|	
A B *	2.113887	.7010388	6.538194	2.296828	-4.424307	2.402629	0.066	1.599093
A D *	5.3	1.946608	4.233402	3.401374	1.066598	3.911008	0.785	1.910487
A E	.	.	.	.	.	.	.	.
A F	-1.261795	1.225691	-5.686086	2.065077	4.424291	2.402625	0.066	1.599094
A G *	4.9	1.93664	3.833402	3.39568	1.066598	3.911009	0.785	1.910488
A H	.	.	.	.	.	.	.	.
A I *	-.3	1.936449	-1.366598	3.395571	1.066598	3.911009	0.785	1.910488
A J *	1.043364	1.442688	.2259352	3.132171	.8174286	3.428409	0.812	1.904758
B D *	2.5	1.959608	2.633439	3.431117	-.1334387	3.946012	0.973	1.920996
B F	-7.8	1.942451	-3.375684	1.414044	-4.424316	2.402631	0.066	1.599093
B G *	2.1	1.949707	2.233438	3.425472	-.1334383	3.946014	0.973	1.920997
B I *	-3.1	1.949514	-2.966561	3.425359	-.1334391	3.946008	0.973	1.920993
B J *	-1.749244	1.462868	-.9318149	3.104718	-.8174287	3.428417	0.812	1.904763
C F *	-3.642489	1.431095	-4.742603	1614.783	1.100114	1614.784	0.999	1.781107
D G	.	.	.	.	.	.	.	.
D I	.	.	.	.	.	.	.	.
D J *	-3.9	1.942151	-5.482926	4.667951	1.582926	5.058605	0.754	1.894049
G I	.	.	.	.	.	.	.	.
G J *	-3.5	1.932156	-5.082925	4.663797	1.582925	5.058598	0.754	1.894046
I J *	1.7	1.931966	.1170741	4.66372	1.582926	5.058601	0.754	1.894047

(D) Incidence of Cancer

Design-by-Treatment Interaction Model

A: Placebo; B: bisphosphonate (BP); C: monoclonal antibody (mAb); D: selective estrogen receptor modulator (SERM)

```
Testing for inconsistency:
 ( 1)  [_y_C]des_BC = 0

           chi2(  1) =    0.15
         Prob > chi2 =    0.6991
```

	Coef.	Std. Err.	z	P>|z|	[95% Conf. Interval]	
_y_B						
_cons	.1163682	.3669585	0.32	0.751	-.6028573	.8355938
_y_C						
des_BC	.1904792	.4928562	0.39	0.699	-.7755012	1.15646
_cons	.0161595	.1456462	0.11	0.912	-.2693019	.3016209
_y_D						
_cons	-.4320222	.1865551	-2.32	0.021	-.7976635	-.0663809

Loop Inconsistency Model

A: Placebo; B: bisphosphonate (BP); C: monoclonal antibody (mAb); D: selective estrogen receptor modulator (SERM)

```
Testing for inconsistency:
 ( 1)  [_y_C]groupB = 0

           chi2(  1) =    0.15
         Prob > chi2 =    0.6991
```

	Coef.	Std. Err.	z	P>|z|	[95% Conf. Interval]	
_y_B						
_cons	.1163682	.3669585	0.32	0.751	-.6028573	.8355938
_y_C						
groupB	.1904792	.4928562	0.39	0.699	-.7755012	1.15646
_cons	.0161595	.1456462	0.11	0.912	-.2693019	.3016209
_y_D						
_cons	-.4320222	.1865551	-2.32	0.021	-.7976635	-.0663809

Side-Splitting Model

A: Placebo; B: bisphosphonate (BP); C: monoclonal antibody (mAb); D: selective estrogen receptor modulator (SERM)

Side	Direct Coef.	Std. Err.	Indirect Coef.	Std. Err.	Difference Coef.	Std. Err.	P>|z|	tau
A B	.116367	.3669618	-.0741228	.3291707	.1904898	.4929648	0.699	.1344039
A C	.0161461	.1450142	.2066352	.4708476	-.1904891	.4926729	0.699	.1344012
A D	.	.	.	.	.	.	.	.
B C	.0902683	.2950202	-.1002212	.3947944	.1904894	.4928484	0.699	.1343997

(E) Incidence of hip fracture

Design-by-Treatment Interaction Model

A: Placebo; B: bisphosphonate (BP); C: monoclonal antibody (mAb); D: selective estrogen receptor modulator (SERM); E: parathyroid hormone (PTH); F: Calcitonin; G: Fluoride

```
Testing for inconsistency:
 ( 1)  [_y_E]des_BE = 0

           chi2(  1) =    0.73
         Prob > chi2 =    0.3934
```

		Coef.	Std. Err.	z	P>|z|	[95% Conf.	Interval]
_y_B							
	_cons	-.560394	.1743985	-3.21	0.001	-.9022089	-.2185792
_y_C							
	_cons	-.5064632	.2494679	-2.03	0.042	-.9954112	-.0175152
_y_D							
	_cons	-.0491759	.2307689	-0.21	0.831	-.5014746	.4031228
_y_E							
	des_BE	-.9368668	1.097788	-0.85	0.393	-3.088491	1.214757
	_cons	-.5442526	.6868526	-0.79	0.428	-1.890459	.8019537
_y_F							
	_cons	-.2845401	.5105043	-0.56	0.577	-1.28511	.7160299
_y_G							
	_cons	-.2914165	.6460761	-0.45	0.652	-1.557702	.9748694

Loop Inconsistency Model

A: Placebo; B: bisphosphonate (BP); C: monoclonal antibody (mAb); D: selective estrogen receptor modulator (SERM); E: parathyroid hormone (PTH); F: Calcitonin; G: Fluoride

```
Testing for inconsistency:
 ( 1)  [_y_E]groupB = 0

           chi2(  1) =    0.73
         Prob > chi2 =    0.3934
```

		Coef.	Std. Err.	z	P>|z|	[95% Conf. Interval]	
_y_B							
	_cons	-.560394	.1743985	-3.21	0.001	-.9022089	-.2185792
_y_C							
	_cons	-.5064632	.2494679	-2.03	0.042	-.9954112	-.0175152
_y_D							
	_cons	-.0491759	.2307689	-0.21	0.831	-.5014746	.4031228
_y_E							
	groupB	-.9368668	1.097788	-0.85	0.393	-3.088491	1.214757
	_cons	-.5442526	.6868526	-0.79	0.428	-1.890459	.8019537
_y_F							
	_cons	-.2845401	.5105043	-0.56	0.577	-1.28511	.7160299
_y_G							
	_cons	-.2914165	.6460761	-0.45	0.652	-1.557702	.9748694

Side-Splitting Model

A: Placebo; B: bisphosphonate (BP); C: monoclonal antibody (mAb); D: selective estrogen receptor modulator (SERM); E: parathyroid hormone (PTH); F: Calcitonin; G: Fluoride

Side	Direct		Indirect		Difference			tau
	Coef.	Std. Err.	Coef.	Std. Err.	Coef.	Std. Err.	P>|z|	
A B	-.560394	.1743985	.3764728	1.083847	-.9368667	1.097788	0.393	2.47e-06
A C	.	.	.	.	.	.	.	.
A D	.	.	.	.	.	.	.	.
A E	-.5442526	.6868526	-1.481119	.856371	.9368668	1.097788	0.393	2.57e-06
A F	.	.	.	.	.	.	.	.
A G	.	.	.	.	.	.	.	.
B E	-.9207253	.8384249	.0161415	.7086475	-.9368668	1.097788	0.393	3.05e-08

(F) Incidence of Death

Design-by-Treatment Interaction Model

A: Placebo; B: bisphosphonate (BP); C: monoclonal antibody (mAb); D: selective estrogen receptor modulator (SERM); E: parathyroid hormone (PTH)

```
Testing for inconsistency:
 ( 1)  [_y_E]des_BE = 0

           chi2(  1) =    1.91
         Prob > chi2 =    0.1673
```

		Coef.	Std. Err.	z	P>|z|	[95% Conf. Interval]	
_y_B							
	_cons	-.0024594	.1887356	-0.01	0.990	-.3723744	.3674556
_y_C							
	_cons	-.2303769	.2040826	-1.13	0.259	-.6303714	.1696177
_y_D							
	_cons	.1724264	.1717063	1.00	0.315	-.1641118	.5089646
_y_E							
	des_BE	1.282465	.928733	1.38	0.167	-.5378186	3.102748
	_cons	-.5108256	.7583527	-0.67	0.501	-1.99717	.9755184

Loop Inconsistency Model

A: Placebo; B: bisphosphonate (BP); C: monoclonal antibody (mAb); D: selective estrogen receptor modulator (SERM); E: parathyroid hormone (PTH)

```
Testing for inconsistency:
 ( 1)  [_y_E]groupB = 0

           chi2(  1) =    1.91
         Prob > chi2 =    0.1673
```

	Coef.	Std. Err.	z	P>|z|	[95% Conf.	Interval]
_y_B						
_cons	-.0024594	.1887356	-0.01	0.990	-.3723744	.3674556
_y_C						
_cons	-.2303769	.2040826	-1.13	0.259	-.6303714	.1696177
_y_D						
_cons	.1724264	.1717063	1.00	0.315	-.1641118	.5089646
_y_E						
groupB	1.282465	.928733	1.38	0.167	-.5378186	3.102748
_cons	-.5108256	.7583527	-0.67	0.501	-1.99717	.9755184

Side-Splitting Model

A: Placebo; B: bisphosphonate (BP); C: monoclonal antibody (mAb); D: selective estrogen receptor modulator (SERM); E: parathyroid hormone (PTH)

Side	Direct		Indirect		Difference			tau
	Coef.	Std. Err.	Coef.	Std. Err.	Coef.	Std. Err.	P>|z|	
A B	-.0024594	.1887395	-1.284924	.9093537	1.282464	.928734	0.167	.1982714
A C	.	.	.	.	.	.	.	.
A D	.	.	.	.	.	.	.	.
A E	-.5108256	.7583526	.771639	.5361399	-1.282465	.9287328	0.167	.1982711
B E	.7740984	.5018233	-.5083651	.7814814	1.282463	.9287302	0.167	.1982754

Figure S6. Comparison-Adjusted Funnel Plots for the Outcomes of BMD at **(A)** lumbar spine (LS) **(B)** total hip (TH) **(C)** radius (RU) and incidence of **(D)** cancer **(E)** cardiovascular disease (CVD) **(F)** hip fracture **(G)** death. The red line represents the null hypothesis that the study-specific effect sizes do not differ from the respective comparison-specific pooled effect estimates.

(A) BMD at lumbar spine (LS)

A: Placebo; B: bisphosphonate (BP); C: monoclonal antibody (mAb); D: hormone replacement therapy (HRT); E: selective estrogen receptor modulator (SERM); F: parathyroid hormone (PTH); G: Calcitonin; H: strontium ranelate (SrRan); I: Fluoride; J: active vitamin D (VitD)

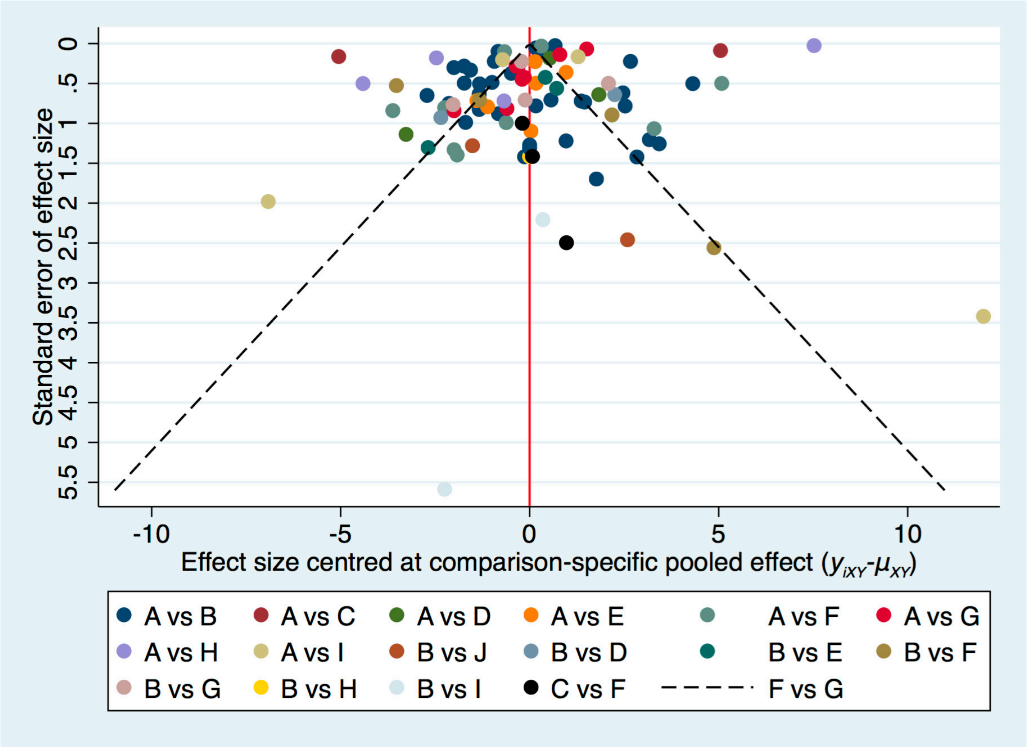

(B) BMD at total hip (TH)

A: Placebo; B: bisphosphonate (BP); C: monoclonal antibody (mAb); D: hormone replacement therapy (HRT); E: selective estrogen receptor modulator (SERM); F: parathyroid hormone (PTH); G: Calcitonin; H: strontium ranelate (SrRan); I: Fluoride; J: active vitamin D (VitD)

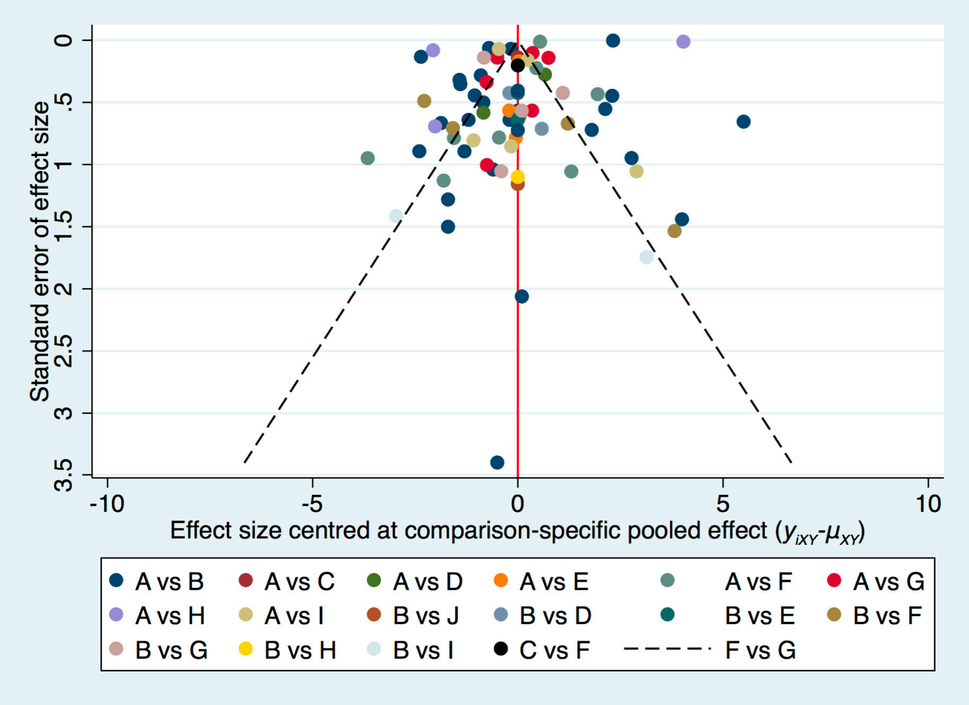

(C) BMD at radius (RU)

A: Placebo; B: bisphosphonate (BP); C: monoclonal antibody (mAb); D: hormone replacement therapy (HRT); E: selective estrogen receptor modulator (SERM); F: parathyroid hormone (PTH); G: Calcitonin; H: Fluoride; I: active vitamin D (VitD); J: vitamin K (VitK)

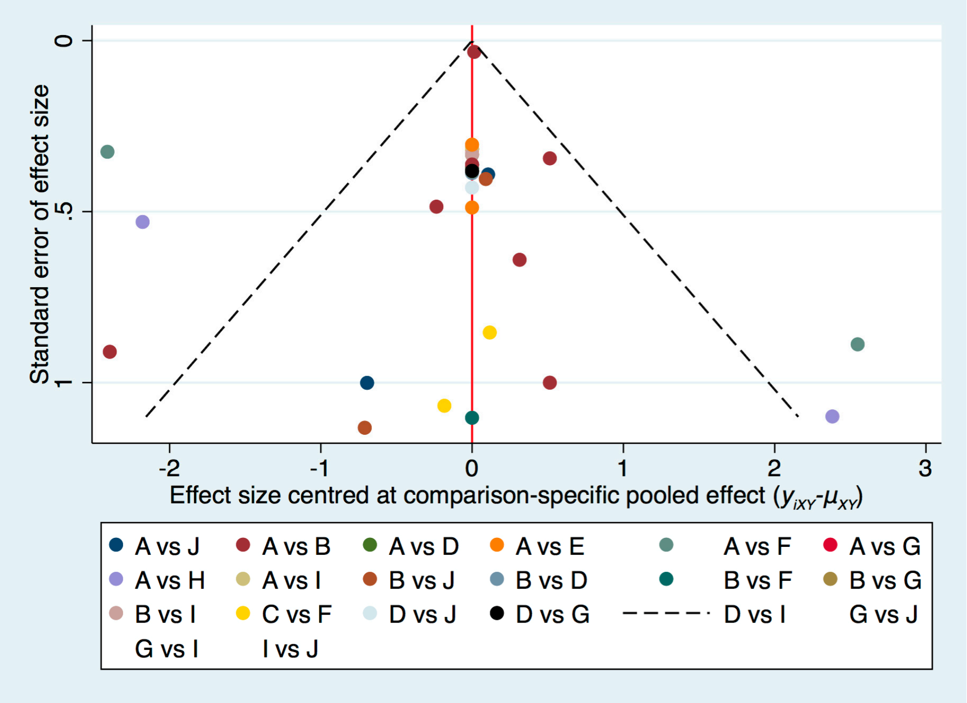

(D) Incidence of Cancer

A: Placebo; B: bisphosphonate (BP); C: monoclonal antibody (mAb); D: selective estrogen receptor modulator (SERM)

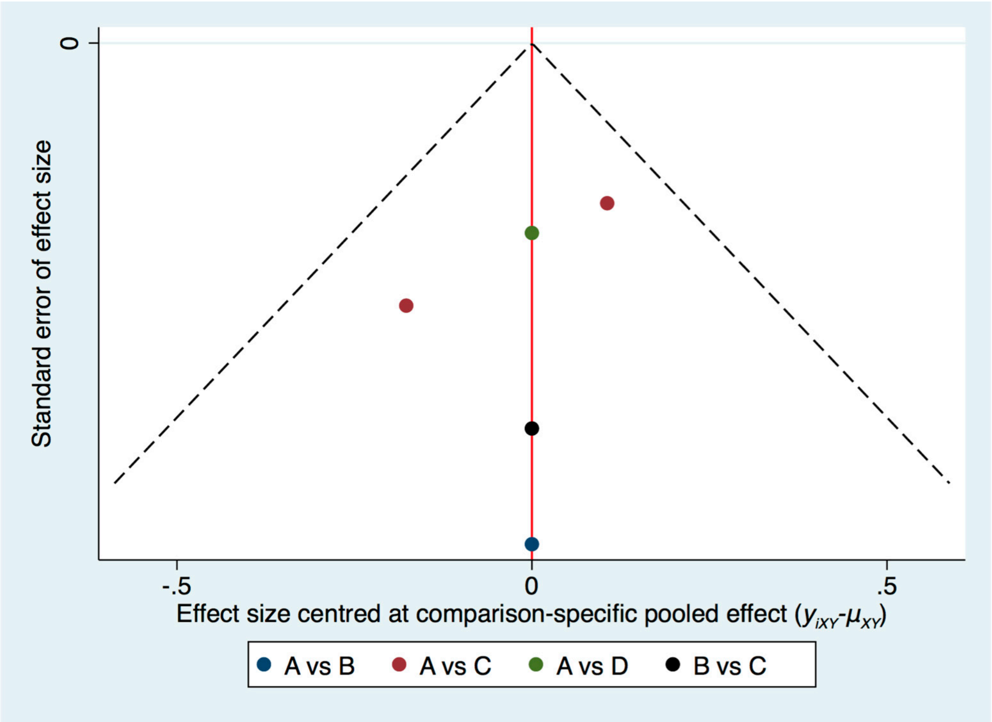

(E) Incidence of cardiovascular disease (CVD)

A: Placebo; B: bisphosphonate (BP); C: monoclonal antibody (mAb); D: selective estrogen receptor modulator (SERM)

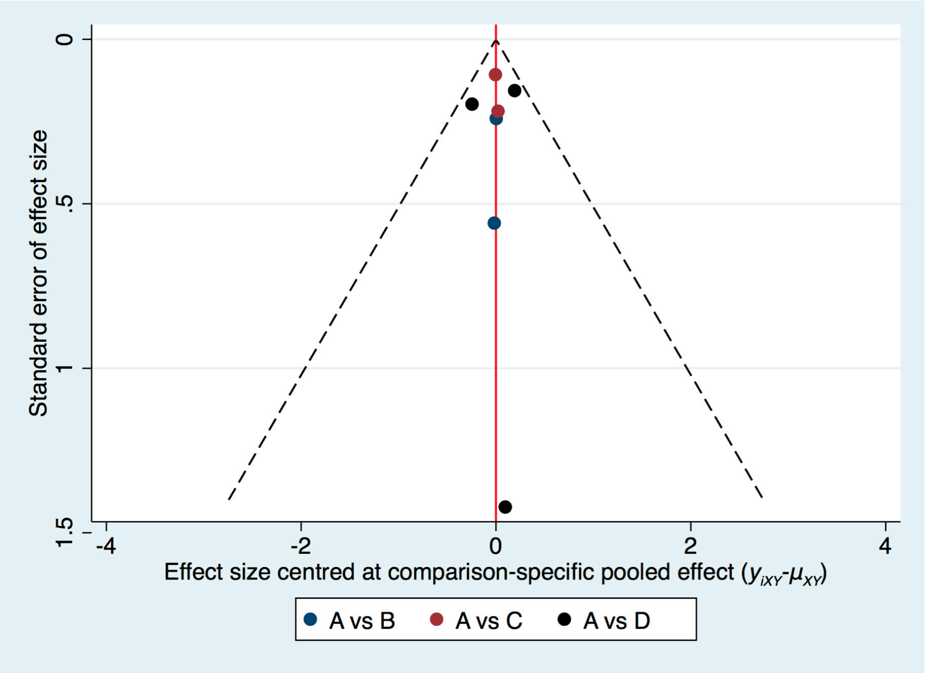

(F) Incidence of hip fracture

A: Placebo; B: bisphosphonate (BP); C: monoclonal antibody (mAb); D: selective estrogen receptor modulator (SERM); E: parathyroid hormone (PTH); F: Calcitonin; G: Fluoride

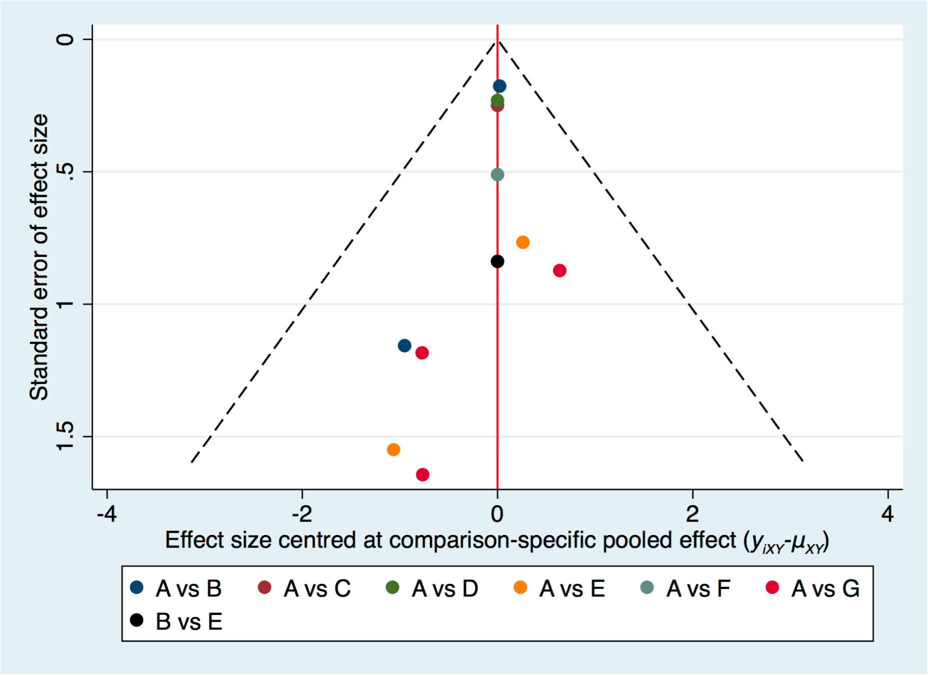

(G) Incidence of death

A: Placebo; B: bisphosphonate (BP); C: monoclonal antibody (mAb); D: selective estrogen receptor modulator (SERM); E: parathyroid hormone (PTH)

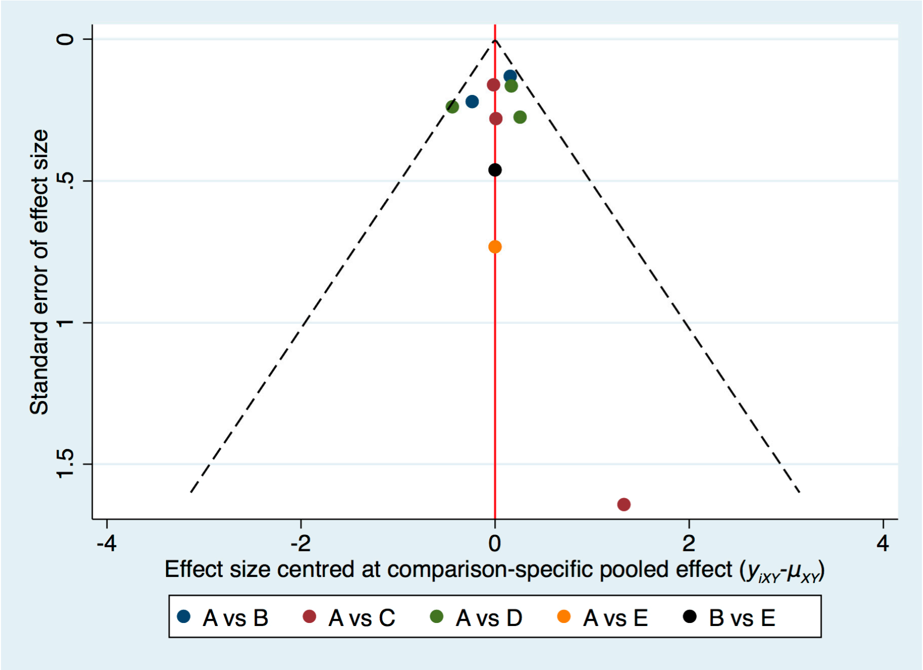

Figure S7. Egger's Publication Bias Plots for the Outcomes of BMD at **(A)** lumbar spine (LS) **(B)** total hip (TH) **(C)** radius (RU) and the incidence of **(D)** cancer **(E)** cardiovascular disease (CVD) **(F)** hip fracture **(G)** death The red diagonal line represents the regression line. The space between the red bars indicates the 95% confidence interval (CI) for the expected distribution of studies in the absence of heterogeneity between studies and absence of selection biases.

(A) BMD at lumbar spine (LS)

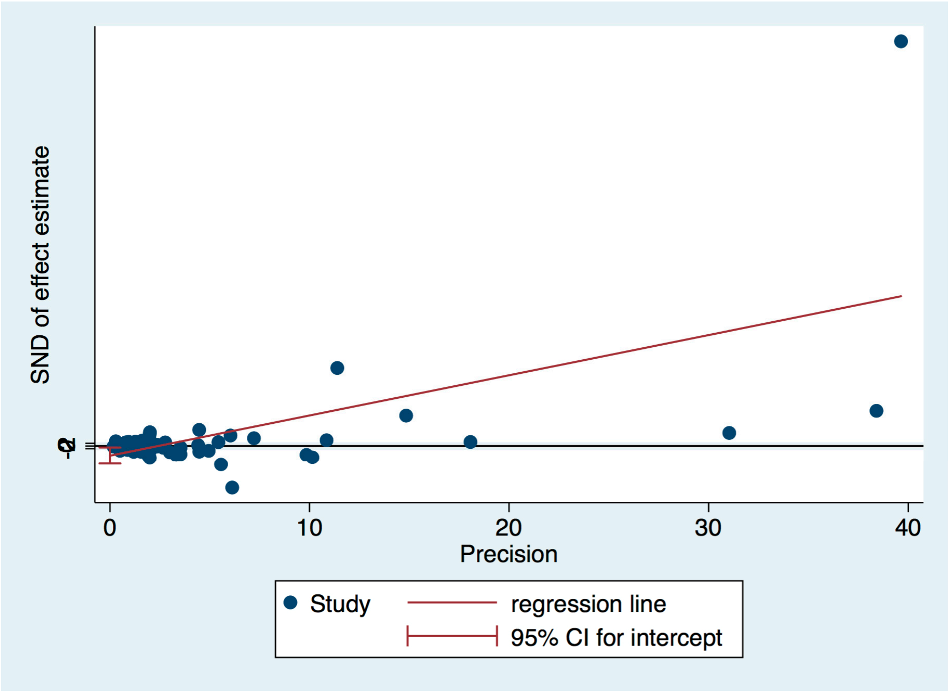


```
Egger's test for small-study effects:
Regress standard normal deviate of intervention
effect estimate against its standard error

Number of studies =   91                         Root MSE      =   25.36
```

Std_Eff	Coef.	Std. Err.	t	P>|t|	[95% Conf.	Interval]
slope	2.964489	.3904532	7.59	0.000	2.188667	3.740311
bias	-7.127783	3.030292	-2.35	0.021	-13.14891	-1.106658

```
Test of H0: no small-study effects          P = 0.021
```

(B) BMD at total hip (TH)

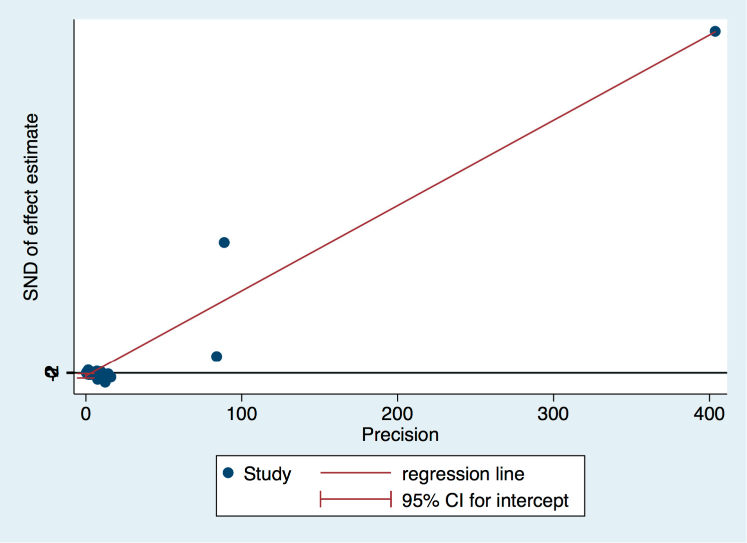

Egger's test for small-study effects:
Regress standard normal deviate of intervention
effect estimate against its standard error

Number of studies = 77 Root MSE = 26.97

Std_Eff	Coef.	Std. Err.	t	P>|t|	[95% Conf.	Interval]
slope	2.335164	.0652112	35.81	0.000	2.205257	2.465071
bias	-8.023912	3.148003	-2.55	0.013	-14.29505	-1.752769

Test of H0: no small-study effects P = 0.013

(C) BMD at radius (RU)

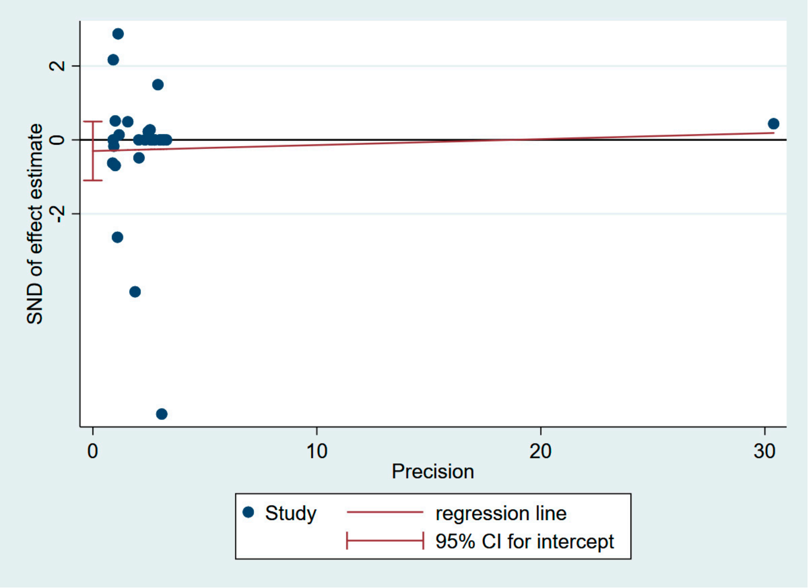

Egger's test for small-study effects:
Regress standard normal deviate of intervention
effect estimate against its standard error

Number of studies = 30 Root MSE = 1.831

Std_Eff	Coef.	Std. Err.	t	P>|t|	[95% Conf.	Interval]
slope	.0160382	.0650207	0.25	0.807	-.1171507	.1492272
bias	-.3001536	.3892059	-0.77	0.447	-1.097406	.4970984

Test of H0: no small-study effects P = 0.447

(D) Incidence of Cancer

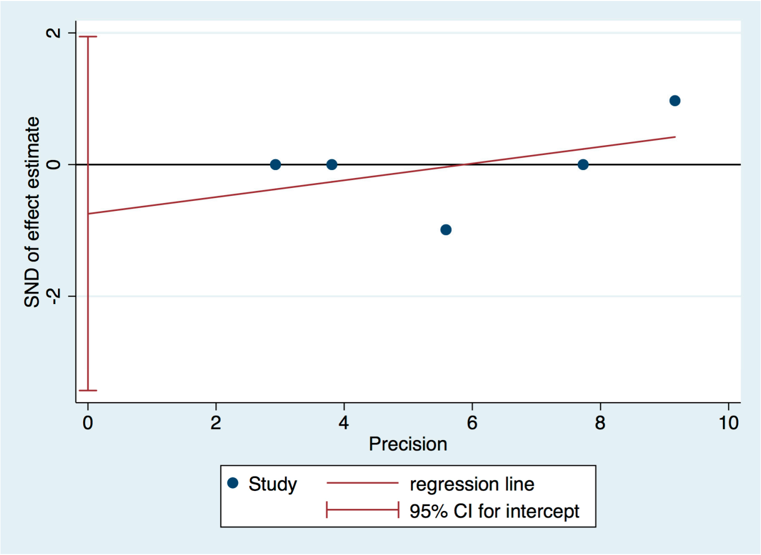

Egger's test for small-study effects:
Regress standard normal deviate of intervention
effect estimate against its standard error

Number of studies = 5 Root MSE = .702

Std_Eff	Coef.	Std. Err.	t	P>|t|	[95% Conf.	Interval]
slope	.127339	.1344146	0.95	0.413	-.3004284	.5551064
bias	-.7477861	.8458527	-0.88	0.442	-3.439667	1.944095

Test of H0: no small-study effects P = 0.442

(E) Incidence of cardiovascular disease (CVD)

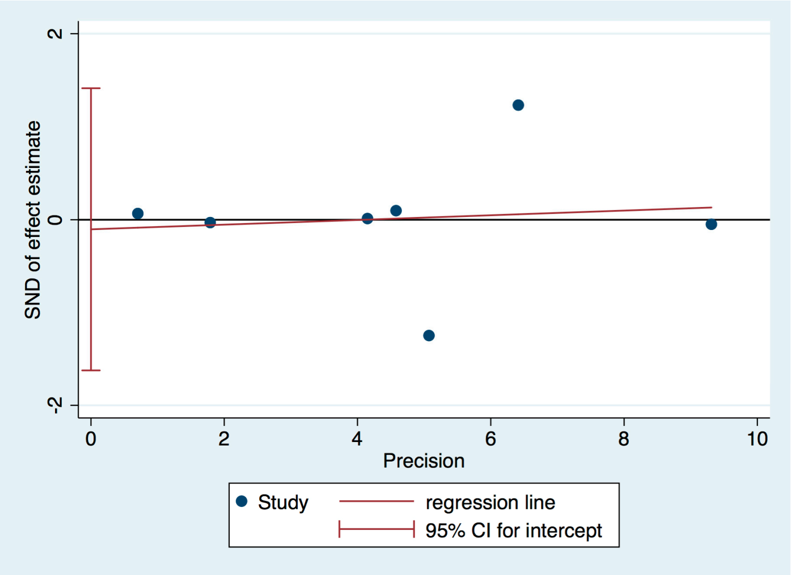

```
Egger's test for small-study effects:
Regress standard normal deviate of intervention
effect estimate against its standard error

Number of studies =   7                                        Root MSE      =    .7813
```

Std_Eff	Coef.	Std. Err.	t	P>|t|	[95% Conf.	Interval]
slope	.0252207	.1116302	0.23	0.830	-.2617339	.3121753
bias	-.1027587	.5898883	-0.17	0.869	-1.619115	1.413597

```
Test of H0: no small-study effects          P = 0.869
```

(F) Incidence of hip fracture

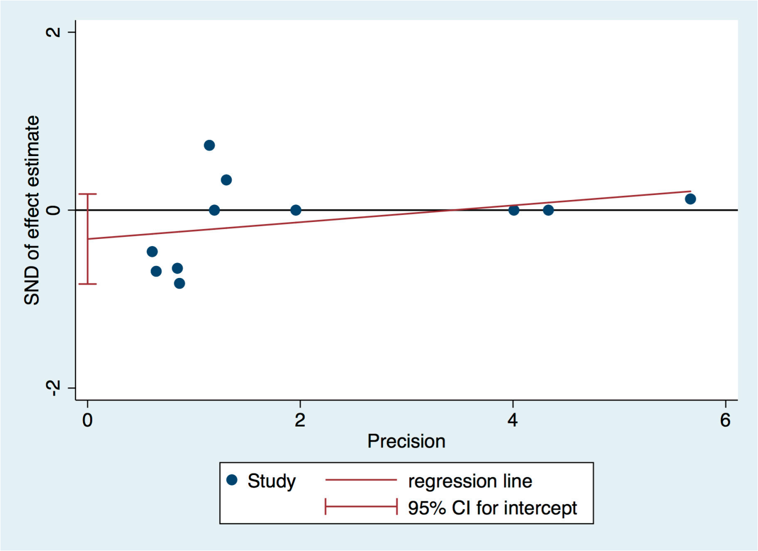


```
Egger's test for small-study effects:
Regress standard normal deviate of intervention
effect estimate against its standard error

Number of studies =   11                                       Root MSE      =    .4703
```

Std_Eff	Coef.	Std. Err.	t	P>|t|	[95% Conf.	Interval]
slope	.0945217	.0842401	1.12	0.291	-.0960427	.2850861
bias	-.3244908	.2236007	-1.45	0.181	-.8303107	.1813292

```
Test of H0: no small-study effects          P = 0.181
```

(G) Incidence of death

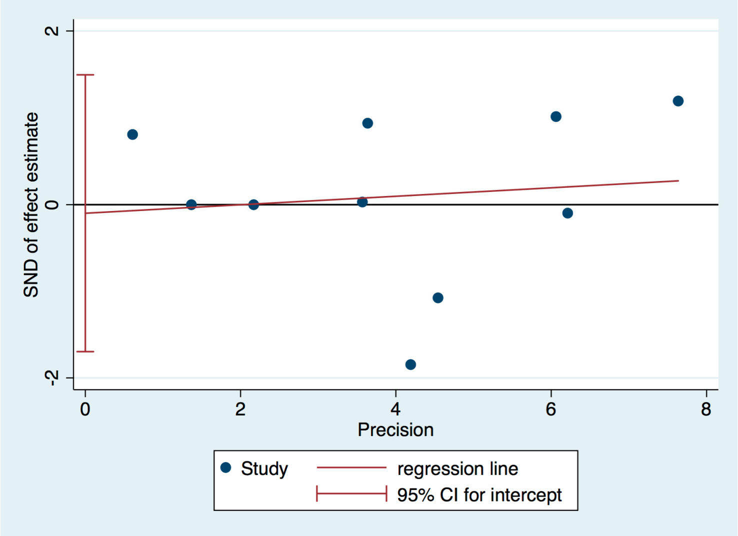


```
Egger's test for small-study effects:
Regress standard normal deviate of intervention
effect estimate against its standard error

Number of studies =   10                                Root MSE      =    1.022
```

Std_Eff	Coef.	Std. Err.	t	P>|t|	[95% Conf.	Interval]
slope	.0488426	.1527248	0.32	0.757	-.3033413	.4010265
bias	-.0983542	.6909909	-0.14	0.890	-1.691782	1.495074

```
Test of H0: no small-study effects          P = 0.890
```

References

1. Bonnick S, Broy S, Kaiser F, et al. Treatment with alendronate plus calcium, alendronate alone, or calcium alone for postmenopausal low bone mineral density. *Curr Med Res Opin.* 2007;23(6):1341-1349.
2. M Chen SNC. Additive effect of alfacalcidol on bone mineral density of the lumbar spine in Taiwanese postmenopausal women treated with hormone replacement therapy and calcium supplementation: a randomized 2-year study. *Clin Endocrinol (Oxf).* 2001;55(2):253-258.
3. Iseri K, Iyoda M, Watanabe M, et al. The effects of denosumab and alendronate on glucocorticoid-induced osteoporosis in patients with glomerular disease: A randomized, controlled trial. *PLoS One.* 2018;13(3):e0193846.
4. Alessandro Rubinacci EP, Alberto Bacchi Modena, Ettore Zanardi, Bruno Andrei, Vincenzo De Leo, Francesco Saverio Pansini, Erhard Quebe-Fehling, Patricia Ibarra de Palacios. Effect of low-dose transdermal E2/NETA on the reduction of postmenopausal bone loss in women. *Menopause.* 2003;10(3):241-249.
5. A Braga de Castro Machado R Hannon RE. Monitoring alendronate therapy for osteoporosis. *J Bone Miner Res.* 1999;14(4):602-608.
6. C Castelo-Branco JJV, F Figueras, A Sanjuan, M J Martínez de Osaba, E Casals, F Pons, J Balasch, J A Vanrell. Comparative effects of estrogens plus androgens and tibolone on bone, lipid pattern and sexuality in postmenopausal women. *Maturitas.* 2000;34(2):161-168.
7. Lucy Cooper PBC-B, M Liza Nery, Gemma Figtree, Stephen Twigg, Emily Hibbert, Bruce G Robinson. Vitamin D supplementation and bone mineral density in early postmenopausal women. *Am J Clin Nutr.* 2003;77(5):1324-1329.
8. B Dawson-Hughes GED, E A Krall, L Sadowski, N Sahyoun, S Tannenbaum. A controlled trial of the effect of calcium supplementation on bone density in postmenopausal women. *N Engl J Med.* 1990;323(13):878-883.
9. Grados F, Brazier M, Kamel S, et al. Prediction of bone mass density variation by bone remodeling markers in postmenopausal women with vitamin D insufficiency treated with calcium and vitamin D supplementation. *J Clin Endocrinol Metab.* 2003;88(11):5175-5179.
10. C J Haines TKC, P C Leung, S Y Hsu, D H Leung. Calcium supplementation and bone mineral density in postmenopausal women using estrogen replacement therapy. *Bone.* 1995;16(5):529-531.
11. S T Harris HKG, D J Baylink, J C Gallagher, S K Karp, M A McConnell, E M Green, R W Stoll. The effects of estrone (Ogen) on spinal bone density of postmenopausal women. *Arch Intern Med.* 1991;151(10):1980-1984.
12. T C Hillard SJW, M S Marsh, M C Ellerington, B Lees, M I Whitehead, J C Stevenson. Long-term effects of transdermal and oral hormone replacement therapy on postmenopausal bone loss. *Osteoporos Int.* 1994;4(6):341-348.
13. Mayuree Jirapinyo UT, Jittima Manonai, Charnchai Suchartwatnachai, Lars Nelleman Jorgensen. Effect of combined oral estrogen/progestogen preparation (Kliogest) on bone mineral density, plasma lipids and postmenopausal symptoms in HRT-naïve Thai women. *Acta Obstet Gynecol Scand.* 2003;82(9):857-866.

14. Fitzpatrick LA, Dabrowski CE, Cicconetti G, et al. The effects of ronacaleret, a calcium-sensing receptor antagonist, on bone mineral density and biochemical markers of bone turnover in postmenopausal women with low bone mineral density. *J Clin Endocrinol Metab.* 2011;96(8):2441-2449.
15. L You Z-YS, J-Y Chen, L Pan, L Chen. The safety and efficacy of early-stage bi-weekly alendronate to improve bone mineral density and bone turnover in chinese post-menopausal women at risk of osteoporosis. *J Int Med Res.* 2011;39(1):302-310.
16. Bhattoa HP, Bettembuk P, Balogh A, Szegedi G, Kiss E. The effect of 1-year transdermal estrogen replacement therapy on bone mineral density and biochemical markers of bone turnover in osteopenic postmenopausal systemic lupus erythematosus patients: a randomized, double-blind, placebo-controlled trial. *Osteoporos Int.* 2004;15(5):396-404.
17. Watts NB, Lindsay R, Li Z, Kasibhatla C, Brown J. Use of matched historical controls to evaluate the anti-fracture efficacy of once-a-week risedronate. *Osteoporos Int.* 2003;14(5):437-441.
18. Norman H Bell JPB, Henry G Bone 3rd, Amarjot Kaur, Adele Maragoto, Arthur C Santora, MK-063 Study Group. Alendronate increases bone mass and reduces bone markers in postmenopausal African-American women. *J Clin Endocrinol Metab.* 2002;87(6):2792-2797.
19. Susan L Greenspan RDE, Henry G Bone, Stuart R Weiss, Norman H Bell, Robert W Downs, Clark McKeever, Sam S Miller, Michael Davidson, Michael A Bolognese, Anthony L Mulloy, Norman Heyden, Mei Wu, Amarjot Kaur, Antonio Lombardi. Significant differential effects of alendronate, estrogen, or combination therapy on the rate of bone loss after discontinuation of treatment of postmenopausal osteoporosis. A randomized, double-blind, placebo-controlled trial. *Ann Intern Med.* 2002;137(11):875-883.
20. Susan L Greenspan NMR, Robert A Parker. Combination therapy with hormone replacement and alendronate for prevention of bone loss in elderly women: a randomized controlled trial. *JAMA.* 2003;289(19):2525-2533.
21. Grbic JT, Landesberg R, Lin SQ, et al. Incidence of osteonecrosis of the jaw in women with postmenopausal osteoporosis in the health outcomes and reduced incidence with zoledronic acid once yearly pivotal fracture trial. *J Am Dent Assoc.* 2008;139(1):32-40.
22. Chung YS, Lim SK, Chung HY, et al. Comparison of monthly ibandronate versus weekly risedronate in preference, convenience, and bone turnover markers in Korean postmenopausal osteoporotic women. *Calcif Tissue Int.* 2009;85(5):389-397.
23. Devogelaer JP, Brown JP, Burckhardt P, et al. Zoledronic acid efficacy and safety over five years in postmenopausal osteoporosis. *Osteoporos Int.* 2007;18(9):1211-1218.
24. Gallagher JC, Rosen CJ, Chen P, Misurski DA, Marcus R. Response rate of bone mineral density to teriparatide in postmenopausal women with osteoporosis. *Bone.* 2006;39(6):1268-1275.
25. Byron Cryer NB, Christine Simonelli, E Michael Lewiecki, Frank Lanza, Erluo Chen, Richard A Petruschke, Christine Mullen, Anne E de Papp. A randomized, placebo-controlled, 6-month study of once-weekly alendronate oral solution for postmenopausal osteoporosis. *Am J Geriatr Pharmacother.* 2005;3(3):127-136.
26. Iwamoto J, Takeda T, Sato Y, Uzawa M. Effects of alendronate on metacarpal and lumbar bone mineral

density, bone resorption, and chronic back pain in postmenopausal women with osteoporosis. *Clin Rheumatol.* 2004;23(5):383-389.

27. Jun Iwamoto TT, Yoshihiro Sato, Mitsuyoshi Uzawa. Determinants of one-year response of lumbar bone mineral density to alendronate treatment in elderly Japanese women with osteoporosis. *Yonsei Med J.* 2004;45(4):676-682.
28. Keaveny TM, Donley DW, Hoffmann PF, Mitlak BH, Glass EV, San Martin JA. Effects of teriparatide and alendronate on vertebral strength as assessed by finite element modeling of QCT scans in women with osteoporosis. *J Bone Miner Res.* 2007;22(1):149-157.
29. BonAdASIA Study Group; Annie Wai-Chee Kung IAR, John M F Adam, Djoko Roeshadi, Tito Torralba, Sandra Navarra, Zayda Gamilla, Arthur Cañete, Miles de la Rosa, KehSung Tsai, Hsiao-Yi Lin, Yung Kuei Soong, Joung-Liang Lan, Horng-Chaung Hsu, Shih-Te Tu, Ruey-Mo Lin, Pongsak Yuktanandana, Thawee Songpatanasilp, Srihatach Ngarmukos, Sugree Soontrapa, Suppasin Soontrapa, Sattaya Rojanasthien, Sirichai Luevitoonvechkij, Taninnit Leerapan, Adelin Albert, Sophie Vanbelle. Impact of bone marker feedback on adherence to once monthly ibandronate for osteoporosis among Asian postmenopausal women. *Int J Rheum Dis.* 2009;12(3):216-224.
30. S Ljunghall PG, O Johnell, K Larsson, E Lindh, K Obrant, I Sernbo. Synthetic human calcitonin in postmenopausal osteoporosis: a placebo-controlled, double-blind study. *Calcif Tissue Int.* 1991;49(1):17-19.
31. E G Lufkin RA, M D Whitaker, A L Cameron, V H Wong, K S Egan, W M O'Fallon, B L Riggs. Pamidronate: an unrecognized problem in gastrointestinal tolerability. *Osteoporos Int.* 1994;4(6):320-322.
32. Takafumi Majima AS, Yasato Komatsu, Noriko Satoh, Atsushi Fukao, Kiyoshi Ninomiya, Tadashi Matsumura, Kazuwa Nakao. Association between baseline values of bone turnover markers and bone mineral density and their response to raloxifene treatment in Japanese postmenopausal women with osteoporosis. *Endocr J.* 2008;55(1):41-48.
33. H E Gruber JLI, D J Baylink, M Matthews, W B Nelp, K Sisom, C H Chesnut 3rd. Long-term calcitonin therapy in postmenopausal osteoporosis. *Metabolism.* 1984;33(4):295-303.
34. P D Miller GW, A A Licata, M P Ettinger, B Mako, M E Smith, L Wang, S J Yates, M E Melton, J J Palmisano. Rechallenge of patients who had discontinued alendronate therapy because of upper gastrointestinal symptoms. *Clin Ther.* 2000;22(12):1433-1442.
35. Reid IR, Gamble GD, Mesenbrink P, Lakatos P, Black DM. Characterization of and risk factors for the acute-phase response after zoledronic acid. *J Clin Endocrinol Metab.* 2010;95(9):4380-4387.
36. B L Riggs ES, S F Hodgson, D R Taves, W M O'Fallon. Effect of the fluoride/calcium regimen on vertebral fracture occurrence in postmenopausal osteoporosis. Comparison with conventional therapy. *N Engl J Med.* 1982;306(8):446-450.
37. Stepan JJ, Burr DB, Pavo I, et al. Low bone mineral density is associated with bone microdamage accumulation in postmenopausal women with osteoporosis. *Bone.* 2007;41(3):378-385.
38. E van der Poest Clement PP, K Vandormael, H Haarman, P Lips. The effect of alendronate on bone mass after distal forearm fracture. *J Bone Miner Res.* 2000;15(3):586-593.
39. Engelke K, Nagase S, Fuerst T, et al. The effect of the cathepsin K inhibitor ONO-5334 on trabecular

and cortical bone in postmenopausal osteoporosis: the OCEAN study. *J Bone Miner Res.* 2014;29(3):629-638.

40. Yang L, Sycheva AV, Black DM, Eastell R. Site-specific differential effects of once-yearly zoledronic acid on the hip assessed with quantitative computed tomography: results from the HORIZON Pivotal Fracture Trial. *Osteoporos Int.* 2013;24(1):329-338.
41. Arcoraci V, Atteritano M, Squadrito F, et al. Antiosteoporotic Activity of Genistein Aglycone in Postmenopausal Women: Evidence from a Post-Hoc Analysis of a Multicenter Randomized Controlled Trial. *Nutrients.* 2017;9(2).
42. D Agnusdei SA, R Cervetti, G Crepaldi, O Di Munno, L Fantasia, G C Isaia, G Letizia, S Ortolani, M Passeri, et al. Effects of ipriflavone on bone mass and calcium metabolism in postmenopausal osteoporosis. *Bone Miner.* 1992;19 Suppl 1:S43-48.
43. S Gonnelli CC, M Montomoli, L Gennari, A Montagnani, R Palmieri, C Gennari. Treatment of post-menopausal osteoporosis with recombinant human growth hormone and salmon calcitonin: a placebo controlled study. *Clin Endocrinol (Oxf).* 1997;46(1):55-61.
44. Moscarini M, Patacchiola F, Spacca G, Palermo P, Caserta D, Valenti M. New perspectives in the treatment of postmenopausal osteoporosis: ipriflavone. *Gynecol Endocrinol.* 1994;8(3):203-207.
45. Nakamura T, Shiraki M, Fukunaga M, et al. Effect of the cathepsin K inhibitor odanacatib administered once weekly on bone mineral density in Japanese patients with osteoporosis--a double-blind, randomized, dose-finding study. *Osteoporos Int.* 2014;25(1):367-376.
46. M Sääf AH, M Thorën, S Troell, K Hall. Growth hormone treatment of osteoporotic postmenopausal women - a one-year placebo-controlled study. *Eur J Endocrinol.* 1999;140(5):390-399.
47. Zhang X, Li SW, Wu JF, et al. Effects of ipri fl avone on postmenopausal syndrome and osteoporosis. *Gynecol Endocrinol.* 2010;26(2):76-80.
48. Gang Zhao DC, Shufan Dong, Yu Fan. Clinical observation on treatment with the kidney-tonifying prescription in 25 cases of postmenopausal osteoporosis. *J Tradit Chin Med.* 2003;23(2):103-105.
49. Eastell R, Nagase S, Ohyama M, et al. Safety and efficacy of the cathepsin K inhibitor ONO-5334 in postmenopausal osteoporosis: the OCEAN study. *J Bone Miner Res.* 2011;26(6):1303-1312.
50. Eastell R, Nagase S, Small M, et al. Effect of ONO-5334 on bone mineral density and biochemical markers of bone turnover in postmenopausal osteoporosis: 2-year results from the OCEAN study. *J Bone Miner Res.* 2014;29(2):458-466.
51. M Cecchettin SB, G Cremonesi, L P Solimeno, G Torri. Metabolic and bone effects after administration of ipriflavone and salmon calcitonin in postmenopausal osteoporosis. *Biomed Pharmacother.* 1995;49(10):465-468.
52. Chee WS, Suriah AR, Chan SP, Zaitun Y, Chan YM. The effect of milk supplementation on bone mineral density in postmenopausal Chinese women in Malaysia. *Osteoporos Int.* 2003;14(10):828-834.
53. Y K Choi IKH, H K Yoon. Ipriflavone for the treatment of osteoporosis. *Osteoporos Int.* 1997;7 Suppl 3:S174-178.
54. A S Douglas SPR, J D Hutchison, R W Porter, A Stewart, D M Reid. Carboxylation of osteocalcin in post-menopausal osteoporotic women following vitamin K and D supplementation. *Bone.* 1995;17(1):15-20.

55. Gail A Greendale ME, Stacey Slone, Robert Marcus, Elizabeth Barrett-Connor, PEPI Safety Follow-Up Study (PSFS) Investigators. Bone mass response to discontinuation of long-term hormone replacement therapy: results from the Postmenopausal Estrogen/Progestin Interventions (PEPI) Safety Follow-up Study. *Arch Intern Med.* 2002;162(6):665-672.
56. Hampson G, Martin FC, Moffat K, et al. Effects of dietary improvement on bone metabolism in elderly underweight women with osteoporosis: a randomised controlled trial. *Osteoporos Int.* 2003;14(9):750-756.
57. Kovács AB. Efficacy of ipriflavone in the prevention and treatment of postmenopausal osteoporosis. *Agents Actions.* 1994;41(1-2):86-87.
58. Hagino H, Nishizawa Y, Sone T, et al. A double-blinded head-to-head trial of minodronate and alendronate in women with postmenopausal osteoporosis. *Bone.* 2009;44(6):1078-1084.
59. Jun Iwamoto TT, Yoshihiro Sato, Mitsuyoshi Uzawa. Comparison of effect of treatment with etidronate and alendronate on lumbar bone mineral density in elderly women with osteoporosis. *Yonsei Med J.* 2005;46(6):750-758.
60. Kushida K, Fukunaga M, Kishimoto H, et al. A comparison of incidences of vertebral fracture in Japanese patients with involutional osteoporosis treated with risedronate and etidronate: a randomized, double-masked trial. *J Bone Miner Metab.* 2004;22(5):469-478.
61. Li M, Xing XP, Zhang ZL, et al. Infusion of ibandronate once every 3 months effectively decreases bone resorption markers and increases bone mineral density in Chinese postmenopausal osteoporotic women: a 1-year study. *J Bone Miner Metab.* 2010;28(3):299-305.
62. Miller PD, Epstein S, Sedarati F, Reginster JY. Once-monthly oral ibandronate compared with weekly oral alendronate in postmenopausal osteoporosis: results from the head-to-head MOTION study. *Curr Med Res Opin.* 2008;24(1):207-213.
63. Reid DM, Hosking D, Kendler D, et al. A comparison of the effect of alendronate and risedronate on bone mineral density in postmenopausal women with osteoporosis: 24-month results from FACTS-International. *Int J Clin Pract.* 2008;62(4):575-584.
64. Sarioglu M, Tuzun C, Unlu Z, Tikiz C, Taneli F, Uyanik BS. Comparison of the effects of alendronate and risedronate on bone mineral density and bone turnover markers in postmenopausal osteoporosis. *Rheumatol Int.* 2006;26(3):195-200.
65. Tankó LB, McClung MR, Schimmer RC, Mahoney P, Christiansen C. The efficacy of 48-week oral ibandronate treatment in postmenopausal osteoporosis when taken 30 versus 60 minutes before breakfast. *Bone.* 2003;32(4):421-426.
66. R Balena MK, J A Foldes, M S Shih, D S Rao, H C Schober, A M Parfitt. Effects of different regimens of sodium fluoride treatment for osteoporosis on the structure, remodeling and mineralization of bone. *Osteoporos Int.* 1998;8(5):428-435.
67. Suzuki T, Nakamura Y, Kato H. Efficacy, safety, and compliance of ibandronate treatment for 3 years in postmenopausal Japanese women with primary osteoporosis. *Osteoporos Sarcopenia.* 2018;4(2):69-72.
68. A Gürlek MB, O Gedik. Comparison of calcitriol treatment with etidronate-calcitriol and calcitonin-calcitriol combinations in Turkish women with postmenopausal osteoporosis: a prospective study. *Calcif Tissue Int.* 1997;61(1):39-43.

69. Lindsay R, Nieves J, Formica C, et al. Randomised controlled study of effect of parathyroid hormone on vertebral-bone mass and fracture incidence among postmenopausal women on oestrogen with osteoporosis. *The Lancet.* 1997;350(9077):550-555.
70. Orimo H, Nakamura T, Fukunaga M, et al. Effects of alendronate plus alfacalcidol in osteoporosis patients with a high risk of fracture: the Japanese Osteoporosis Intervention Trial (JOINT) - 02. *Curr Med Res Opin.* 2011;27(6):1273-1284.
71. Reid IR, Cundy T, Grey AB, et al. Addition of monofluorophosphate to estrogen therapy in postmenopausal osteoporosis: a randomized controlled trial. *J Clin Endocrinol Metab.* 2007;92(7):2446-2452.
72. Ringe JD, Setnikar I. Monofluorophosphate combined with hormone replacement therapy in postmenopausal osteoporosis. An open-label pilot efficacy and safety study. *Rheumatol Int.* 2002;22(1):27-32.
73. Deal C, Omizo M, Schwartz EN, et al. Combination teriparatide and raloxifene therapy for postmenopausal osteoporosis: results from a 6-month double-blind placebo-controlled trial. *J Bone Miner Res.* 2005;20(11):1905-1911.
74. D H Gutteridge GOS, R L Prince, R I Price, R W Retallack, S S Dhaliwal, B G A Stuckey, P Drury, C E Jones, D L Faulkner, G N Kent, C I Bhagat, G C Nicholson, K Jamrozik. A randomized trial of sodium fluoride (60 mg) +/- estrogen in postmenopausal osteoporotic vertebral fractures: increased vertebral fractures and peripheral bone loss with sodium fluoride; concurrent estrogen prevents peripheral loss, but not vertebral fractures. *Osteoporos Int.* 2002;13(2):158-170.
75. S T Harris EFE, M Davidson, M P Ettinger, A H Moffett Jr, D J Baylink, C E Crusan, A A Chines. Effect of combined risedronate and hormone replacement therapies on bone mineral density in postmenopausal women. *J Clin Endocrinol Metab.* 2001;86(5):1890-1897.
76. A B Hodsman LJF, P H Watson, T Ostbye, L W Stitt, J D Adachi, D H Taves, D Drost. A randomized controlled trial to compare the efficacy of cyclical parathyroid hormone versus cyclical parathyroid hormone and sequential calcitonin to improve bone mass in postmenopausal women with osteoporosis. *J Clin Endocrinol Metab.* 1997;82(2):620-628.
77. R Lindsay FC, R A Lobo, B W Walsh, S T Harris, J E Reagan, C L Liss, M E Melton, C A Byrnes. Addition of alendronate to ongoing hormone replacement therapy in the treatment of osteoporosis: a randomized, controlled clinical trial. *J Clin Endocrinol Metab.* 1999;84(9):3076-3081.
78. K Sakhaee AZ, J R Poindexter, J E Zerwekh, C Y Pak. Metabolic effects of thiazide and 1,25-(OH)2 vitamin D in postmenopausal osteoporosis. *Osteoporos Int.* 1993;3(4):209-214.
79. Sone T, Ito M, Fukunaga M, et al. The effects of once-weekly teriparatide on hip geometry assessed by hip structural analysis in postmenopausal osteoporotic women with high fracture risk. *Bone.* 2014;64:75-81.
80. Duvernoy CS, Kulkarni PM, Dowsett SA, Keech CA. Vascular events in the Multiple Outcomes of Raloxifene Evaluation (MORE) trial: incidence, patient characteristics, and effect of raloxifene. *Menopause.* 2005;12(4):444-452.
81. Akira Itabashi KY, Arkadi A Chines, Takami Miki, Masahiko Takada, Hiroshi Sato, Itsuo Gorai, Toshitsugu Sugimoto, Hideki Mizunuma, Hiroshi Ochi, Ginger D Constantine, Hiroaki Ohta. Bridging

analysis of the efficacy and safety of bazedoxifene in Japanese and global populations of postmenopausal women with osteoporosis. *J Bone Miner Metab.* 2015;33(1):61-72.
82. Robert Marcus OW, Julie Satterwhite, Bruce Mitlak. The skeletal response to teriparatide is largely independent of age, initial bone mineral density, and prevalent vertebral fractures in postmenopausal women with osteoporosis. *J Bone Miner Res.* 2003;18(1):18-23.
83. Dempster DW, Brown JP, Fahrleitner-Pammer A, et al. Effects of Long-Term Denosumab on Bone Histomorphometry and Mineralization in Women With Postmenopausal Osteoporosis. *J Clin Endocrinol Metab.* 2018;103(7):2498-2509.
84. Reginster JY, Bruyere O, Sawicki A, et al. Long-term treatment of postmenopausal osteoporosis with strontium ranelate: results at 8 years. *Bone.* 2009;45(6):1059-1064.
85. Reginster JY, Kaufman JM, Goemaere S, et al. Maintenance of antifracture efficacy over 10 years with strontium ranelate in postmenopausal osteoporosis. *Osteoporos Int.* 2012;23(3):1115-1122.
86. Zanchetta JR, Bogado CE, Cisari C, et al. Treatment of postmenopausal women with osteoporosis with PTH(1-84) for 36 months: treatment extension study. *Curr Med Res Opin.* 2010;26(11):2627-2633.
87. Kanis JA, Barton IP, Johnell O. Risedronate decreases fracture risk in patients selected solely on the basis of prior vertebral fracture. *Osteoporos Int.* 2005;16(5):475-482.
88. Harrington JT, Ste-Marie LG, Brandi ML, et al. Risedronate rapidly reduces the risk for nonvertebral fractures in women with postmenopausal osteoporosis. *Calcif Tissue Int.* 2004;74(2):129-135.
89. Sorensen OH, Crawford GM, Mulder H, et al. Long-term efficacy of risedronate: a 5-year placebo-controlled clinical experience. *Bone.* 2003;32(2):120-126.
90. Watts NB, Josse RG, Hamdy RC, et al. Risedronate prevents new vertebral fractures in postmenopausal women at high risk. *J Clin Endocrinol Metab.* 2003;88(2):542-549.
91. Goldstein SR, Nanavati N. Adverse events that are associated with the selective estrogen receptor modulator levormeloxifene in an aborted phase III osteoporosis treatment study. *Am J Obstet Gynecol.* 2002;187(3):521-527.
92. Michael Maricic JDA, Somnath Sarkar, Wentao Wu, Mayme Wong, Kristine D Harper. Early effects of raloxifene on clinical vertebral fractures at 12 months in postmenopausal women with osteoporosis. *Arch Intern Med.* 2002;162(10):1140-1143.
93. Liao EY, Zhang ZL, Xia WB, et al. Calcifediol (25-hydroxyvitamin D) improvement and calcium-phosphate metabolism of alendronate sodium/vitamin D3 combination in Chinese women with postmenopausal osteoporosis: a post hoc efficacy analysis and safety reappraisal. *BMC Musculoskelet Disord.* 2018;19(1):210.
94. Watts NB, Grbic JT, Binkley N, et al. Invasive Oral Procedures and Events in Postmenopausal Women With Osteoporosis Treated With Denosumab for Up to 10 Years. *J Clin Endocrinol Metab.* 2019;104(6):2443-2452.
95. Sugimoto T, Shiraki M, Nakano T, et al. A randomized, double-blind, placebo-controlled study of once weekly elcatonin in primary postmenopausal osteoporosis. *Curr Med Res Opin.* 2019;35(3):447-454.
96. Kendler DL, Marin F, Zerbini CAF, et al. Effects of teriparatide and risedronate on new fractures in post-menopausal women with severe osteoporosis (VERO): a multicentre, double-blind, double-dummy, randomised controlled trial. *The Lancet.* 2018;391(10117):230-240.

97. Tsai JN, Burnett-Bowie SM, Lee H, Leder BZ. Relationship between bone turnover and density with teriparatide, denosumab or both in women in the DATA study. *Bone.* 2017;95:20-25.
98. Saag KG, Petersen J, Brandi ML, et al. Romosozumab or Alendronate for Fracture Prevention in Women with Osteoporosis. *N Engl J Med.* 2017;377(15):1417-1427.
99. Liang BC, Shi ZY, Wang B, et al. Intravenous Zoledronic Acid 5 mg on Bone Turnover Markers and Bone Mineral Density in East China Subjects with Newly Diagnosed Osteoporosis: A 24-month Clinical Study. *Orthop Surg.* 2017;9(1):103-109.
100. Koh JM, Chung DJ, Chung YS, et al. Assessment of Denosumab in Korean Postmenopausal Women with Osteoporosis: Randomized, Double-Blind, Placebo-Controlled Trial with Open-Label Extension. *Yonsei Med J.* 2016;57(4):905-914.
101. Henriksen K, Byrjalsen I, Andersen JR, et al. A randomized, double-blind, multicenter, placebo-controlled study to evaluate the efficacy and safety of oral salmon calcitonin in the treatment of osteoporosis in postmenopausal women taking calcium and vitamin D. *Bone.* 2016;91:122-129.
102. Cosman F, Crittenden DB, Adachi JD, et al. Romosozumab Treatment in Postmenopausal Women with Osteoporosis. *N Engl J Med.* 2016;375(16):1532-1543.
103. Miller PD, Hattersley G, Riis BJ, et al. Effect of Abaloparatide vs Placebo on New Vertebral Fractures in Postmenopausal Women With Osteoporosis: A Randomized Clinical Trial. *JAMA.* 2016;316(7):722-733.
104. Zhang ZL, Liao EY, Xia WB, et al. Alendronate sodium/vitamin D3 combination tablet versus calcitriol for osteoporosis in Chinese postmenopausal women: a 6-month, randomized, open-label, active-comparator-controlled study with a 6-month extension. *Osteoporos Int.* 2015;26(9):2365-2374.
105. Santiago Palacios SLS, Tobie J de Villiers, Amy B Levine, Stefan Goemaere, Jacques P Brown, Fiorenzo De Cicco Nardone, Robert Williams, Teresa L Hines, Sebastian Mirkin, Arkadi A Chines, Bazedoxifene Study Group. A 7-year randomized, placebo-controlled trial assessing the long-term efficacy and safety of bazedoxifene in postmenopausal women with osteoporosis. *Menopause.* 2015;22(8):806-813.
106. Leder BZ, O'Dea LS, Zanchetta JR, et al. Effects of abaloparatide, a human parathyroid hormone-related peptide analog, on bone mineral density in postmenopausal women with osteoporosis. *J Clin Endocrinol Metab.* 2015;100(2):697-706.
107. Leder BZ, Tsai JN, Uihlein AV, et al. Two years of Denosumab and teriparatide administration in postmenopausal women with osteoporosis (The DATA Extension Study): a randomized controlled trial. *J Clin Endocrinol Metab.* 2014;99(5):1694-1700.
108. Lola S Abboskhujaeva SII, Nodira M Alikhanova. Efficacy of strontium ranelate in combination with a D-hormone analog for the treatment of postmenopausal osteoporosis. *Drugs R D.* 2014;14(4):315-324.
109. Henriksen K, Andersen JR, Riis BJ, et al. Evaluation of the efficacy, safety and pharmacokinetic profile of oral recombinant human parathyroid hormone [rhPTH(1-31)NH(2)] in postmenopausal women with osteoporosis. *Bone.* 2013;53(1):160-166.
110. Rizzoli R, Chapurlat RD, Laroche JM, et al. Effects of strontium ranelate and alendronate on bone microstructure in women with osteoporosis. Results of a 2-year study. *Osteoporos Int.* 2012;23(1):305-315.

111. Zhang L, Yang M, Liu D, Guo C, Li L, Yang G. The rhPTH (1-34), but not elcatonin, increases bone anabolic efficacy in postmenopausal women with osteoporosis. *Exp Clin Endocrinol Diabetes.* 2012;120(6):361-366.
112. Binkley N, Bolognese M, Sidorowicz-Bialynicka A, et al. A phase 3 trial of the efficacy and safety of oral recombinant calcitonin: the Oral Calcitonin in Postmenopausal Osteoporosis (ORACAL) trial. *J Bone Miner Res.* 2012;27(8):1821-1829.
113. Cosman F, Eriksen EF, Recknor C, et al. Effects of intravenous zoledronic acid plus subcutaneous teriparatide [rhPTH(1-34)] in postmenopausal osteoporosis. *J Bone Miner Res.* 2011;26(3):503-511.
114. Tuppurainen M, Harma K, Komulainen M, et al. Effects of continuous combined hormone replacement therapy and clodronate on bone mineral density in osteoporotic postmenopausal women: a 5-year follow-up. *Maturitas.* 2010;66(4):423-430.
115. Finkelstein JS, Wyland JJ, Lee H, Neer RM. Effects of teriparatide, alendronate, or both in women with postmenopausal osteoporosis. *J Clin Endocrinol Metab.* 2010;95(4):1838-1845.
116. Ensrud K, LaCroix A, Thompson JR, et al. Lasofoxifene and cardiovascular events in postmenopausal women with osteoporosis: Five-year results from the Postmenopausal Evaluation and Risk Reduction with Lasofoxifene (PEARL) trial. *Circulation.* 2010;122(17):1716-1724.
117. Steven R. Cummings KE, Pierre D. Delmas, Andrea Z. LaCroix, Slobodan Vukicevic, David M. Reid, Ch.B., Steven Goldstein, Usha Sriram, Andy Lee, John Thompson, Roisin A. Armstrong, David D. Thompson, Trevor Powles, Jose Zanchetta, David Kendler, Patrick Neven, and Richard Eastell. Lasofoxifene in Postmenopausal Women with Osteoporosis. *N Engl J Med* 2010;362:686-696.
118. LaCroix AZ, Powles T, Osborne CK, et al. Breast cancer incidence in the randomized PEARL trial of lasofoxifene in postmenopausal osteoporotic women. *J Natl Cancer Inst.* 2010;102(22):1706-1715.
119. Claus Christiansen CHCr, Jonathan D Adachi, Jacques P Brown, César E Fernandes, Annie Wc Kung, Santiago Palacios, Amy B Levine, Arkadi A Chines, Ginger D Constantine. Safety of bazedoxifene in a randomized, double-blind, placebo- and active-controlled Phase 3 study of postmenopausal women with osteoporosis. *BMC Musculoskelet Disord.* 2010;11:130.
120. Yuxiang Yan WW, Hanmin Zhu, Mei Li, Jianli Liu, Bangyao Luo, Haibao Xie, Guangjian Zhang, Fuobao Li. The efficacy and tolerability of once-weekly alendronate 70 mg on bone mineral density and bone turnover markers in postmenopausal Chinese women with osteoporosis. *J Bone Miner Metab.* 2009;27(4):471-478.
121. Steven R Cummings JSM, Michael R McClung, Ethel S Siris, Richard Eastell, Ian R Reid, Pierre Delmas, Holly B Zoog, Matt Austin, Andrea Wang, Stepan Kutilek, Silvano Adami, Jose Zanchetta, Cesar Libanati, Suresh Siddhanti, Claus Christiansen. Denosumab for prevention of fractures in postmenopausal women with osteoporosis. *N Engl J Med.* 2009;361(8):756-765.
122. Silverman SL, Christiansen C, Genant HK, et al. Efficacy of bazedoxifene in reducing new vertebral fracture risk in postmenopausal women with osteoporosis: results from a 3-year, randomized, placebo-, and active-controlled clinical trial. *J Bone Miner Res.* 2008;23(12):1923-1934.
123. Iwamoto J, Sato Y, Uzawa M, Takeda T, Matsumoto H. Comparison of effects of alendronate and raloxifene on lumbar bone mineral density, bone turnover, and lipid metabolism in elderly women with osteoporosis. *Yonsei Med J.* 2008;49(1):119-128.

124. Miyauchi A, Matsumoto T, Shigeta H, Tsujimoto M, Thiebaud D, Nakamura T. Effect of teriparatide on bone mineral density and biochemical markers in Japanese women with postmenopausal osteoporosis: a 6-month dose-response study. *J Bone Miner Metab.* 2008;26(6):624-634.
125. Hwang JS, Chen JF, Yang TS, et al. The effects of strontium ranelate in Asian women with postmenopausal osteoporosis. *Calcif Tissue Int.* 2008;83(5):308-314.
126. B K Sethi MC, K D Modi, K M Prasanna Kumar, R Mehrotra, Usha Sriram. Efficacy of teriparatide in increasing bone mineral density in postmenopausal women with osteoporosis--an Indian experience. *J Assoc Physicians India.* 2008;56:418-424.
127. Susan L Greenspan HGB, Mark P Ettinger, David A Hanley, Robert Lindsay, Jose R Zanchetta, Consuelo M Blosch, Annette L Mathisen, Stephen A Morris, Thomas B Marriott. Effect of recombinant human parathyroid hormone (1-84) on vertebral fracture and bone mineral density in postmenopausal women with osteoporosis: a randomized trial. *Ann Intern Med.* 2007;146(5):326-339.
128. Dennis M Black PDD, Richard Eastell, Ian R Reid, Steven Boonen, Jane A Cauley, Felicia Cosman, Péter Lakatos, Ping Chung Leung, Zulema Man, Carlos Mautalen, Peter Mesenbrink, Huilin Hu, John Caminis, Karen Tong, Theresa Rosario-Jansen, Joel Krasnow, Trisha F Hue, Deborah Sellmeyer, Erik Fink Eriksen, Steven R Cummings. Once-yearly zoledronic acid for treatment of postmenopausal osteoporosis. *N Engl J Med.* 2007;356(18):1809-1822.
129. Refik Tanakol SY, Taner Bayraktaroglu, Harika Boztepe, Faruk Alagöl. Clodronic acid in the treatment of postmenopausal osteoporosis. *Clin Drug Investig.* 2007;27(6):419-433.
130. Kung AW, Pasion EG, Sofiyan M, et al. A comparison of teriparatide and calcitonin therapy in postmenopausal Asian women with osteoporosis: a 6-month study. *Curr Med Res Opin.* 2006;22(5):929-937.
131. Hwang JS, Tu ST, Yang TS, Chen JF, Wang CJ, Tsai KS. Teriparatide vs. calcitonin in the treatment of Asian postmenopausal women with established osteoporosis. *Osteoporos Int.* 2006;17(3):373-378.
132. Gonnelli S, Martini G, Caffarelli C, et al. Teriparatide's effects on quantitative ultrasound parameters and bone density in women with established osteoporosis. *Osteoporos Int.* 2006;17(10):1524-1531.
133. Ensrud K, Genazzani AR, Geiger MJ, et al. Effect of raloxifene on cardiovascular adverse events in postmenopausal women with osteoporosis. *Am J Cardiol.* 2006;97(4):520-527.
134. Martino S, Disch D, Dowsett SA, Keech CA, Mershon JL. Safety assessment of raloxifene over eight years in a clinical trial setting. *Curr Med Res Opin.* 2005;21(9):1441-1452.
135. Michael R McClung JSM, Paul D Miller, Roberto Civitelli, Francisco Bandeira, Molly Omizo, David W Donley, Gail P Dalsky, Erik F Eriksen. Opposite bone remodeling effects of teriparatide and alendronate in increasing bone mass. *Arch Intern Med.* 2005;165(15):1762-1768.
136. Leung JY, Ho AY, Ip TP, Lee G, Kung AW. The efficacy and tolerability of risedronate on bone mineral density and bone turnover markers in osteoporotic Chinese women: a randomized placebo-controlled study. *Bone.* 2005;36(2):358-364.
137. Ho AY, Kung AW. Efficacy and tolerability of alendronate once weekly in Asian postmenopausal osteoporotic women. *Ann Pharmacother.* 2005;39(9):1428-1433.
138. Ligia J Dominguez AG, Anna Ferlisi, Maria Adele Alessi, Mario Belvedere, Ernesto Putignano, Giuseppe Costanza, Maurizio Bevilacqua, Mario Barbagallo. Intermittent intramuscular clodronate

therapy: a valuable option for older osteoporotic women. *Age Ageing.* 2005;34(6):633-636.
139. Yuming Li ZZ, Xiuling Deng, Lulu Chen. Efficacy and safety of risedronate sodium in treatment of postmenopausal osteoporosis. *J Huazhong Univ Sci Technolog Med Sci.* 2005;25(5):527-529.
140. Kushida K, Shiraki M, Nakamura T, et al. Alendronate reduced vertebral fracture risk in postmenopausal Japanese women with osteoporosis: a 3-year follow-up study. *J Bone Miner Metab.* 2004;22(5):462-468.
141. Ishida Y, Kawai S. Comparative efficacy of hormone replacement therapy, etidronate, calcitonin, alfacalcidol, and vitamin K in postmenopausal women with osteoporosis: The Yamaguchi Osteoporosis Prevention Study. *Am J Med.* 2004;117(8):549-555.
142. Adami S, Felsenberg D, Christiansen C, et al. Efficacy and safety of ibandronate given by intravenous injection once every 3 months. *Bone.* 2004;34(5):881-889.
143. Ste-Marie LG, Sod E, Johnson T, Chines A. Five years of treatment with risedronate and its effects on bone safety in women with postmenopausal osteoporosis. *Calcif Tissue Int.* 2004;75(6):469-476.
144. Pierre J Meunier CR, Ego Seeman, Sergio Ortolani, Janusz E Badurski, Tim D Spector, Jorge Cannata, Adam Balogh, Ernst-Martin Lemmel, Stig Pors-Nielsen, René Rizzoli, Harry K Genant, Jean-Yves Reginster. The effects of strontium ranelate on the risk of vertebral fracture in women with postmenopausal osteoporosis. *N Engl J Med.* 2004;350(5):459-468.
145. Luckey M, Kagan R, Greenspan S, et al. Once-weekly alendronate 70 mg and raloxifene 60 mg daily in the treatment of postmenopausal osteoporosis. *Menopause.* 2004;11(4):405-415.
146. Genant HK, Lang T, Fuerst T, et al. Treatment with raloxifene for 2 years increases vertebral bone mineral density as measured by volumetric quantitative computed tomography. *Bone.* 2004;35(5):1164-1168.
147. Steven Boonen MRM, Richard Eastell, Ghada El-Hajj Fuleihan, Ian P Barton, Pierre Delmas. Safety and efficacy of risedronate in reducing fracture risk in osteoporotic women aged 80 and older: implications for the use of antiresorptive agents in the old and oldest old. *J Am Geriatr Soc.* 2004;52(11):1832-1839.
148. Barrett-Connor E, Cauley JA, Kulkarni PM, Sashegyi A, Cox DA, Geiger MJ. Risk-benefit profile for raloxifene: 4-year data From the Multiple Outcomes of Raloxifene Evaluation (MORE) randomized trial. *J Bone Miner Res.* 2004;19(8):1270-1275.
149. Martino S, Cauley JA, Barrett-Connor E, et al. Continuing outcomes relevant to Evista: breast cancer incidence in postmenopausal osteoporotic women in a randomized trial of raloxifene. *J Natl Cancer Inst.* 2004;96(23):1751-1761.
150. Morii H, Ohashi Y, Taketani Y, et al. Effect of raloxifene on bone mineral density and biochemical markers of bone turnover in Japanese postmenopausal women with osteoporosis: results from a randomized placebo-controlled trial. *Osteoporos Int.* 2003;14(10):793-800.
151. Braga V, Gatti D, Colapietro F, et al. Intravenous intermittent neridronate in the treatment of postmenopausal osteoporosis. *Bone.* 2003;33(3):342-345.
152. Hodsman AB, Hanley DA, Ettinger MP, et al. Efficacy and safety of human parathyroid hormone-(1-84) in increasing bone mineral density in postmenopausal osteoporosis. *J Clin Endocrinol Metab.* 2003;88(11):5212-5220.

153. Chailurkit LO, Jongjaroenprasert W, Rungbunnapun S, Ongphiphadhanakul B, Sae-tung S, Rajatanavin R. Effect of alendronate on bone mineral density and bone turnover in Thai postmenopausal osteoporosis. *J Bone Miner Metab.* 2003;21(6):421-427.
154. P J Meunier DOS, P D Delmas, J L Sebert, M L Brandi, C Albanese, R Lorenc, S Pors-Nielsen, M C De Vernejoul, A Roces, J Y Reginster. Strontium ranelate: dose-dependent effects in established postmenopausal vertebral osteoporosis--a 2-year randomized placebo controlled trial. *J Clin Endocrinol Metab.* 2002;87(5):2060-2066.
155. Olof Johnell WHS, Yili Lu, Jean-Yves Reginster, Allan G Need, Ego Seeman. Additive effects of raloxifene and alendronate on bone density and biochemical markers of bone remodeling in postmenopausal women with osteoporosis. *J Clin Endocrinol Metab.* 2002;87(3):985-992.
156. Body JJ, Gaich GA, Scheele WH, et al. A randomized double-blind trial to compare the efficacy of teriparatide [recombinant human parathyroid hormone (1-34)] with alendronate in postmenopausal women with osteoporosis. *J Clin Endocrinol Metab.* 2002;87(10):4528-4535.
157. C D Rubin CYP, B Adams-Huet, H K Genant, J Li, D S Rao. Sustained-release sodium fluoride in the treatment of the elderly with established osteoporosis. *Arch Intern Med.* 2001;161(19):2325-2333.
158. B J Riis JI, T von Stein, Y Bagger, C Christiansen. Ibandronate: a comparison of oral daily dosing versus intermittent dosing in postmenopausal osteoporosis. *J Bone Miner Res.* 2001;16(10):1871-1878.
159. R M Neer CDA, J R Zanchetta, R Prince, G A Gaich, J Y Reginster, A B Hodsman, E F Eriksen, S Ish-Shalom, H K Genant, O Wang, B H Mitlak. Effect of parathyroid hormone (1-34) on fractures and bone mineral density in postmenopausal women with osteoporosis. *N Engl J Med.* 2001;344(19):1434-1441.
160. J Iwamoto TT, S Ichimura. Effect of menatetrenone on bone mineral density and incidence of vertebral fractures in postmenopausal women with osteoporosis: a comparison with the effect of etidronate. *J Orthop Sci.* 2001;6(6):487-492.
161. N Guañabens JF, L Perez-Edo, A Monegal, A Renau, J Carbonell, M Roca, M Torra, M Pavesi. Cyclical etidronate versus sodium fluoride in established postmenopausal osteoporosis: a randomized 3 year trial. *Bone.* 2000;27(1):123-128.
162. H G Bone SLG, C McKeever, N Bell, M Davidson, R W Downs, R Emkey, P J Meunier, S S Miller, A L Mulloy, R R Recker, S R Weiss, N Heyden, T Musliner, S Suryawanshi, A J Yates, A Lombardi. Alendronate and estrogen effects in postmenopausal women with low bone mineral density. Alendronate/Estrogen Study Group. *J Clin Endocrinol Metab.* 2000;85(2):720-726.
163. O Sahota IF, P J Blackwell, N Lawson, S A Cawte, P San, T Masud, D J Hosking. A comparison of continuous alendronate, cyclical alendronate and cyclical etidronate with calcitriol in the treatment of postmenopausal vertebral osteoporosis: a randomized controlled trial. *Osteoporos Int.* 2000;11(11):959-966.
164. R W Downs Jr NHB, M P Ettinger, B W Walsh, M J Favus, B Mako, L Wang, M E Smith, G J Gormley, M E Melton. Comparison of alendronate and intranasal calcitonin for treatment of osteoporosis in postmenopausal women. *J Clin Endocrinol Metab.* 2000;85(5):1783-1788.
165. C H Chesnut SS, K Andriano, H Genant, A Gimona, S Harris, D Kiel, M LeBoff, M Maricic, P Miller, C Moniz, M Peacock, P Richardson, N Watts, D Baylink. A randomized trial of nasal spray salmon calcitonin in postmenopausal women with established osteoporosis: the prevent recurrence of

osteoporotic fractures study. PROOF Study Group. *Am J Med.* 2000;109(4):267-276.
166. G Y Boivin PMC, A C Santora, J Yates, P J Meunier. Alendronate increases bone strength by increasing the mean degree of mineralization of bone tissue in osteoporotic women. *Bone.* 2000;27(5):687-694.
167. J Reginster HWM, O H Sorensen, M Hooper, C Roux, M L Brandi, B Lund, D Ethgen, S Pack, I Roumagnac, R Eastell. Randomized trial of the effects of risedronate on vertebral fractures in women with established postmenopausal osteoporosis. *Osteoporos Int.* 2000;11(1):83-91.
168. Kung AWC, Yeung SSC, Chu LW. The Efficacy and Tolerability of Alendronate in Postmenopausal Osteoporotic Chinese Women: A Randomized Placebo-Controlled Study. *Calcified Tissue International.* 2000;67(4):286-290.
169. M B Tiraş VN, A Yildiz, M Yildirim, S Daya. Effects of alendronate and hormone replacement therapy, alone or in combination, on bone mass in postmenopausal women with osteoporosis: a prospective, randomized study. *Hum Reprod.* 2000;15(10):2087-2092.
170. M Rossini VB, D Gatti, D Gerardi, N Zamberlan, S Adami. Intramuscular clodronate therapy in postmenopausal osteoporosis. *Bone.* 1999;24(2):125-129.
171. J D Ringe CK, A Cöster, R Umbach. Therapy of established postmenopausal osteoporosis with monofluorophosphate plus calcium: dose-related effects on bone density and fracture rate. *Osteoporos Int.* 1999;9(2):171-178.
172. S T Harris NBW, H K Genant, C D McKeever, T Hangartner, M Keller, C H Chesnut 3rd, J Brown, E F Eriksen, M S Hoseyni, D W Axelrod, P D Miller. Effects of risedronate treatment on vertebral and nonvertebral fractures in women with postmenopausal osteoporosis: a randomized controlled trial. *JAMA.* 1999;282(14):1344-1352.
173. J Y Reginster LM, B Zegels, L C Rovati, H W Minne, G Giacovelli, A N Taquet, I Setnikar, J Collette, C Gosset. The effect of sodium monofluorophosphate plus calcium on vertebral fracture rate in postmenopausal women with moderate osteoporosis. A randomized, controlled trial. *Ann Intern Med.* 1998;129(1):1-8.
174. P J Meunier JLS, J Y Reginster, D Briancon, T Appelboom, P Netter, G Loeb, A Rouillon, S Barry, J C Evreux, B Avouac, X Marchandise. Fluoride salts are no better at preventing new vertebral fractures than calcium-vitamin D in postmenopausal osteoporosis: the FAVOStudy. *Osteoporos Int.* 1998;8(1):4-12.
175. E G Lufkin MDW, T Nickelsen, R Argueta, R H Caplan, R K Knickerbocker, B L Riggs. Treatment of established postmenopausal osteoporosis with raloxifene: a randomized trial. *J Bone Miner Res.* 1998;13(11):1747-1754.
176. D Felsenberg FA, O Bock, C Hammermeister, W Gowan. Placebo-controlled multicenter study of oral alendronate in postmenopausal osteoporotic women. *Maturitas.* 1998;31(1):35-44.
177. S Gonnelli CC, C Pondrelli, S Martini, R Monaco, C Gennari. The usefulness of bone turnover in predicting the response to transdermal estrogen therapy in postmenopausal osteoporosis. *J Bone Miner Res.* 1997;12(4):624-631.
178. D Thiébaud PB, H Kriegbaum, H Huss, H Mulder, J R Juttmann, K H Schöter. Three monthly intravenous injections of ibandronate in the treatment of postmenopausal osteoporosis. *Am J Med.* 1997;103(4):298-307.

179. P Filipponi SC, E Rizzello, G Policani, L Fedeli, F Gregorio, S Boldrini, S Troiani, C Massoni. Cyclical intravenous clodronate in postmenopausal osteoporosis: results of a long-term clinical trial. *Bone.* 1996;18(2):179-184.

180. J R Tucci RPT, R D Emkey, C A Peverly, U Kher, A C Santora 2nd. Effect of three years of oral alendronate treatment in postmenopausal women with osteoporosis. *Am J Med.* 1996;101(5):488-501.

181. P Ravn BC, B J Riis, C Christiansen. The effect on bone mass and bone markers of different doses of ibandronate: a new bisphosphonate for prevention and treatment of postmenopausal osteoporosis: a 1-year, randomized, double-blind, placebo-controlled dose-finding study. *Bone.* 1996;19(5):527-533.

182. M C Ellerington TCH, S I Whitcroft, M S Marsh, B Lees, L M Banks, M I Whitehead, J C Stevenson. Intranasal salmon calcitonin for the prevention and treatment of postmenopausal osteoporosis. *Calcif Tissue Int.* 1996;59(1):6-11.

183. J P Devogelaer HB, R Correa-Rotter, D C Cumming, C N De Deuxchaisnes, P Geusens, D Hosking, P Jaeger, J M Kaufman, M Leite, J Leon, U Liberman, C J Menkes, P J Meunier, I Reid, J Rodriguez, A Romanowicz, E Seeman, A Vermeulen, L J Hirsch, A Lombardi, K Plezia, A C Santora, A J Yates, W Yuan. Oral alendronate induces progressive increases in bone mass of the spine, hip, and total body over 3 years in postmenopausal women with osteoporosis. *Bone.* 1996;18(2):141-150.

184. C Y Pak KS, B Adams-Huet, V Piziak, R D Peterson, J R Poindexter. Treatment of postmenopausal osteoporosis with slow-release sodium fluoride. Final report of a randomized controlled trial. *Ann Intern Med.* 1995;123(6):401-408.

185. U A Liberman SRW, J Bröll, H W Minne, H Quan, N H Bell, J Rodriguez-Portales, R W Downs Jr, J Dequeker, M Favus. Effect of oral alendronate on bone mineral density and the incidence of fractures in postmenopausal osteoporosis. *N Engl J Med.* 1995;333(22):1437-1443.

186. C H Chesnut 3rd MRM, K E Ensrud, N H Bell, H K Genant, S T Harris, F R Singer, J L Stock, R A Yood, P D Delmas, et al. Alendronate treatment of the postmenopausal osteoporotic woman: effect of multiple dosages on bone mass and bone remodeling. *Am J Med.* 1995;99(2):144-152.

187. S Adami MP, S Ortolani, M Broggini, L Carratelli, I Caruso, G Gandolini, L Gnessi, M Laurenzi, A Lombardi, et al. Effects of oral alendronate and intranasal salmon calcitonin on bone mass and biochemical markers of bone turnover in postmenopausal women with osteoporosis. *Bone.* 1995;17(4):383-390.

188. D Thiébaud PB, J Melchior, P Eckert, A F Jacquet, P Schnyder, C Gobelet. Two years' effectiveness of intravenous pamidronate (APD) versus oral fluoride for osteoporosis occurring in the postmenopause. *Osteoporos Int.* 1994;4(2):76-83.

189. I R Reid DJW, M C Evans, G D Gamble, J P Stapleton, J Cornish. Continuous therapy with pamidronate, a potent bisphosphonate, in postmenopausal osteoporosis. *J Clin Endocrinol Metab.* 1994;79(6):1595-1599.

190. M Rossini DG, N Zamberlan, V Braga, R Dorizzi, S Adami. Long-term effects of a treatment course with oral alendronate of postmenopausal osteoporosis. *J Bone Miner Res.* 1994;9(11):1833-1837.

191. S Adami MCB, M Broggini, L Carratelli, I Caruso, L Gnessi, M Laurenzi, A Lombardi, G Norbiato, S Ortolani, et al. Treatment of postmenopausal osteoporosis with continuous daily oral alendronate in comparison with either placebo or intranasal salmon calcitonin. *Osteoporos Int.* 1993;3 Suppl 3:S21-27.
